# The evolutionary trajectory of the mating-type (*mat*) genes in *Neurospora *relates to reproductive behavior of taxa

**DOI:** 10.1186/1471-2148-8-109

**Published:** 2008-04-11

**Authors:** Lotta Wik, Magnus Karlsson, Hanna Johannesson

**Affiliations:** 1 Swedish University of Agricultural Sciences, Department of Biomedical Sciences and Veterinary Public Health, Husargatan 3, SE-75123 Uppsala, Sweden; 2 Swedish University of Agricultural Sciences, Department of Forest Mycology and Pathology, Ulls väg 26, SE-750 07 Uppsala, Sweden; 3 Uppsala University, Department of Evolutionary Biology, Norbyvägen 18D, SE-752 36 Uppsala, Sweden

## Abstract

**Background:**

Comparative sequencing studies among a wide range of taxonomic groups, including fungi, have led to the discovery that reproductive genes evolve more rapidly than other genes. However, for fungal reproductive genes the question has remained whether the rapid evolution is a result of stochastic or deterministic processes. The mating-type (*mat*) genes constitute the master regulators of sexual reproduction in filamentous ascomycetes and here we present a study of the molecular evolution of the four *mat*-genes (*mat a-1*, *mat A-1*, *mat A-2 *and *mat A-3*) of 20 *Neurospora *taxa.

**Results:**

We estimated nonsynonymous and synonymous substitution rates of genes to infer their evolutionary rate, and confirmed that the *mat*-genes evolve rapidly. Furthermore, the evolutionary trajectories are related to the reproductive modes of the taxa; likelihood methods revealed that positive selection acting on specific codons drives the diversity in heterothallic taxa, while among homothallic taxa the rapid evolution is due to a lack of selective constraint. The latter finding is supported by presence of stop codons and frame shift mutations disrupting the open reading frames of *mat a-1*, *mat A-2 *and *mat A-3 *in homothallic taxa. Lower selective constraints of *mat*-genes was found among homothallic than heterothallic taxa, and comparisons with non-reproductive genes argue that this disparity is not a nonspecific, genome-wide phenomenon.

**Conclusion:**

Our data show that the *mat*-genes evolve rapidly in *Neurospora*. The rapid divergence is due to either adaptive evolution or lack of selective constraints, depending on the reproductive mode of the taxa. This is the first instance of positive selection acting on reproductive genes in the fungal kingdom, and illustrates how the evolutionary trajectory of reproductive genes can change after a switch in reproductive behaviour of an organism.

## Background

Rapidly evolving genes are those with a higher than average percentage of amino-acid substitutions between species. Rapid evolution may be due to adaptive evolution, which occurs when natural selection promotes amino-acid divergence. Alternatively, rapid evolution may be due to a lack of functional constraint; for example, a pseudogene rapidly accumulates mutations because of an absence of purifying selection.

Comparative sequencing studies among a wide range of taxonomic groups have led to the discovery that reproductive genes evolve more rapidly than other genes. In animals, essentially all steps in fertilization where the genes have been identified there is evidence of rapid divergence, and numerous reports of positively selected animal reproductive genes exist [1]. In contrast, in plants and fungi the selective forces driving reproductive gene diversity, and the functional consequences of reproductive gene evolution, are just beginning to be understood. In filamentous ascomycetes, comparatively low levels of between species nucleotide identity have been observed for mating-type genes, which constitute the master-regulators of sexual reproduction in this group of fungi [2]. Consequently these genes have been suggested to evolve at a faster rate than genes coding for e.g. metabolic enzymes [2-4], although the question has remained whether the rapid evolution is a result of stochastic or deterministic processes.

Many fungi have both asexual and sexual stages as a part of their life cycle. Their sexual mating systems are classified as either heterothallic or homothallic. In heterothallic fungi, strains must be of opposite mating type for sexual reproduction and morphogenesis, while homothallic species, generally thought to evolve from heterothallic ancestors [[5] and references therein], reproduce sexually without a mating partner. In all heterothallic filamentous ascomycetes examined to date, the *mat *locus is the sole determinant of mating type, and sexual development in heterothallic taxa is regulated by alternative *mat A *or *mat a *genes at this locus. Alternate sequences at *mat*, denoted idiomorphs [6], lack significant sequence similarity and encode different transcriptional regulators [7-10]. Genetic analyses of the model species *Neurospora crassa *have identified one open reading frame (ORF) in the *mat a *idiomorph (*mat a-1*) as the major mating regulator in *mat a *strains [8]. The *mat A *idiomorph contains three genes, one of which is the main regulator of sexual development in *mat A *strains (*mat A-1*) [7,11]. While MAT A-1 and MAT a-1 constitute the critical factors for both mating and sexual development in *N. crassa*, MAT A-2 and MAT A-3 are suggested to increase the efficiency of the process [12].

**Figure 1:**
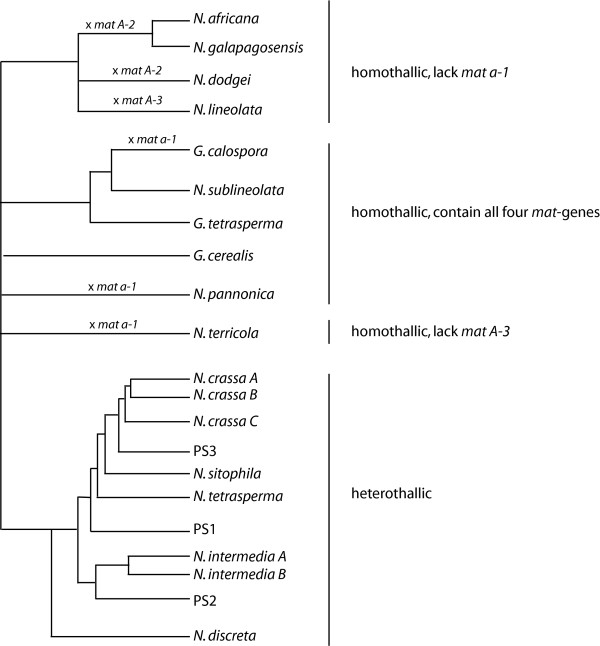
Evolutionary relationship of the included taxa. Topology is constructed from previously reported topologies based on rDNA loci (28S, ITS), nuclear gene loci (*Bml*, *ccg-7*, *mat a-1*, *mat A-1*), and four anonymous nuclear loci [36-38, 46]. A cross indicates lineages where any of the ORFs is disrupted. A, B and C indicates intraspecific subgroups and PS1 through 3 phylogenetic species [46].

In contrast to many yeast species [13], filamentous ascomycetes of either mating type are unable to switch to the opposite mating type. Nevertheless, it has been suggested that conversion from heterothallic to homothallic mode in filamentous ascomycetes resides within the *mat *locus, and that homothallic species carry both idiomorphs in a single thallus [14]. In so called pseudohomothallic species, represented in this study by *Neurospora tetrasperma*, two haploid nuclei of opposite mating-type (*mat a *and *mat A*) are maintained in each heterokaryotic ascospore progeny and vegetative cell, while in true homothallic species *mat*-genes from both idiomorphs are found within a single haploid genome. The structure of the mating type genes in homothallic *Neurospora *has not been determined in detail; however, hybridizations using cloned portions of the idiomorphs of *N. crassa *to the genome of related homothallic taxa have revealed three classes of homothallic taxa (Figure 1). The haploid genomes of one group contain both *mat a *and *mat A *sequences [15]. Another distinct group consists of taxa containing the entire *mat A *sequence, while the *mat a-1 *gene is absent [16,17]. Finally, the hybridization patterns of the homothallic *N. terricola *provide evidence for the presence of *mat a-1*, *mat A-1 *and *mat A-2 *sequences, but it appears to lack parts of the A-idiomorphic region corresponding to the *mat A-3 *region [15].

Here we present the first, to our knowledge, extensive study of molecular evolution of fungal mating-type genes in relation to reproductive strategy. We sequenced the four *mat*-genes of *Neurospora *and *Gelasinospora *taxa, which we for simplicity refer to as *Neurospora *in this study. In addition, for comparative purposes we sequenced parts of three genes with function assumed to be independent of reproductive mode of fungi; encoding actin (*act*), translational elongation factor EF-1 alpha (*tef-1*) and glyceraldehyde 3-phosphate dehydrogenase (*ccg-7*). We confirm that the *mat*-genes evolve rapidly in this group of fungi and show that the evolutionary trajectory of each of the *mat*-genes is specifically related to the reproductive modes of the taxa; positive selection drives *mat*-gene diversity in heterothallic taxa, while the rapid evolution is due to a lack of selective constraint among homothallic taxa.

## Results

### Sequence analysis of mat a-1

Amplification products and sequences of *mat a-1*, originally described by Staben and Yanofsky [8], were successfully obtained from all investigated heterothallic strains of mating type a (*mat a *strains), and from all homothallic strains except *N. africana*, *N. galapagosensis*, *N. dodgei *and *N. lineolata*, thus confirming a previous report [17], suggesting that these four homothallic taxa lack the *mat a-1 *ORF. Taken together, *mat a-1 *was sequenced from twelve heterothallic and five homothallic strains, and the alignments of nucleotide and amino acid sequences of the gene are shown in Supplemental Figure S1 (Additional file 1). Several differences were found when comparing genomic and cDNA sequences of strain FGSC #4200 with the originally published *N. crassa *sequence of the same strain [8]. The most striking difference was an additional cytosine found downstream from the conserved, DNA binding domain of the high mobility group (HMG), at nucleotide position 1074, resulting in a shift in the ORF at amino acid position 359. Reading the resulting sequence until the first stop-codon results in a 438 amino acid MAT a-1 polypeptide in *N. crassa *instead of 381, as was originally suggested by Staben and Yanofsky [8]. Consequently, the C-terminal part of the protein is different from that of the previously reported MAT a-1 protein. This feature of FGSC #4200 was consistently found in all strains analyzed, supporting our revised description of *mat a-1*.

The size of the ORF of *mat a-1 *was found to be identical in all heterothallic taxa included in this study, and only a few amino acid substitutions were found. However, this was not the case among the six homothallic taxa, especially in the C-terminal part in which insertions and deletions of amino acids were found. In three of the homothallic taxa, *N. pannonica*, *N. terricola *and *G. calospora*, sequencing of both genomic and cDNA revealed disrupted *mat a-1 *ORFs (Figure 1). First, *N. pannonica *was found to lack a 125 bp region in the third exon (nucleotide position 826 to 965, Supplemental Figure S1 (Additional file 1)) located between two stretches of cytosine nucleotides conserved in the other taxa. This deletion in *N. pannonica *results in a frame shift introducing a premature stop codon 25 amino acids downstream of the deletion. In *N. terricola*, a stop codon was found at position 308 and in *G. calospora *an inserted adenosine that results in a frame shift at amino acid position 438 was found, although the HMG-box remained intact. Taken together, these data show that only two of the homothallic taxa included in this study possess a *mat a-1 *gene that is not modified.

### Sequence analyses of mat A-1

Amplification products and sequences of *mat A-1 *were successfully obtained from the 12 heterothallic *mat A *and the nine homothallic strains investigated here. The alignments of nucleotide and amino acid sequences of *mat A-1 *are shown in Supplemental Figure S1 (Additional file 1). We confirmed the 293 amino-acid *mat A-1 *sequence of reference strain FGSC# 2489 of *N. crassa*, originally published by Glass et al. 1990 [7], including a DNA-binding α1 domain showing similarity to the α1 transcription factor of *Saccharomyces cerevisiae *[7]. All other strains included here, however, differed from strain FGSC# 2489 in that amino acids at positions 32 and 33 were found to be lacking. With the exception of this difference, and a premature stop codon found in the heterothallic taxon phylogenetic species 1 (PS1) resulting in a truncated protein lacking the last three amino acids, the length of the ORF of *mat A-1 *was identical among all heterothallic and homothallic strains investigated here.

### Sequence analysis of mat A-2

The *mat A-2 *ORF was successfully amplified and sequenced from all investigated heterothallic *mat A *strains and the homothallic strains. The nucleotide and amino acid alignments of the gene are shown in Supplemental Figure S1 (Additional file 1), and confirmed the presence of a DNA-binding, acidic amphipathic α-helix (HPG-domain) [11]. A number of differences were found when comparing the sequence of genomic DNA and cDNA from strain FGSC #2489 with the originally published sequence of the same strain [11], and these were also found in all the other strains analyzed, indicating sequencing errors in the original submission.

*N. intermedia *(both subgroups NiA and NiB) and *N. crassa *(NcC) were found to lack the start codon of *mat A-2*. No alternative start-codon was found when investigating 40 bp of the upstream sequence, but another methionine was found at aa-position 6 in both these taxa, which may serve as an alternative start-codon. This pattern was confirmed for additional strains of *N. intermedia *(NiA: FGSC# 8844) and *N. crassa *(NcC: FGSC#8865) (data not shown). With these exceptions, the size of the *mat A-2 *ORF is identical among the heterothallic taxa. However, among the homothallic taxa, the size of the *mat A-2 *ORF is not conserved. Deletions of up to 6 amino acids are found in *G. cerealis*, *G. calospora *and *N. sublineolata*, and, more importantly, in *N. dodgei, N. africana*, and *N. galapagosensis *the *mat A-2 *ORF is disrupted by a stop codon (Figure 1). In *N. dodgei*, the stop occurred at codon 39, while in *N. africana *and *N. galapagosensis*, the stop was found at codon 288. By sequencing *mat A-2 *from cDNA of *N. dodgei *and *N. galapagosensis *we confirmed the structure of the ORF in these strains. However, in spite of several independent trials, we failed to amplify *mat A-2 *from cDNA of *N. africana *(data not shown).

### Sequence analysis of mat A-3

We successfully amplified and sequenced *mat A-3 *from all investigated *mat A *and homothallic strains, except *N. terricola*, in which this gene previously has been indicated to be absent [15]. The nucleotide and amino acid alignment of *mat A-3 *is shown in Supplemental Figure S1 (Additional file 1), and shows the presence of an HMG-box. When comparing our DNA sequence obtained from genomic and cDNA of strain FGSC# 2489 of *N. crassa *with the original published *mat A-3 *sequence of this strain [11], we found only one silent nucleotide difference. This difference was found in all other strains analyzed here, supporting our revised description of *mat A-3*.

The length of the ORF was found to be intact in all taxa studied here, except for the homothallic taxa *N. africana*, *N. galapagosensis*, *N. dodgei *and *N. lineolata*. Sequencing of both genomic and cDNA from these four taxa revealed that amino acids 100–177 have been deleted in *N. africana *and *N. galapagosensis*, a region spanning the first half of the HMG-box. In *N. dodgei*, no start codon was found, although a methionine was found after 30 amino acids, which might serve as a start codon for *mat A-3 *in this taxon. Finally, *N. lineolata *has a stop-codon at position 215 (Supplemental Figure S1, Additional file 1).

### Evolutionary pattern among sites

**Figure 2:**
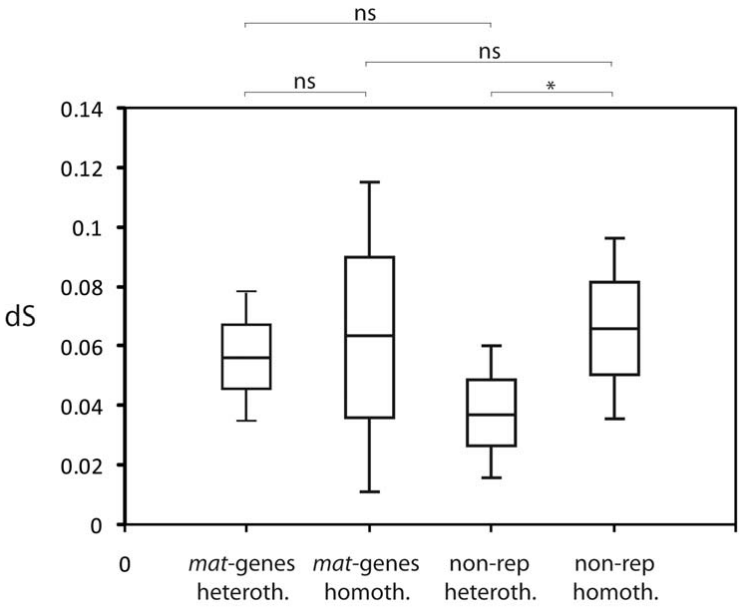
Mean rate of synonymous substitutions (d_S_) for *mat*- and non-reproductive genes of the heterothallic and homothallic datasets, obtained through pairwise comparisons between taxa in all possible combinations. Heterothallic and homothallic non-reproductive genes are statistically different from each other (*P *= 0.05, Mann-Whitney U-test).

Figure 2 shows the synonymous substitution rate (d_S_) for *mat*- and non-reproductive genes of the heterothallic and homothallic datasets, obtained through pairwise comparisons between taxa in all possible combinations. We found that d_S _did not differ significantly between *mat*- and non-reproductive genes within each reproductive class. Furthermore, we found that the synonymous substitution rate of the *mat*-genes did not differ between heterothallic and homothallic taxa, while for the non-reproductive genes we found a significantly higher d_S _in the homothallic as compared to the heterothallic dataset (*P *= 0.05, Mann-Whitney *U*-test).

**Figure 3:**
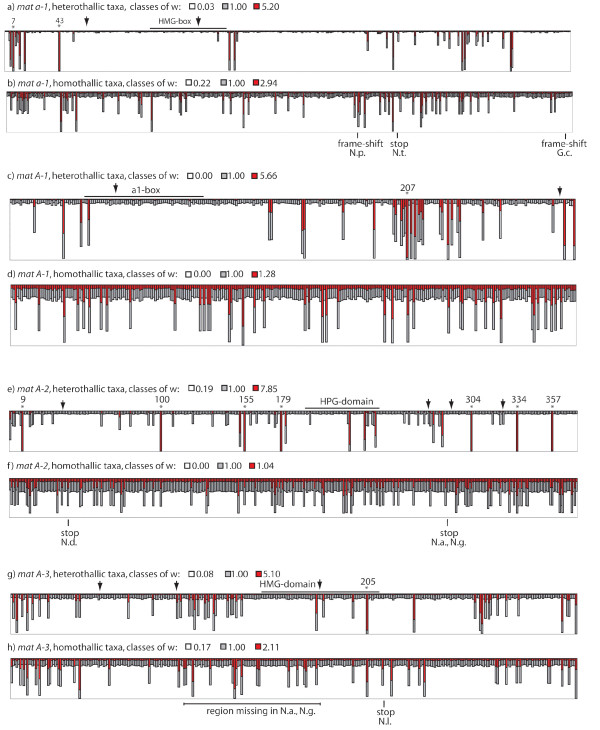
Probabilities that sites of the *mat*-genes belong to site classes with different selective pressures. Bar height represents the BEB probabilities for each site to belong to one of the three site classes obtained by running model 2a in PAML version 3.14. Asterisks indicate a probability of > 95% that the site belongs to a class with ω > 1 in the positive-selection and/or B&ω-models implemented in codeml-package, with site number as in amino acid alignment in Supplemental Figure S1. Arrow indicate intron splicing positions and pins indicate disrupted ORFs in N.a. (*N. africana*), N.g. (*N. galapagosensis*), N.t. (*N. terricola*), N.l. (*N. lineolata*), G.c. (*G. calospora*), N. p. (*N. pannonica*). Functional domains of each gene, as defined by Debuchy and Turgeon [14], are indicated.

Six models of variable ratios of nonsynonymous (d_N_) to synonymous substitution rates (d_S_), ω, among gene codons (site models outlined by Nielsen and Yang [18] and implemented in PAML version 3.14 [19]) were used to investigate the evolutionary constraints acting on each gene. Detailed descriptions of the models are given in the Methods section. Supplemental Table S1 (Additional file 1) lists likelihood ratio statistics and parameter estimates for the four *mat*-genes, and three genes which function is assumed to be independent of reproductive mode of a fungus; *act*, *tef-1 *and *ccg-7 *[20-22]. The Bayes Empirical Bayes (BEB) probabilities for each codon of the *mat*-genes to fit into one of the three classes of sites obtained by running the positive-selection model are shown in Figure 3.

We found a higher ω among *mat*-genes than among non-reproductive genes (*P *< 0.05, Fisher exact test). Given an equal synonymous substitution rate between *mat *and non-reproductive genes within each reproductive class (Figure 2), this finding indicates that *mat*-genes evolve faster than the non-reproductive genes. Furthermore, all four *mat*-genes were found to evolve faster among the homothallic than the heterothallic taxa; although the synonymous substitution rate of the *mat*-genes was equal among these groups, the averaged value of ω for each *mat*-gene, obtained using the one-ratio model implemented in PAML version 3.14, was higher in the homothallic compared to the heterothallic dataset (0.385 versus 0.144 for *mat a-1*, 0.487 versus 0.233 for *mat A-1*, 0.581 versus 0.386 for *mat A-2 *and 0.427 versus 0.316 for *mat A-3*; *P *< 0.001, Fisher exact tests). This pattern was also found for the *tef-1 *gene, for which we found a ω of 0.133 in the homothallic dataset as compared to 0.017 for the heterothallics. In contrast, the ω between homothallic and heterothallic dataset did not differ for the *act *or the *ccg-7 *genes. For the *act*-gene we estimated ω of 0 for both datasets and for *ccg-7 *we found ω of 0.102 versus 0.097 for the heterothallic and homothallic datasets, respectively.

*Mat a-1 *was found to be the most conserved *mat*-gene in both datasets, with lowest average ω ratios. Although ω was smaller in the heterothallic than the homothallic dataset, *mat a-1 *was found to evolve under positive selection among the heterothallic taxa (i.e. both models allowing for sites with ω > 1 fitted the data significantly better than the corresponding neutral model, *P *< 0.05). Bayes empirical Bayes (BEB) calculations for posterior probabilities of sites belonging to site classes revealed two codons, codon 7 and 43, with a posterior probability > 0.95 to belong to the class with ω > 1, using both the positive-selection and the beta&ω model (Figure 3a).

The sites of *mat a-1 *showing an elevated ω were not identical between the heterothallic and homothallic datasets (Figure 3a, b). The second exon was found to be extremely conserved among the heterothallic taxa, and codes for a DNA binding HMG-domain. The PRPPNAYILYRK motif suggested by Staben & Yanofsky [8] to be functionally important for mating, based on conservation between *Schizosaccharomyces pombe *and *N. crassa*, was found to be completely conserved in both heterothallic and homothallic taxa possessing this gene, but variation was found within the other part of the HMG-box for both groups of taxa (Figure 3a, b).

For *mat A-1*, the neutral models of evolution fitted the data equally well as the models of positive selection. The rapidly evolving sites in the heterothallic dataset were clustered into domains, e.g. in the C-terminal end of *mat A-1 *we found a stretch of adjacent rapidly evolving sites, including one site with a BEB posterior probability of *P *> 0.95 to belong to a class with ω > 1, while the α1-box was found to be conserved (Figure 3c). This was in contrast to the homothallic dataset in which the rapidly evolving sites are more evenly distributed along the gene (Figure 3d).

In *mat A-2 *we found the most pronounced difference between the heterothallic and homothallic dataset. In the heterothallic dataset, *mat A-2 *was found to evolve under positive selection. BEB calculations of posterior probabilities revealed seven sites with *P *> 0.95 to belong to the class with ω > 1 using the positive-selection and the beta&ω model (Figure 3e). This was very different from the homothallics in which a large proportion of sites were found to evolve neutrally; more than half of the codons were found in a class with ω = 1.011 using the discrete model. Noteworthy, the discrete model fitted the data significantly better than the one-ratio model for all datasets except the *mat A-2 *of the homothallics, for which the one-ratio model with a ω of all codons of 0.581 fitted equally well. The HPG-domain that was previously suggested to be conserved among euascomycetes [14] was found to be conserved in our heterothallic dataset, except for three rapidly evolving sites. However, the HPG-domain is not conserved in the homothallic dataset (Figure 3e, f).

In *mat A-3*, the smallest difference in average ω between the heterothallics and the homothallics was observed. Similarly to *mat a-1*, and in contrast to the observations at *mat A-1 *and *mat A-2*, when using the discrete model no codons were found in a class with ω ≈ 1 in the homothallic dataset for *mat A-3*. The HMG domain was more conserved in the heterothallic than in the homothallic taxa. However, the one site found to be in a class with *P *> 0.95 of positive selection (site 205) in the heterothallics was located within this domain.

The investigated region of the *act*-gene was found to be extremely conserved in both datasets. This was also the case for all codons of *tef-1 *for the heterothallic dataset, while a small proportion (3.3%) of the sites of this gene was found to evolve under positive selection among homothallic taxa (i.e. found in a class with ω of 6.062 using both the discrete and positive selection model: Supplemental Table S1 (Additional file 1)). For both of these genes, no neutrally evolving codons were identified in any of the datasets. For the *ccg-7 *gene the majority (> 90%) of the sequenced codons are conserved in both datasets, however, a small proportion of the codons was found in a class with ω of 1.6 in the heterothallic dataset and 1.175 in the homothallic dataset, under the discrete model (Supplemental Table S1, Additional file 1).

## Discussion

In this study we confirm that the *mat*-genes evolve rapidly in *Neurospora*, and show that the rapid divergence is due to either adaptive evolution or lack of selective constraint, depending on the reproductive mode of taxa.

A lower overall value of ω was found for the *mat*-genes among heterothallic as compared to homothallic taxa. Nevertheless, the two mating type genes *mat a-1 *and *mat A-2 *were found to evolve under positive selection in heterothallic taxa. Although a high between-taxa nucleotide divergence of reproductive genes in fungi has been observed previously [2-4], this is the first evidence of positive selection of reproductive genes in this kingdom. The finding fits directly with patterns observed for animal reproductive genes, in which essentially all identified genes are characterized by high levels of nonsynonymous divergence and positive selection [1,23,24]. None of the positively selected sites were found within the regions encoding known functional domains of the genes, and the biological significance of alterations at the positively selected sites is unknown. MAT a-1 and its orthologs in other filamentous ascomycetes are characterized by a conserved region similar to motifs found in several DNA binding proteins of the high mobility group (HMG), and has been shown to bind to DNA in *in vitro *assays [25]. *Mat a-1 *is one of the master genes regulating fertilization in *mat a *strains of *Neurospora*, as well as in other species [14]. During the initial phase of mating in heterothallic *Neurospora*, the trichogynes (female receptive hyphae) grow toward and fuse with male cells of the opposite mating type, and the directed growth is mediated by chemotropic communication between *mat-*regulated pheromones diffused from male cells and their corresponding receptors on the trichogynes. Competition between individuals could potentially drive adaptation of *mat*-genes, as indirectly involved in localization of, binding to and fusion between trichogyne and conidia of opposite mating types. An alternative hypothesis would be that the signature of positive selection observed in heterothallic *mat a-1 *and *mat A-2 *could be related to mating type associated vegetative incompatibility, rather than sexual development. Vegetative incompatibility genes are highly variable, and some have been shown to evolve in a diversifying manner [26].

Our result of *mat a-1 *in heterothallic taxa confirms previous reports suggesting that an intact HMG domain and the presence of a C-terminal tail, although non-specific, are required for mating [25,27]; at least the first part of the HMG-domain is under strong purifying selection among heterothallic taxa, while lower levels of conservation are found in the C-terminal tail (Figure 3a). The finding that a group of closely related homothallic taxa lack *mat a-1 *indicate that this gene is not essential for completion of the homothallic sexual cycle, and it is possible that this function of *mat a-1 *is redundant with another gene in the genomes of this group of taxa. The premature stop codons in *mat a-1 *in the homothallic *N. pannonica *and *N. terricola *indicate that this gene may not be required for completing the sexual cycle in a wider range of homothallic taxa. However, in these two taxa the stop codons are found distal to the HMG box, and functional assays have to be performed to verify this hypothesis.

The MAT A-1 protein is characterized by the presence of an α1 domain showing similarity to the α1 transcription factor of *Saccharomyces cerevisiae *[7].* Mat A-1 *is present in all homothallic taxa investigated here, and the only gene in which the ORF is intact in all homothallic taxa, suggesting the possibility that only the α1 transcription factor is essential to promote sexual development. This scenario would mimic the situation in *Cryptococcus neoformans*, where recent findings show sexual reproduction between isolates with the same mating type [28]. The allele from *N. africana *has been shown previously to function as a mating activator and to confer mating type-associated vegetative incompatibility in *N. crassa *[29] indicating conservation of both these functions in *mat A-1 *also in homothallic *Neurospora*. Nevertheless, we found a large proportion of evenly distributed neutrally evolving sites in the homothallic taxa, even in the α1-box that is conserved among the heterothallic taxa, indicating lower selective constraints on this gene in homothallic taxa.

*Mat A-2 *encodes a protein containing an acidic amphipathic α-helix and is unique to filamentous ascomycetes. It is suggested to not be involved in fertilization, but to function in concert with the other MAT A polypeptides to regulate the expression of genes necessary for post-fertilization events [11,12], and the positive selection at this gene among heterothallic taxa could possibly be coupled to its role in recognition of opposite mating-type nuclei during sexual development.

The MAT A-3 (and MAT a-1) HMG-domain is a DNA-binding motif found in non-histone chromosomal proteins and transcription factors. In *Neurospora*, examples of homothallic taxa lacking either *mat A-3 *or *mat a-1 *are found, and although the structure of the HMG-domain differ between the genes [14] it has been proposed that the loss of *mat a-1 *or *mat A-3 *is compensated by the remaining HMG encoding gene [30]. *N. terricola*, which is the only homothallic taxa included here that lacks *mat A-3*, has a stop codon disrupting the ORF in *mat a-1*, suggesting that this protein does not function correctly in this taxon, and we have no indications of directional selection in the *N. terricola mat a-1 *gene to change function. Furthermore, two of the four taxa lacking the *mat a-1 *gene, *N. africana *and *N. galapagosensis*, also lack the first half of the MAT A-3 HMG-domain. Thus, we are not able to provide any support for the hypothesis that the essential functions of the lost *mat A-3 *or *mat a-1 *are compensated by the remaining HMG encoding gene in *Neurospora*.

In addition to the *mat*-genes, *tef-1 *also shows a higher ω among homothallic taxa as compared to heterothallic, and this result implies that the evolution of this gene is also dependent of reproductive mode of the fungi. Interestingly, this elevated ω is due to a small proportion of positively selected sites among the homothallic taxa. At this point, we have no explanation for the observation, but it implies that *tef-1 *is involved in sexual development in *Neurospora*, or that it is affected by genetic linkage through a close physical proximity to the *mat *locus [31].

In contrast to the heterothallic taxa, rapid evolution of *mat*-genes in homothallic *Neurospora *is caused by genetic decay. Instead of being conserved, the *mat*-genes in homothallic *Neurospora *evolve under relaxed selective constraints. This is evident from a higher proportion of neutrally evolving codons among the homothallics as well as observations of a disrupted ORF in three of the four *mat*-genes in homothallic taxa. Comparisons with non-reproductive genes argue that this disparity is *mat*-specific, and not a nonspecific, genome-wide, phenomenon. The observations here provide a parallel to the observations of ecotypes with additional loss-of-function mutations at one or more SI-modifier genes after the evolutionary switch to autogamy in the *Arabidopsis thaliana *lineage [32], but is the first time low selective constraints of reproductive proteins associated with the switch to homothallism has been reported from fungi.

Our results indicate that we observe an early stage of degeneration of *mat*-genes in homothallic *Neurospora*. With the exception of *mat A-2 *in *N. africana*, we found that all genes are expressed in homothallic taxa despite frame shifts and stop codons, and the DNA sequences downstream of the disruptions are relatively conserved. This short degeneration time of the *mat*-genes in homothallic taxa might explain the results where the alterations in the *N. africana mat A-1 *are not sufficient to abolish its functions when assayed in a heterothallic relative [29]. Furthermore, the observation that they have not yet become pseudogenes, in the sense that they do not exhibit degenerative features that prevent their expression [33], indicates a low cost associated with their expression.

The results presented here indicate that once a *Neurospora *switches reproductive mode from heterothallic to homothallic, it expresses low selective pressure for maintenance of functional *mat*-genes. This finding, together with the observation that both heterothallic counterparts of the *mat*-genes are not required for sexual development in certain taxa [15,17], implies that within homothallic *Neurospora*, activation of sexual reproduction can be independent of *mat a*/*A *interaction. In other filamentous ascomycetes conversion from heterothallic to homothallic mode evidently resides within the *mat *locus [5] and both idiomorphic counterparts of heterothallic *mat*-genes are required for homothallic sexual development. In these taxa, the function of *mat*-genes has been conserved [34]. This study indicates that the transition to homothallism in *Neurospora *may represent a different paradigm. The exact nature of this difference is not known, but it would involve differences downstream of *mat a/A *interaction.

If functional mating-type genes are essential for outcrossing in the homothallics, the observed genetic decay may lead to a progressive loss of outcrossing functions and ultimately, to asexuality. However, alternative ways of outcrossing, independent of *mat*-genes may be possible, as expression of pheromones and receptors can be detected in some strains deleted for the *mat *locus [35]. Furthermore, it is possible for strains to bypass the need for fertilization by the formation of heterokaryons, which may lead to outcrossing, as done routinely in the laboratory with the homothallic *Aspergillus nidulans *[36].

## Conclusion

In this study we show that the *mat*-genes evolve rapidly in *Neurospora*, and that the rapid divergence is due to either adaptive evolution or lack of selective constraint, depending on the reproductive mode of taxa. In heterothallic *Neurospora*, the *mat*-genes evolve in an adaptive manner while once a *Neurospora *switches reproductive mode from heterothallic to homothallic there is a low selective advantage for maintenance of functional *mat*-genes. To our knowledge, this is the first example showing that the evolutionary trajectory of reproductive proteins changes after a switch in reproductive behaviour of an organism.

## Methods

### Fungal material

Thirty-four strains belonging to 21 taxa of *Neurospora *and *Gelasinospora *were used in this study (Table 1). Recent studies show that these two genera are closely related and not reciprocally monophyletic [37-39]. Therefore we include homothallic taxa of both genera in this study, and for simplicity refer to all of them as *Neurospora*. The strains were obtained from the Fungal Genetics Stock Center (University of Kansas Medical Center, Kansas City, KS).

**Table 1:** Fungal strains used in this study

Taxa	FGSC number	Mating system	Mating type	*mat*-gene analyzed^4^
Heterothallic taxa:				
*N. crassa *Shear & Dodge	2489	heteroth.	A	-
*N. crassa *Shear & Dodge	4200	heteroth.	a	-
*N. crassa *Shear & Dodge (NcA^1^)	8900	heteroth.	A	A1, A2, A3
*N. crassa *Shear & Dodge (NcA^1^)	8848	heteroth.	a	a1
*N. crassa *Shear & Dodge (NcB^1^)	8830	heteroth.	A	A1, A2, A3
*N. crassa *Shear & Dodge (NcB^1^)	8772	heteroth.	a	a1
*N. crassa *Shear & Dodge (NcC^1^)	8858	heteroth.	A	A1, A2, A3
*N. crassa *Shear & Dodge (NcC^1^)	8860	heteroth.	a	a1
PS3^2^	8838	heteroth.	A	A1, A2, A3
PS3^2^	8835	heteroth.	a	a1
*N. sitophila *Shear & Dodge	8770	heteroth.	A	A1, A2, A3
*N. sitophila *Shear & Dodge	412	heteroth.	a	a1
*N. tetrasperma *Shear & Dodge	8774	pseudohomoth.^3^	A	A1, A2, A3
*N. tetrasperma *Shear & Dodge	8775	pseudohomoth.^3^	a	a1
PS1^2^	8817	heteroth.	A	A1, A2, A3
PS1^2^	8815	heteroth.	a	a1
*N. intermedia *Tai (NiA^1^)	8901	heteroth.	A	A1, A2, A3
*N. intermedia *Tai (NiA^1^)	8841	heteroth.	a	a1
*N. intermedia *Tai (NiB^1^)	8844	heteroth.	A	A1, A2, A3
*N. intermedia *Tai (NiB^1^)	8768	heteroth.	a	a1
PS2^2^	8847	heteroth.	A	A1, A2, A3
PS2^2^	8853	heteroth.	a	a1
*N. discreta *Perkins & Raju	8780	heteroth.	A	A1, A2, A3
*N. discreta *Perkins & Raju	8827	heteroth.	a	a1
Homothallic taxa containing all *mat*-genes:				
*G. calospora *(Mouton) Moreau & Moreau	958	homoth.	-	a1, A1, A2, A3
*G. cerealis *Dowding	959	homoth.	-	a1, A1, A2, A3
*N. pannonica *Krug & Khan	7221	homoth.	-	a1, A1, A2, A3
*N. sublineolata *(Furuya & Udagawa) von Arx	5508	homoth.	-	a1, A1, A2, A3
Homothallic taxon lacking *mat A-3*:				
*N. terricola *Gochenaur & Backus	1889	homoth.	-	a1, A1, A2
Homothallic taxa lacking *mat a-1*:				
*N. africana *Huang & Backus	1740	homoth.	-	A1, A2, A3
*N. galapagosensis *Mahoney & Backus	1739	homoth.	-	A1, A2, A3
*N. dodgei *Nelson & Novak	1692	homoth.	-	A1, A2, A3
*N. lineolata *Frederick & Uecker	1910	homoth.	-	A1, A2, A3

^1^Intraspecific subgroups and ^2 ^phylogenetic species designated by Dettman *et al*. 2003 [47]

^3^Analyzed as heterothallic since it has been shown to occasionally outcross in nature [49].

^4^ORF included in evolutionary analyses.

### Nucleic acid extraction and complementary DNA (cDNA) construction

Strains were grown in minimal medium broth [40] with 1% sucrose for 3 days at 37°C, after which nucleic acids were extracted as described previously [41]. Total RNA was treated with DNase I according to the manufacturer's protocol (Fermentas, Burlington, Canada). cDNA was synthesized from total RNA by reverse transcription using an oligo(dT) primer (5'-T_25_VN-3') and Moloney Murine Leukemia Virus (M-MLV) reverse transcriptase (Invitrogen), according to the manufacturers instructions.

### Selection of loci

The entire coding regions of the four *mat*-genes, and partial coding regions of three additional genes; encoding actin (*act*: 767 bp) [22], translational elongation factor EF-1 alpha (*tef-1*: 834 bp) [20] and glyceraldehyde 3-phosphate dehydrogenase (*ccg-7*: 753 bp) [21], were investigated in this study. *Act*, *tef-1 *and *ccg-7 *were included for comparative purposes, and selected to represent genes which function is independent of reproductive mode of a fungus. As both physical proximity and expression levels can have an effect on evolutionary rate [42,43], these genes were selected to be located on different chromosomes, and expression levels of *tef-1 *and *ccg-7 *have been shown to vary between different treatments [20,21].

### PCR amplification

All included loci were amplified from one selected strain per taxa. Published sequences for *mat a*, *mat A*, *act*, *tef-1 *and *ccg-7 *of *N. crassa *were used as templates for each gene when designing primers. For isolates where amplification of *mat A-3 *was not possible using primers based on the *N. crassa *gene sequence, a primer walking sequencing strategy was used for searching the *N. crassa *genome sequence [31] for conserved region upstream from *mat A-3*. All primer sequences and GenBank accession numbers of reference sequences are given as Supplemental Table S2 (Additional file 1). PCR was performed with 1.55 U ThermoWhite *Taq *DNA polymerase (Saveen & Werner, Limhamn, Sweden) on a GeneAmp PCR System 9700 (Applied Biosystems, Foster City, CA) according to the manufacturer's instructions.

### DNA sequencing

The PCR products were purified using a PCR purification kit (QIAquick, Qiagen) and sequencing was performed by Macrogen Inc., Seoul, Korea, utilizing ABI 3730 XL automated sequencers (Applied Biosystems, Foster City, CA). Raw sequence data was analyzed using the Sequencher 4.1.4 software (Gene Codes Corporation, Ann Arbor, MI) and BioEdit version 7.0.5.2 [44].

### Codon-based likelihood analyses

Likelihood-based tests were used to investigate the nature of evolutionary pressure acting on the *mat-*genes in *Neurospora*, using the CODEML program of the PAML version 3.14 package [19,45]. All analyses were carried out using separate datasets consisting of the heterothallic or homothallic taxa, as well as a dataset containing both hetero- and homothallics. For each model, equilibrium codon frequencies were estimated from the average nucleotide frequencies at each codon position, amino acid distances were assumed to be equal, and the transition/transversion ratio (κ) was estimated from the data. For all other parameters, we use the default settings provided by Yang et al. [46]. We assumed linkage between collinear sites. The tree structure file used as input file in PAML was: (((G_calospora, N_sublineolata), G_tetrasperma),((N_africana, N_galapagosensis), N_dodgei, N_lineolata), G_cerealis, N_terricola, N_pannonica,((((((((N_crassaA, N_crassaB), N_crassaC), PS3), N_sitophila), N_tetrasperma), PS1),((N_intermediaA, N_intermedia B), PS2)), N_discreta)); shown in Figure 1 and constructed based on the topology obtained from previous reports where neighbor-joining, maximum parsimony and bayesian methods were used to construct species-phylogenies based on multiple loci [37-39,47]. We assume the true evolutionary history of the genes under study is the same as the history of the species. When only a subset of the taxa was used in the analyses, the corresponding subset of the topology was used. Sequences downstream of stop codons and frameshift mutations were omitted from the analyses. We verified that including the sequence downstream of the disruption did not affect the result by performing parallel runs, in which stop codons and frameshift mutations were omitted form the sequences.

We estimated the synonymous substitution rate (d_S_) for each gene by comparing rates between taxa in all possible combinations (runmode = -2 in PAML 3.14 [19]). A Mann-Whitney U-test (Statistica 7.1, StatSoft, Tulsa, OK) was utilized to examine whether the synonymous substitution pattern differs between *mat*- and non-reproductive genes in heterothallic and homothallic taxa.

Models of variable ratios of nonsynonymous (d_N_) to synonymous substitution rates (d_S_), ω, among sites were used to test for the presence of sites under different evolutionary constraints, and to identify them. We used six models outlined by Nielsen and Yang [18] and implemented in PAML version 3.14 [19]. The one-ratio model (Nssites 0) assumes one ω for all sites, while the discrete model (Nssites 3) uses a general discrete distribution with three site classes estimated from the data. The nearly-neutral model (Nssites 1a) assumes two classes of sites in the protein, with ω < 1 and ω = 1. The positive-selection model (Nssites 2a) adds a third class of sites to the neutral model, in which ω > 1. The beta model (Nssites 7) uses a β distribution of ω over sites: β (p, q), which, depending on the parameters p and q, can take various shapes in the interval (0,1). The beta&ω model (Nssites 8) adds an extra class of sites to the beta model, with a proportion of ω estimated from the data.

To verify which of the models best fits the data, likelihood ratio tests (LRTs) were performed by comparing twice the difference in log-likelihood values (-2lnΔ) between two models using a *χ*^2 ^distribution, with the number of degrees of freedom equal to the differences in the number of parameters between the models. For each dataset, we made three comparisons: first, the one-ratio model (Nssites 0) was compared to the discrete model (Nssites 3), to test for a variation of ω among codons within the gene. To test whether a gene evolve under positive selection, the two models including a class of codons with positively selected sites (i.e. ω > 1; models Nssites 2a and Nssites 8) were compared to their corresponding neutral models (Nssites 1a and Nssites 7, respectively) using 4, 2 and 2 degrees of freedom, respectively [45].

We identified particular sites in the genes that were likely to have evolved under positive selection by using the Bayes empirical Bayes (BEB) calculation of posterior probabilities for site classes implemented in the positive-selection- and beta&ω models [48].

## Authors' contributions

Lotta Wik carried out the majority of the molecular work, participated in sequence alignment and helped to draft the manuscript. Magnus Karlsson participated in the in the design of the study, the molecular work, the statistical analyses and helped to draft the manuscript. Hanna Johannesson initiated the study, performed the statistical analyses and drafted the manuscript. All authors read and approved the final manuscript.

## Supplementary Material

Additional File 1 Supplemental Table S1. Likelihood ratio statistics and parameter estimates for the two datasets as inferred under six models of ω over codons. Supplemental Table S2. Primers used in the current study. Supplemental Figure S1. Nucleotide and amino acid alignments of all genes and strains investigated in this study, and the originally published sequence of the *mat*-genes (Genbank accession numbers M54787, M33876). For the *mat*-genes, the entire coding sequence is aligned, while for the non-reproductive genes the alignment includes the part of the coding region amplified with primers given in the Supplemental Table S2. Click here for file
