# Supplementary material for: The evolutionary trajectory of the mating-type (mat) genes in Neurospora relates to reproductive behavior of taxa
Source: BMC Evol Biol. 2008 Apr 11;8:109. doi: 10.1186/1471-2148-8-109 (PMC2335104; doi:10.1186/1471-2148-8-109)
Supplement: Additional File 1 — Supplemental Table S1. Likelihood ratio statistics and parameter estimates for the two datasets as inferred under six models of ω over codons. Supplemental Table S2. Primers used in the current study. Supplemental Figure S1. Nucleotide and amino acid alignments of all genes and strains investigated in this study, and the originally published sequence of the mat-genes (Genbank accession numbers M54787, M33876). For the mat-genes, the entire coding sequence is aligned, while for the non-reproductive genes the alignment includes the part of the coding region amplified with primers given in the Supplemental Table S2. [file 1471-2148-8-109-S1.pdf]

Supplemental Figure. Nucleotide alignment of the coding region of *mat a-1*

```
                                 10        20        30        40        50        60
                        ....|....|....|....|....|....|....|....|....|....|....|....|
4200-N.crassaA          ATGGACGGTAACTCGACACACCCCGCTCCAAACCTCAAGACTACTATGGCTTGGTCGCGC
8848-N.crassaA          ............................................................
8772-N.crassaB          ............................................................
8860-N.crassaC          .................G..........................................
8835-PS3                ............................................................
412-N.sitophila         ........C........C.T.............G...........T..............
8775-N.tetrasperma      ........C........C.C.............G...........T..............
8815-PS1                ........C........C.C....C........G...........T..............
8841-N.intermediaA      .................G..........................................
8768-N.intermediaB      .................G..........................................
8853-PS2                ............................................................
8827-N.discreta         ........CG..CT...G...........G.C.G.......C...T..............
958-G.calospora         ........C.G..T...G...........GG......C.T.C..CT..A...........
5508-N.sublineolata     ........C.G..T...G...........GG..G...C.T.C..CT..A...........
959-G.cerealis          ........C....T...G......C....G...G.......C..CT..............
7221-N.pannonica        ........C....T...G...........GG..G.......C.TCT..............
1889-N.terricola        ........C....T...G...........GG..G.......C..CT..........T...
M54787-N.crassaA        ............................................................
```

```
                                 70        80        90       100       110       120
                        ....|....|....|....|....|....|....|....|....|....|....|....|
4200-N.crassaA          ATCTCAAACCAACTCGGTCACTGGAATGACCGCAAGGTCATTGCCATTCCTCTGAGCGAC
8848-N.crassaA          ..A.........................................................
8772-N.crassaB          ............................................................
8860-N.crassaC          ............................................................
8835-PS3                ............................................................
412-N.sitophila         ............................................................
8775-N.tetrasperma      ............................................................
8815-PS1                ............................................................
8841-N.intermediaA      ............................................................
8768-N.intermediaB      ............................................................
8853-PS2                ............................................................
8827-N.discreta         .....G....................C........................T.......T
958-G.calospora         .....G...........A........C.................................
5508-N.sublineolata     .....G...........A........C.................................
959-G.cerealis          .....G....................CA................................
7221-N.pannonica        .....C...........A........C.................................
1889-N.terricola        .....C...........A........C.................................
M54787-N.crassaA        ..A.........................................................
```

Supplemental Figure. Nucleotide alignment of the coding region of *mat a-1*

```
                                                          Intron 1
                           130       140       150       160       170       180
                   ....|....|....|....|....|....|....|....|....|....|....|....|
4200-N.crassaA     TTCCTTAACACCCACCCTGACATTCAGTCTGGCATCATCGCCGAGTTCAAGAAAGCGACT
8848-N.crassaA     ............................................................
8772-N.crassaB     ..T.........................................................
8860-N.crassaC     ............................................................
8835-PS3           ............................................................
412-N.sitophila    ......TG....................................................
8775-N.tetrasperma ......TG....................................................
8815-PS1           ......TG.....................C..............................
8841-N.intermediaA ............................................................
8768-N.intermediaB ............................................................
8853-PS2           ............................................................
8827-N.discreta    ......C................................A....................
958-G.calospora    .....C..G...G......T..C..........C........AGC...............
5508-N.sublineolata .....C..G...G......T..C..........C........AGC...............
959-G.cerealis     ......T.....................................................
7221-N.pannonica   ......C.....G......T...C..................AGC...............
1889-N.terricola   ......C.....G......T......................AGC...............
M54787-N.crassaA   ............................................................
```

```
                           190       200       210       220       230       240
                   ....|....|....|....|....|....|....|....|....|....|....|....|
4200-N.crassaA     GGCGAAGAGGGCATGTTTGCCCGCGATCCTGAATCATTGGGAATCATGCTTCTTGGTCCC
8848-N.crassaA     ................................G...........................
8772-N.crassaB     ............................................................
8860-N.crassaC     ............................................................
8835-PS3           ............................................................
412-N.sitophila    ..T.................T..............G........T...............
8775-N.tetrasperma ..T.................T..............G........T...............
8815-PS1           ..T.................T.......................................
8841-N.intermediaA ............................................................
8768-N.intermediaB ............................................................
8853-PS2           ............................................................
8827-N.discreta    ..T..............C..T........C..G...C...................C...
958-G.calospora    ..T.................T...........G...C..........C........C...
5508-N.sublineolata ..T.................T...........G...C..........C........C...
959-G.cerealis     ..T.................T........C..G...C...................C...
7221-N.pannonica   ..T.................T........C..G...C...................C...
1889-N.terricola   ..T.................T........C..G...C...................C...
M54787-N.crassaA   ............................................................
```

Supplemental Figure. Nucleotide alignment of the coding region of *mat a-1*

```
                                      250       260       270       280       290       300
                                 ....|....|....|....|....|....|....|....|....|....|....|....|
4200-N.crassaA                   GTCAAGCTGTTCAAGCCCGACAGTGTCGTCGTCGACGGCAACCTGTTCTGGGATCCCAAG
8848-N.crassaA                   ............................................................
8772-N.crassaB                   ............................................................
8860-N.crassaC                   ............................................................
8835-PS3                         ............................................................
412-N.sitophila                  ............................................................
8775-N.tetrasperma               ............................................................
8815-PS1                         ............................................................
8841-N.intermediaA               ............................................................
8768-N.intermediaB               ............................................................
8853-PS2                         ............................................................
8827-N.discreta                  ....................T...................................T...
958-G.calospora                  .C..................TG..........T...........T..............A
5508-N.sublineolata              .C..................TG..........T...........T...............
959-G.cerealis                   .C..................T................................C......
7221-N.pannonica                 .C..................T.......................T...............
1889-N.terricola                 .C..........................................T...............
M54787-N.crassaA                 ............................................................

                                      310       320       330       340       350       360
                                 ....|....|....|....|....|....|....|....|....|....|....|....|
4200-N.crassaA                   GGCATCCATGCTTCGGCACCCAAGGAGCAGCAGAAGAAGGCCAAGATCCCTCGCCCTCCC
8848-N.crassaA                   ............................................................
8772-N.crassaB                   ............................................................
8860-N.crassaC                   ...............................................T............
8835-PS3                         ............................................................
412-N.sitophila                  ............................................................
8775-N.tetrasperma               ............................................................
8815-PS1                         ............................................................
8841-N.intermediaA               ...............................................T........C...
8768-N.intermediaB               ...............................................T........C...
8853-PS2                         ............................................................
8827-N.discreta                  ............................................................
958-G.calospora                  ...............A.......................AT......T............
5508-N.sublineolata              ........................................T......T............
959-G.cerealis                   .........A..................................................
7221-N.pannonica                 ..............T.........................T......T........C...
1889-N.terricola                 ...............................................T........C...
M54787-N.crassaA                 ............................................................
```

Supplemental Figure. Nucleotide alignment of the coding region of *mat a-1*

```
                             370       380       390       400       410       420
                     ....|....|....|....|....|....|....|....|....|....|....|....|
4200-N.crassaA       AATGCCTACATCTTGTACCGTAAGGACCATCATCGTGAGATCCGCGAGCAGAATCCCGGA
8848-N.crassaA       ............................................................
8772-N.crassaB       ............................................................
8860-N.crassaC       ............C...............................................
8835-PS3             ............C............................T..................
412-N.sitophila      ........................................................T...
8775-N.tetrasperma   ............................................................
8815-PS1             ............................................................
8841-N.intermediaA   .............................C..............................
8768-N.intermediaB   ............................................................
8853-PS2             ............................................................
8827-N.discreta      .................T.................C.................C......
958-G.calospora      ..C.................A........................A...G...C......
5508-N.sublineolata  ..C.................A..A.....................A...G...C......
959-G.cerealis       ..C.................C................................C......
7221-N.pannonica     ..C.................A............................G...C......
1889-N.terricola     ..C.................A...A........................G...C......
M54787-N.crassaA     ............................................................
```

Intron 2

```
                                           Intron 2
                                              |
                             430       440    v  450       460       470       480
                     ....|....|....|....|....|....|....|....|....|....|....|....|
4200-N.crassaA       CTTCACAATAACGAGATTTCGGTCATCGTCGGCAACATGTGGCGTGATGAGCAGCCGCAC
8848-N.crassaA       ............................................................
8772-N.crassaB       ............................................................
8860-N.crassaC       ..........................T.................................
8835-PS3             ............................................................
412-N.sitophila      ............................................................
8775-N.tetrasperma   ............................................................
8815-PS1             ............................................................
8841-N.intermediaA   ..........................T.................................
8768-N.intermediaB   ..........................T.................................
8853-PS2             ............................................................
8827-N.discreta      ........C...................................................
958-G.calospora      ..C.....C...................................................
5508-N.sublineolata  ..C.....C...................................................
959-G.cerealis       T.C.....C.......................T...........................
7221-N.pannonica     ..C.....C...................................................
1889-N.terricola     ..C.....C...................................................
M54787-N.crassaA     .................---........................................
```

Supplemental Figure. Nucleotide alignment of the coding region of *mat a-1*

```
                             490       500       510       520       530       540
                     ....|....|....|....|....|....|....|....|....|....|....|....|
4200-N.crassaA       ATTCGCGAGAAATATTTCAACATGTCCAATGAGATCAAGACCAGACTGTTGCTGGAGAAT
8848-N.crassaA       ............................................................
8772-N.crassaB       ............................................................
8860-N.crassaC       ............................................................
8835-PS3             ............................................................
412-N.sitophila      .............................................A..........AC..
8775-N.tetrasperma   .............................................A..........AC..
8815-PS1             .............................................A..........AC..
8841-N.intermediaA   ..................C.........................................
8768-N.intermediaB   ............................................................
8853-PS2             ............................................................
8827-N.discreta      .................T...........................A....AT.....C..
958-G.calospora      ........................G...C............................C..
5508-N.sublineolata  ........................G...C............................C..
959-G.cerealis       ........................G....................A...........C..
7221-N.pannonica     ........................G....................A...........C..
1889-N.terricola     ........................G................T...A...........C..
M54787-N.crassaA     ............................................................

                             550       560       570       580       590       600
                     ....|....|....|....|....|....|....|....|....|....|....|....|
4200-N.crassaA       CCCGACTATCGCTACAATCCGCGTCGGTCTCAAGACATTCGCAGGCGCGTCTCGCCGTAT
8848-N.crassaA       ............................................................
8772-N.crassaB       ............................................................
8860-N.crassaC       ............................................................
8835-PS3             ............................................................
412-N.sitophila      ............................................................
8775-N.tetrasperma   ............................................................
8815-PS1             .....G......................................................
8841-N.intermediaA   ............................................................
8768-N.intermediaB   ............................................................
8853-PS2             ............................................................
8827-N.discreta      ....................A................................A......
958-G.calospora      ...........T...GC...A......C................A..T............
5508-N.sublineolata  ...........T...GC...A......C................A...............
959-G.cerealis       ....................A..C....................................
7221-N.pannonica     ....................A.......................................
1889-N.terricola     ....................A.......................................
M54787-N.crassaA     ............................................................
```

Supplemental Figure. Nucleotide alignment of the coding region of *mat a-1*

```
                              610       620       630       640       650       660
                     ....|....|....|....|....|....|....|....|....|....|....|....|
4200-N.crassaA       CTCAAGATCAAGCTCCTCAACTACGACGTTAATGGCAACCTTCTTTGGGGCACCGTCAAC
8848-N.crassaA       ............................................................
8772-N.crassaB       ............................................................
8860-N.crassaC       ............................................................
8835-PS3             ............................................................
412-N.sitophila      .......................T..........................A.........
8775-N.tetrasperma   .......................T..........................A.........
8815-PS1             .......................T..............T...........A.........
8841-N.intermediaA   ............................................................
8768-N.intermediaB   ............................................................
8853-PS2             ............................................................
8827-N.discreta      .................T.....T.............................T......
958-G.calospora      .......................T....C.C.............................
5508-N.sublineolata  .......................T....C.C.............................
959-G.cerealis       .....A.................T....C...............................
7221-N.pannonica     .....A.................T....C...............................
1889-N.terricola     .....A.................T....C...............................
M54787-N.crassaA     ............................................................

                              670       680       690       700       710       720
                     ....|....|....|....|....|....|....|....|....|....|....|....|
4200-N.crassaA       GCCGAGGATGCTGCGCTGATTCGGACTCACTTCCATGGAGTCGTTCGTGTTGAGGAAATG
8848-N.crassaA       ............................................................
8772-N.crassaB       ............................................................
8860-N.crassaC       ............................................................
8835-PS3             ............................................................
412-N.sitophila      ............................................................
8775-N.tetrasperma   ............................................................
8815-PS1             ..........................C.................................
8841-N.intermediaA   ............................................................
8768-N.intermediaB   ............................................................
8853-PS2             ............................................................
8827-N.discreta      ..............C..A..........................................
958-G.calospora      .G...............A..........................................
5508-N.sublineolata  .G...............A..........................................
959-G.cerealis       .................A..................A.......................
7221-N.pannonica     .................A..........................................
1889-N.terricola     .................A..........................................
M54787-N.crassaA     ............................................................
```

Supplemental Figure. Nucleotide alignment of the coding region of *mat a-1*

```
                                730       740       750       760       770       780
                       ....|....|....|....|....|....|....|....|....|....|....|....|
4200-N.crassaA         GATGATGGTTGCAGAATCGTCTGCCGTCCCGTCGCAGGATCTAGAAAACTTCGCGCCGCC
8848-N.crassaA         ............................................................
8772-N.crassaB         ............................................................
8860-N.crassaC         ........C...................................................
8835-PS3               ............................................................
412-N.sitophila        ........C...................................................
8775-N.tetrasperma     ........C...................................................
8815-PS1               ........C...................................................
8841-N.intermediaA     ........C...................................................
8768-N.intermediaB     ........C...................................................
8853-PS2               ............................................................
8827-N.discreta        ........C...........T....................C..................
958-G.calospora        ..C.....C..T.....T..........................G...............
5508-N.sublineolata    ..C.....C..T.....T..........................G...............
959-G.cerealis         ........C........T..............G........C..................
7221-N.pannonica       ........C........T..........................................
1889-N.terricola       ........C........T..........................................
M54787-N.crassaA       ............................................................
```

```
                                790       800       810       820       830       840
                       ....|....|....|....|....|....|....|....|....|....|....|....|
4200-N.crassaA         GTTGTCGACACTTGGATGCCTCGCTACACGGTTGACACAACCCCCGTCACCGAGGACGA-
8848-N.crassaA         ...........................................................-
8772-N.crassaB         ...........................................................-
8860-N.crassaC         ...........................................................-
8835-PS3               ...........................................................-
412-N.sitophila        ...........................................................-
8775-N.tetrasperma     ...........................................................-
8815-PS1               .........................................T.....T...........-
8841-N.intermediaA     ...........................................................-
8768-N.intermediaB     ...........................................................-
8853-PS2               ...........................................................-
8827-N.discreta        ........T...........G.....T................................-
958-G.calospora        ...........A..............T........TC........CC.G.T........-
5508-N.sublineolata    ...........A..............T........TC........CC.G.T........-
959-G.cerealis         ..........................T........TG...........G.T........-
7221-N.pannonica       ..........................T........TG........---------------
1889-N.terricola       ..........................T........TG.............T........-
M54787-N.crassaA       ...........................................................-
```

Supplemental Figure. Nucleotide alignment of the coding region of *mat a-1*

```
                            850       860       870       880       890       900
                    ....|....|....|....|....|....|....|....|....|....|....|....|
4200-N.crassaA      -----CGATGCACCGGCTTTCAACTTCAATGATCCC---------TTGGGCGGTGCTTAT
8848-N.crassaA      -----........A......................---------...............
8772-N.crassaB      -----...............................---------...............
8860-N.crassaC      -----..............................T---------C............T.
8835-PS3            -----...............................---------...............
412-N.sitophila     -----...........................C...---------...............
8775-N.tetrasperma  -----...........................C...---------...............
8815-PS1            -----...........................C...---------...............
8841-N.intermediaA  -----...............................---------...............
8768-N.intermediaB  -----...............................---------...............
8853-PS2            -----...............................---------...............
8827-N.discreta     -----.........T..............C..C...---------...............
958-G.calospora     --TGC..CG..C.A...............C..C...ATGGACCCC....C...C...G..
5508-N.sublineolata --TGC..CG..C.A...............C..C...ATGGACCCC....C...C...G..
959-G.cerealis      --------C.A..A..................C...---------........C......
7221-N.pannonica    ------------------------------------------------------------
1889-N.terricola    -----........A...............C..C...ATAGACCCC....C...C......
M54787-N.crassaA    -----........A......................---------...............

                            910       920       930       940       950       960
                    ....|....|....|....|....|....|....|....|....|....|....|....|
4200-N.crassaA      TTCCCTTTGAATGAGCACCTCTGGATCACTGTCAACCAAAACCCTCCCTTCAATGCCCCT
8848-N.crassaA      ............................................................
8772-N.crassaB      ............................................................
8860-N.crassaC      ..........................T.................................
8835-PS3            ............................................................
412-N.sitophila     ..............T................................A............
8775-N.tetrasperma  ..............T................................A............
8815-PS1            ..............T..............C.................A............
8841-N.intermediaA  ..........................T.................................
8768-N.intermediaB  ..........................T.....T...........................
8853-PS2            ............................................................
8827-N.discreta     .....AC.......T.........G....C...........T..A...............
958-G.calospora     .....CG.......T.........GC...C...--------------.............
5508-N.sublineolata ......G.......T.........GC...C...--------------.......A.....
959-G.cerealis      ......C.......T.........G....C..........C...................
7221-N.pannonica    ------------------------------------------------------------
1889-N.terricola    ......A.C.....T.......A.G....C..........C...................
M54787-N.crassaA    ............................................................
```

Supplemental Figure. Nucleotide alignment of the coding region of *mat a-1*

```
                                   970       980       990      1000      1010      1020
                          ....|....|....|....|....|....|....|....|....|....|....|....|
4200-N.crassaA            CCCCCCAATCCCAACCCACACCTGGATTTCGTTCACCCCGACGGCATGGAGGCAGTTGTT
8848-N.crassaA            ............................................................
8772-N.crassaB            ............................................................
8860-N.crassaC            ............................................................
8835-PS3                  ............................................................
412-N.sitophila           ............................................................
8775-N.tetrasperma        ............................................................
8815-PS1                  ............................................................
8841-N.intermediaA        ....T.......................................................
8768-N.intermediaB        ............................................................
8853-PS2                  ............................................................
8827-N.discreta           .....T...........T.....A................................CA..
958-G.calospora           ............G..G.......C.G.............A.........C..........
5508-N.sublineolata       ............G..G.........G.............A.........C..........
959-G.cerealis            .........................................................A..
7221-N.pannonica          ----.....................................................A..
1889-N.terricola          ..................................................C......A..
M54787-N.crassaA          ............................................................

                                  1030      1040      1050      1060      1070      1080
                          ....|....|....|....|....|....|....|....|....|....|....|....|
4200-N.crassaA            CACAACGTTCAGAACATGATCGCTCAGGTCCAGGAGGCTAACGAGGCTGCTGCCGCTAAC
8848-N.crassaA            ............................................................
8772-N.crassaB            ............................................................
8860-N.crassaC            ............................................A...............
8835-PS3                  ............................................................
412-N.sitophila           .......................................................T....
8775-N.tetrasperma        .......................................................T....
8815-PS1                  .......................................................T....
8841-N.intermediaA        ............................................................
8768-N.intermediaB        ............................................................
8853-PS2                  ............................................................
8827-N.discreta           ...........AG.............T.................A.T.............
958-G.calospora           ............G.......T.................C..TC.................
5508-N.sublineolata       ............G.......T..C..............C..TC.................
959-G.cerealis            .......................C..............C..T......A..A........
7221-N.pannonica          ............G..........C..............C..T..................
1889-N.terricola          ............G..........C..............C..T..................
M54787-N.crassaA          .....................................................-......
```

Supplemental Figure. Nucleotide alignment of the coding region of *mat a-1*

```
                            1090      1100      1110      1120      1130      1140
                    ....|....|....|....|....|....|....|....|....|....|....|....|
4200-N.crassaA      GCTACCACCGCCACCACCGCTGCGTCTGCTGTCACTCAGGTTATGGCTGATGATACCATT
8848-N.crassaA      ............................................................
8772-N.crassaB      ..........A...........................................G.....
8860-N.crassaC      ............................................................
8835-PS3            ............................................................
412-N.sitophila     .G................................................C.........
8775-N.tetrasperma  .G................................................C.........
8815-PS1            ..................................................C.........
8841-N.intermediaA  ............................................................
8768-N.intermediaB  ............................................................
8853-PS2            ............................................................
8827-N.discreta     ..C...T....T...............A..A.............A.....C........C
958-G.calospora     ..C...T...TT........G...C...TC....................CA.C.....C
5508-N.sublineolata ..C...T...TT........G...C...TC....................CA.C.....C
959-G.cerealis      ..C...T....T............C....C....................C........C
7221-N.pannonica    ..C...T....T............C....C.............C......CA.......C
1889-N.terricola    ..C...T....T............C....C....................C........C
M54787-N.crassaA    ............................................................
```

```
                            1150      1160      1170      1180      1190      1200
                    ....|....|....|....|....|....|....|....|....|....|....|....|
4200-N.crassaA      AACCCAGCTCTCATTCCCACTGTGAACACTCATGCATCTGTTCTTCCCTACGTCCATACC
8848-N.crassaA      ............................................................
8772-N.crassaB      ............................................................
8860-N.crassaC      ................................................C...........
8835-PS3            ............................................................
412-N.sitophila     ................................................C...........
8775-N.tetrasperma  ................................................C...........
8815-PS1            ................................................C..A........
8841-N.intermediaA  ................................................C...........
8768-N.intermediaB  ................................................C...........
8853-PS2            ............................................................
8827-N.discreta     ........G....................CA....G...........TC..A........
958-G.calospora     ........C.....C...............A....GA.C........TC..A........
5508-N.sublineolata ........C.....C...............A....GA.C........TC..A........
959-G.cerealis      ........G.....................A....GA.C........TC...........
7221-N.pannonica    ........G.....................A....GA.C........TC...........
1889-N.terricola    ........G.....................A....GA.C........TC...........
M54787-N.crassaA    ............................................................
```

Supplemental Figure. Nucleotide alignment of the coding region of *mat a-1*

```
                            1210      1220      1230      1240      1250      1260
                    ....|....|....|....|....|....|....|....|....|....|....|....|
4200-N.crassaA      ATTCCTGACAACGCCACCGTTACGCCTTCCGCTACTGGAAACTCGGTTCACGTTGTTACA
8848-N.crassaA      ............................................................
8772-N.crassaB      ............................................................
8860-N.crassaC      ............................................................
8835-PS3            ............................................................
412-N.sitophila     ............................................................
8775-N.tetrasperma  ............................................................
8815-PS1            ............................................................
8841-N.intermediaA  ............................................................
8768-N.intermediaB  ............................................................
8853-PS2            ............................................................
8827-N.discreta     ...........T.................T..............................
958-G.calospora     .....C..............C...........C........T..................
5508-N.sublineolata .....C..............C...........C........T..................
959-G.cerealis      ................................C....................G......
7221-N.pannonica    ................................C........T..................
1889-N.terricola    ................................C........T........T.........
M54787-N.crassaA    ............................................................

                            1270      1280      1290      1300      1310      1320
                    ....|....|....|....|....|....|....|....|....|....|....|....|
4200-N.crassaA      CCCGGTCACCAAGGATATCACCTCATCCACTGGCCCCCGCTGTCCAGTGAGAA-CATGGA
8848-N.crassaA      .....................................................-......
8772-N.crassaB      .....................................................-......
8860-N.crassaC      .....................................................-......
8835-PS3            .....................................................-......
412-N.sitophila     .....................................................-......
8775-N.tetrasperma  .....................................................-......
8815-PS1            .....................................................-......
8841-N.intermediaA  .....................................................-T.....
8768-N.intermediaB  .....................................................-......
8853-PS2            .....................................................-......
8827-N.discreta     .....C...............................................-......
958-G.calospora     ........T..G..G.....G..T...A.......T..............A..A......
5508-N.sublineolata ........T..G..G.....G..T...A.......T..............A..-......
959-G.cerealis      ..............G...................................A..-T.....
7221-N.pannonica    ...........G..............T..................GAG..A..-......
1889-N.terricola    ...........G..G..............................GAG..A..-......
M54787-N.crassaA    .....................................................-......
```

Supplemental Figure. Nucleotide alignment of the coding region of *mat a-1*

```
                         1330
                    ....|....|....|...
4200-N.crassaA      TACCGAGGACTGA-----
8848-N.crassaA      .............-----
8772-N.crassaB      .............-----
8860-N.crassaC      .............-----
8835-PS3            .............-----
412-N.sitophila     .............-----
8775-N.tetrasperma  .............-----
8815-PS1            .............-----
8841-N.intermediaA  .............-----
8768-N.intermediaB  .............-----
8853-PS2            .............-----
8827-N.discreta     .............-----
958-G.calospora     .............GGTAG
5508-N.sublineolata .............-----
959-G.cerealis      .............-----
7221-N.pannonica    .............-----
1889-N.terricola    .............-----
M54787-N.crassaA    .............-----
```

Supplemental Figure. Amino acid alignment of *mat a-1*

```
                                                                    Intron 1
                           7  10        20        30        40 43     50    |   60
                           *                                    *           v
                    ....|....|....|....|....|....|....|....|....|....|....|....|
4200-N.crassaA      MDGNSTHPAPNLKTTMAWSRISNQLGHWNDRKVIAIPLSDFLNTHPDIQSGIIAEFKKAT
8848-N.crassaA      ............................................................
8772-N.crassaB      ............................................................
8860-N.crassaC      ............................................................
8835-PS3            ............................................................
412-N.sitophila     ......L....V...L..........................C.................
8775-N.tetrasperma  ......P....V...L..........................C.................
8815-PS1            ......P.P..V...L..........................C.................
8841-N.intermediaA  ............................................................
8768-N.intermediaB  ............................................................
8853-PS2            ............................................................
8827-N.discreta     ...DL.....TV...L..........................H..........T......
958-G.calospora     ...SL.....D.TS.LT.........................K.D.VT...L..S.....
5508-N.sublineolata ...SL.....DVTS.LT.........................K.D.VT...L..S.....
959-G.cerealis      ....L...P..V...L.............N............Y.................
7221-N.pannonica    ....L.....DV..IL..........................H.D.V.......S.....
1889-N.terricola    ....L.....DV...L..........................H.D.V.......S.....
M54787-N.crassaA    ............................................................
```

```
                                                                        ______
                             70        80        90       100       110       120
                    ....|....|....|....|....|....|....|....|....|....|....|....|
4200-N.crassaA      GEEGMFARDPESLGIMLLGPVKLFKPDSVVVDGNLFWDPKGIHASAPKEQQKKAKIPRPP
8848-N.crassaA      ............................................................
8772-N.crassaB      ............................................................
8860-N.crassaC      ............................................................
8835-PS3            ............................................................
412-N.sitophila     ............................................................
8775-N.tetrasperma  ............................................................
8815-PS1            ............................................................
8841-N.intermediaA  ............................................................
8768-N.intermediaB  ............................................................
8853-PS2            ............................................................
8827-N.discreta     ............................................................
958-G.calospora     ...............I....A......G.................T.......I......
5508-N.sublineolata ...............I....A......G.........................V......
959-G.cerealis      ....................A......................T................
7221-N.pannonica    ....................A................................V......
1889-N.terricola    ....................A.......................................
M54787-N.crassaA    ............................................................
```

Supplemental Figure. Amino acid alignment of *mat a-1*

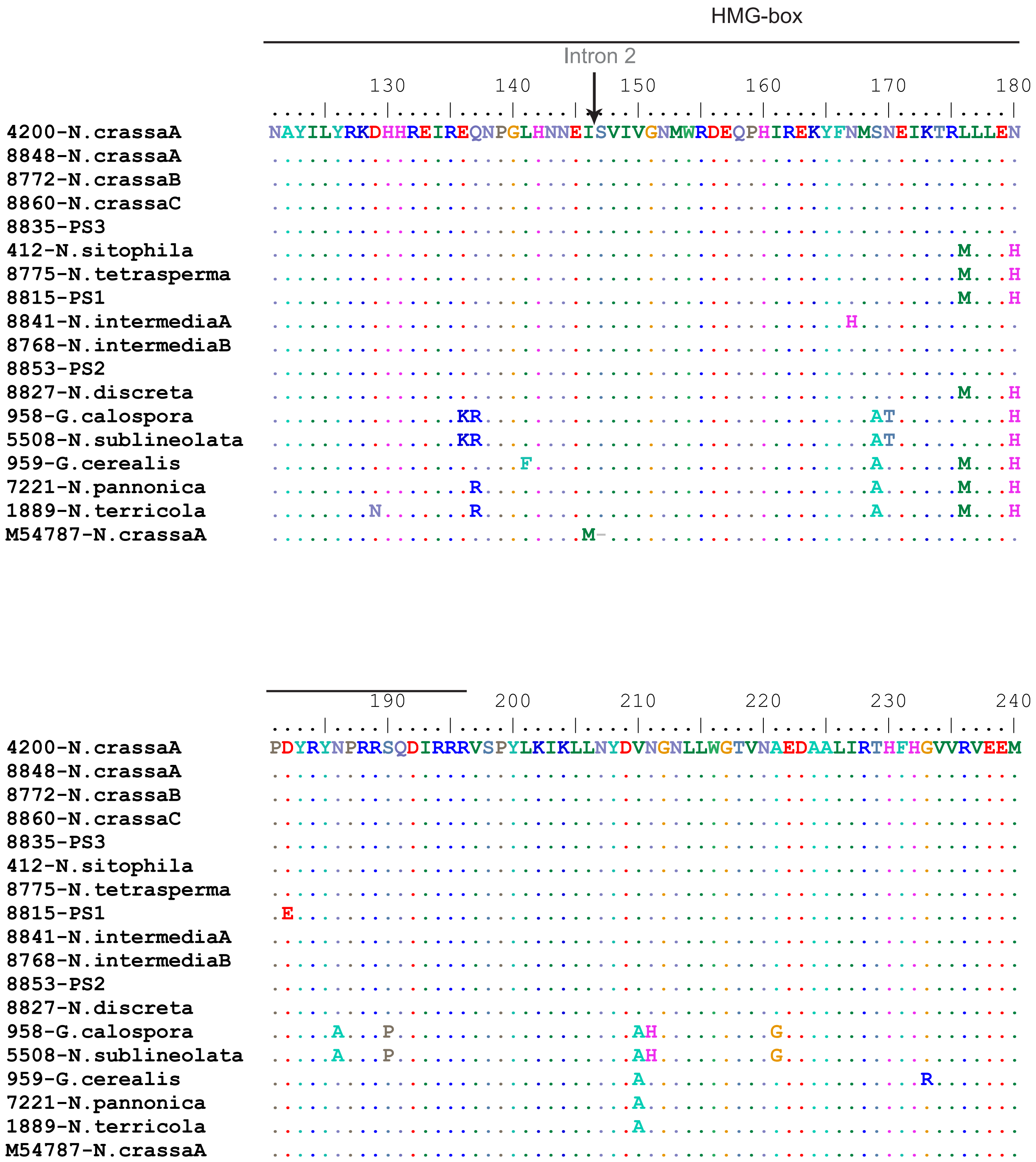

Supplemental Figure. Amino acid alignment of *mat a-1*

```
                                250       260       270       280       290       300
                       ....|....|....|....|....|....|....|....|....|....|....|....|
4200-N.crassaA         DDGCRIVCRPVAGSRKLRAAVVDTWMPRYTVDTTPVTEDD--DAPAFNFN---DPLGGAY
8848-N.crassaA         ........................................--..Q.....---.......
8772-N.crassaB         ........................................--........---.......
8860-N.crassaC         ........................................--........---......F
8835-PS3               ........................................--........---.......
412-N.sitophila        ........................................--........---.......
8775-N.tetrasperma     ........................................--........---.......
8815-PS1               ........................................--........---.......
8841-N.intermediaA     ........................................--........---.......
8768-N.intermediaB     ........................................--........---.......
8853-PS2               ........................................--........---.......
8827-N.discreta        ........................................--........---.......
958-G.calospora        ................................P..PA...-AA.Q.....DPM...A..D
5508-N.sublineolata    ................................P..PA...-AA.Q.....DPM...A..D
959-G.cerealis         ................................A...A...---EQ.....---.......
7221-N.pannonica       ................................A..-------------------------
1889-N.terricola       ................................A.......--..Q.....DPI...A...
M54787-N.crassaA       ........................................--..Q.....---.......
```

```
                                310       320       330       340       350       360
                       ....|....|....|....|....|....|....|....|....|....|....|....|
4200-N.crassaA         FPLNEHLWITVNQNPPFNAPPPNPNPHLDFVHPDGMEAVVHNVQNMIAQVQEANEAAAAN
8848-N.crassaA         ............................................................
8772-N.crassaB         ............................................................
8860-N.crassaC         ............................................................
8835-PS3               ............................................................
412-N.sitophila        ....D.....................................................V.
8775-N.tetrasperma     ....D.....................................................V.
8815-PS1               ....D.....................................................V.
8841-N.intermediaA     .....................L......................................
8768-N.intermediaB     ............................................................
8853-PS2               ............................................................
8827-N.discreta        ....D...V..............................I....D...H......V....
958-G.calospora        ..V.D...A..-----........DA..G....N..A.......D.........Q.....
5508-N.sublineolata    ..V.D...A..-----..T.....DA..G....N..A.......D.........Q.....
959-G.cerealis         ....D...V....T.........................I................TT..
7221-N.pannonica       ---------------------.IPTHTWISFT.TAWRQLFTTFRT*
1889-N.terricola       ..I.D..*
M54787-N.crassaA       ..........................................................LT
```

Supplemental Figure. Amino acid alignment of *mat a-1*

```
                                   370       380       390       400       410       420
                         ....|....|....|....|....|....|....|....|....|....|....|....|
4200-N.crassaA           ATTATTAASAVTQVMADDTINPALIPTVNTHASVLPYVHTIPDNATVTPSATGNSVHVVT
8848-N.crassaA           ............................................................
8772-N.crassaB           ...D..............A.........................................
8860-N.crassaC           ....................................H.......................
8835-PS3                 ............................................................
412-N.sitophila          G...................................H.......................
8775-N.tetrasperma       G...................................H.......................
8815-PS1                 ....................................HI......................
8841-N.intermediaA       ....................................H.......................
8768-N.intermediaB       ....................................H.......................
8853-PS2                 ............................................................
8827-N.discreta          ..S......TI...I...............N.....HI......................
958-G.calospora          ..SV....PV.......N............N.T...HI......................
5508-N.sublineolata      ..SV....PV.......N............N.T...HI......................
959-G.cerealis           ..S.....P.....................N.T...H.......................
7221-N.pannonica
1889-N.terricola
M54787-N.crassaA         LPPPPPLRLLSLRLWLMIPLTQLSF.L*


                                   430       440
                         ....|....|....|....|....|.
4200-N.crassaA           PGHQGYHLIHWPPLSSENMDTED*
8848-N.crassaA           .......................*
8772-N.crassaB           .......................*
8860-N.crassaC           .......................*
8835-PS3                 .......................*
412-N.sitophila          .......................*
8775-N.tetrasperma       .......................*
8815-PS1                 .......................*
8841-N.intermediaA       .......................*
8768-N.intermediaB       .......................*
8853-PS2                 .......................*
8827-N.discreta          .......................*
958-G.calospora          ......Q..N.......KHGYRGLR*
5508-N.sublineolata      ......Q..N.............*
959-G.cerealis           .......................*
7221-N.pannonica
1889-N.terricola
M54787-N.crassaA
```

Supplemental Figure. Nucleotide alignment of the coding region of *mat A-1*

```
                                  10        20        30        40        50        60
                         ....|....|....|....|....|....|....|....|....|....|....|....|
2489-N.crassaA           ATGTCGGGTGTCGATCAAATCGTCAAGACGTTCGCCGACCTCGCTGAGGACGACCGTGAA
8900-N.crassaA           ............................................................
8830-N.crassaB           ............................................................
8858-N.crassaC           ............................................................
8838-PS3                 .......................T....................................
8770-N.sitophila         ............................................................
8774-N.tetrasperma       ............................................................
8817-PS1                 ............................................................
8901-N.intermediaA       .........................................T..................
8844-N.intermediaB       ............................................................
8847-PS2                 ....................................A...................C...
8780-N.discreta          ........C.....C.....T...................................C...
958-G.calospora          ........C................................T.......G...T......
5508-N.sublineolata      ........C................................................G...T......
959-G.cerialis           ........C................................................G...T......
7221-N.pannonica         ........C................................................G...T......
1889-N.terricola         ........C................................................G...T......
1740-N.africana          ......T.C.................................A......GT..T......
1739-N.galapagosensis    .....AT.C.................................A......GT..T......
1692-N.dodgei            ......T.C.................................A......GT..T......
1910-N.lineolata         ......T.C................................TA......GT..T......
M33876-N.crassaA         ............................................................

                                  70        80        90       100       110       120
                         ....|....|....|....|....|....|....|....|....|....|....|....|
2489-N.crassaA           GCGGCAATGAGAGCTTTCTCAAGGATGATGCGTAGAGGTACCGAACCTGTTCGCCGAATC
8900-N.crassaA           ....................G.A...........------....................
8830-N.crassaB           ....................G.A...........------....................
8858-N.crassaC           ......................C...........------....................
8838-PS3                 ....................G.A...........------....................
8770-N.sitophila         ......................C...........------....................
8774-N.tetrasperma       ......................C...........------....................
8817-PS1                 ......................C...........------....................
8901-N.intermediaA       ......................C...........------....................
8844-N.intermediaB       ......................C...........------....................
8847-PS2                 ......................C...........------....................
8780-N.discreta          ......................C...........------...............A....
958-G.calospora          ......................T.........C.------...............A..C.
5508-N.sublineolata      ......................T.........C.------...............A..C.
959-G.cerialis           ......................C.........C.------.........C.....A..C.
7221-N.pannonica         ......................C.........C.------...............A..C.
1889-N.terricola         ......................C.........C.------...............A..C.
1740-N.africana          ......................T.........C.------...............A..C.
1739-N.galapagosensis    ......................T.........C.------...............A..C.
1692-N.dodgei            ......................T.........C.------...............A..C.
1910-N.lineolata         ......................T.........C.------....G..........A..C.
M33876-N.crassaA         ............................................................
```

Supplemental Figure. Nucleotide alignment of the coding region of *mat A-1*

```
                                                                     Intron 1
                                 130       140       150       160   |     170       180
                         ....|....|....|....|....|....|....|....|....|....|....|....|
2489-N.crassaA           CCCGCGGCAAAGAAGAAGGTCAACGGCTTCATGGGTTTCAGATCGTACTATTCCCCGCTC
8900-N.crassaA           ............................................................
8830-N.crassaB           ............................................................
8858-N.crassaC           ............................................................
8838-PS3                 ............................................................
8770-N.sitophila         ............................................................
8774-N.tetrasperma       ............................................................
8817-PS1                 ............................................................
8901-N.intermediaA       ............................................................
8844-N.intermediaB       ............................................................
8847-PS2                 ............................................................
8780-N.discreta          ......A.....................................................
958-G.calospora          .......................T..T.................................
5508-N.sublineolata      .......................T....................................
959-G.cerialis           ............................................................
7221-N.pannonica         .......T....................................................
1889-N.terricola         ........................C...................................
1740-N.africana          .................................A..........................
1739-N.galapagosensis    .................................A..........................
1692-N.dodgei            ........G........................A..........................
1910-N.lineolata         .................................A..........................
M33876-N.crassaA         ............................................................
```

```
                                 190       200       210       220       230       240
                         ....|....|....|....|....|....|....|....|....|....|....|....|
2489-N.crassaA           TTCTCTCAGCTCCCGCAAAAGGAGAGATCGCCCTTCATGACTATTCTCTGGCAGCATGAT
8900-N.crassaA           .........................................C..C...............
8830-N.crassaB           .........................................C..C...............
8858-N.crassaC           .........................................C..................
8838-PS3                 .........................................C..C...............
8770-N.sitophila         .........................................C..................
8774-N.tetrasperma       .........................................C..................
8817-PS1                 .........................................C..................
8901-N.intermediaA       .........................................C..................
8844-N.intermediaB       .........................................C..................
8847-PS2                 .........................................C..................
8780-N.discreta          .........................................C..............C...
958-G.calospora          .........................................C..............C...
5508-N.sublineolata      .........................................C..............C...
959-G.cerialis           ..........C..............................C..............C...
7221-N.pannonica         .........................................C..............C...
1889-N.terricola         ................................G........C..............C...
1740-N.africana          .................G...........A...........C..............C...
1739-N.galapagosensis    .................G...........A...........C..............C...
1692-N.dodgei            .................G...........A...........C..............C...
1910-N.lineolata         .................G...........A...........C..............C...
M33876-N.crassaA         ............................................................
```

Supplemental Figure. Nucleotide alignment of the coding region of *mat A-1*

```
                                        250       260       270       280       290       300
                                ....|....|....|....|....|....|....|....|....|....|....|....|
2489-N.crassaA                  CCCTTCCACAATGAGTGGGATTTCATGTGCTCGGTGTATTCGTCAATCCGGACCTACCTT
8900-N.crassaA                  ............................................................
8830-N.crassaB                  ............................................................
8858-N.crassaC                  ...................................A........................
8838-PS3                        ...........C................................................
8770-N.sitophila                ............................................................
8774-N.tetrasperma              ............................................................
8817-PS1                        ............................................................
8901-N.intermediaA              ............................................................
8844-N.intermediaB              .....T......................................................
8847-PS2                        ............................................................
8780-N.discreta                 ...........C..A...................................T.....T...
958-G.calospora                 ...........C..A...................................C......T..
5508-N.sublineolata             ..T........C..A...................................C......T..
959-G.cerialis                  ...........C..A...................................C.........
7221-N.pannonica                ...........C..A...................................C.........
1889-N.terricola                ...........C..A...................................C.........
1740-N.africana                 ...........C..A...A.........................G.....C.........
1739-N.galapagosensis           ...........C..A...A.........................G.....C.........
1692-N.dodgei                   ...........C..A...A.........................G.....C.........
1910-N.lineolata                ...........C..A...A.........................G.....C.........
M33876-N.crassaA                ............................................................
```

```
                                        310       320       330       340       350       360
                                ....|....|....|....|....|....|....|....|....|....|....|....|
2489-N.crassaA                  GAGCAGGAGAAGGTTACTCTGCAACTCTGGATTCACTATGCTGTCGGCCATCTGGGAGTG
8900-N.crassaA                  ............................................................
8830-N.crassaB                  ............................................................
8858-N.crassaC                  ............................................................
8838-PS3                        ............................................................
8770-N.sitophila                ............................................................
8774-N.tetrasperma              ............................................................
8817-PS1                        ............................................................
8901-N.intermediaA              ............................................................
8844-N.intermediaB              ............................................................
8847-PS2                        ............................................................
8780-N.discreta                 ..........................T.................................
958-G.calospora                 ...........T.....C........................C.................
5508-N.sublineolata             ...........T.....C........................A.................
959-G.cerialis                  .................C.....G....................................
7221-N.pannonica                ...G....................................................G...
1889-N.terricola                .................C..........................................
1740-N.africana                 ...........A.....C...........................C..............
1739-N.galapagosensis           ...........A.....C...........................C..............
1692-N.dodgei                   ...........A.....C...........................C..............
1910-N.lineolata                ...........AA....C...........................C..............
M33876-N.crassaA                ............................................................
```

Supplemental Figure. Nucleotide alignment of the coding region of *mat A-1*

```
                                 370       380       390       400       410       420
                         ....|....|....|....|....|....|....|....|....|....|....|....|
2489-N.crassaA           ATTATCCGCGACAACTACATGGCATCCTTTGGCTGGAACCTCGTCCGTTTTCCCAACGGC
8900-N.crassaA           ............................................................
8830-N.crassaB           ............................................................
8858-N.crassaC           ..C........T..............G.................................
8838-PS3                 ............................................................
8770-N.sitophila         ............................................................
8774-N.tetrasperma       ..........................G.................................
8817-PS1                 ............................................................
8901-N.intermediaA       ...........T..............G.................................
8844-N.intermediaB       ...........T..............G.....T...........................
8847-PS2                 ............................................................
8780-N.discreta          ..........................G...................A.C.G.........
958-G.calospora          ...CG................A....G...................AGC.G.........
5508-N.sublineolata      ...CG................A....G...................AGC.G.........
959-G.cerialis           ....C.....................G...................AAC.G.........
7221-N.pannonica         ....C.....................G...................AGC.G.........
1889-N.terricola         ....C.....................G...................AGC.G.........
1740-N.africana          ..........................G...................AGC.G.........
1739-N.galapagosensis    ..........................G...................AGC.G.........
1692-N.dodgei            ..........................G...........A.......AGC.G.........
1910-N.lineolata         ..........................G...................AGC.G.........
M33876-N.crassaA         ............................................................
```

```
                                 430       440       450       460       470       480
                         ....|....|....|....|....|....|....|....|....|....|....|....|
2489-N.crassaA           ACTCACGACCTCGAGCGCACGGCTCTTCCTTTGGTTCAGCACAATCTCCAGCCCATGAAC
8900-N.crassaA           ............................................................
8830-N.crassaB           ............................................................
8858-N.crassaC           ........................................C...................
8838-PS3                 ............................................................
8770-N.sitophila         ............................................................
8774-N.tetrasperma       ............................................................
8817-PS1                 ...................................C........................
8901-N.intermediaA       ........................................CT..................
8844-N.intermediaB       ........................................C...................
8847-PS2                 ............................................................
8780-N.discreta          ..G.................C........C.....CAG.........T............
958-G.calospora          ....................T.................A..G..CA..............
5508-N.sublineolata      ....................T.................A..G..C...............
959-G.cerialis           ....................C........C...........T..C...............
7221-N.pannonica         ....................C....................T..C...............
1889-N.terricola         ....................C....................T..C..T............
1740-N.africana          ....................C....................T..C...............
1739-N.galapagosensis    ....................C....................T..C...............
1692-N.dodgei            ....................C....................T..C...............
1910-N.lineolata         ....................C....................T..C...............
M33876-N.crassaA         ............................................................
```

Supplemental Figure. Nucleotide alignment of the coding region of *mat A-1*

```
                              490       500       510       520       530       540
                         ....|....|....|....|....|....|....|....|....|....|....|....|
2489-N.crassaA           GGCTTATGCCTGCTCACCAAGTGCCTCGAGAGCGGATTGCCTCTTGCCAATCCTCACTCT
8900-N.crassaA           ........................................A...................
8830-N.crassaB           ............................................................
8858-N.crassaC           ............................................................
8838-PS3                 ............................................................
8770-N.sitophila         ............................................................
8774-N.tetrasperma       ............................................................
8817-PS1                 ............................................................
8901-N.intermediaA       ............................................................
8844-N.intermediaB       ............................................................
8847-PS2                 ............................................................
8780-N.discreta          ...C.............T..........................................
958-G.calospora          ..TC.G.C....T....T.............A.........................C..
5508-N.sublineolata      ...C.G.C....T....T.............A.........................C..
959-G.cerialis           ...C..C..................................................C..
7221-N.pannonica         ...C.............T.......................................C..
1889-N.terricola         ...C.............T.......................................C..
1740-N.africana          ...C........T............................................C..
1739-N.galapagosensis    ...C........T............................................C..
1692-N.dodgei            ...C.....................................................C..
1910-N.lineolata         ...C.....................................................C..
M33876-N.crassaA         ............................................................
```

```
                              550       560       570       580       590       600
                         ....|....|....|....|....|....|....|....|....|....|....|....|
2489-N.crassaA           GTCATCGCCAAGCTTTCAGATCCTAGCTACGACATGATCTGGTTCAACAAGCGTCCTCAC
8900-N.crassaA           ............................................................
8830-N.crassaB           ............................................................
8858-N.crassaC           ............................T...............................
8838-PS3                 ............................................................
8770-N.sitophila         ............................................................
8774-N.tetrasperma       ............................................................
8817-PS1                 ............................................................
8901-N.intermediaA       ............................T...............................
8844-N.intermediaB       ............................TT..............................
8847-PS2                 ............................................................
8780-N.discreta          .........................................................T..
958-G.calospora          ..........................................A.G...............
5508-N.sublineolata      ..........................................A.G.........TT....
959-G.cerialis           ................................T...........................
7221-N.pannonica         .....T......................................................
1889-N.terricola         ............................................................
1740-N.africana          ............................................................
1739-N.galapagosensis    ............................................................
1692-N.dodgei            ............................................................
1910-N.lineolata         ............................................................
M33876-N.crassaA         ............................................................
```

Supplemental Figure. Nucleotide alignment of the coding region of *mat A-1*

```
                                610       620       630       640       650       660
                         ....|....|....|....|....|....|....|....|....|....|....|....|
2489-N.crassaA           CGTCAGCAGGGACACGCCGTTCAAACTGATGAATCTGAAGTTGGAGTTTCGGCGATGTTC
8900-N.crassaA           .........................T..................................
8830-N.crassaB           .........................T..................................
8858-N.crassaC           ...................G........................................
8838-PS3                 .........................T..................................
8770-N.sitophila         ...............................G............................
8774-N.tetrasperma       ...................G........................................
8817-PS1                 ......................................T.....................
8901-N.intermediaA       ...................G........................................
8844-N.intermediaB       ...................G........................................
8847-PS2                 ............................................................
8780-N.discreta          A........A....A.T..GC........C..T......C.C.A...G............
958-G.calospora          ...................GC........CA.T......C.......G......C.C...
5508-N.sublineolata      ...................GC........CA.T......C.......G......C.C...
959-G.cerialis           ..................AAC........CA.T......C.......G......C.C...
7221-N.pannonica         ...................GC........CA.T......C.......G......C.C...
1889-N.terricola         ...................GC........CA.T......C.......G......C.C...
1740-N.africana          ...................GC......T.CA.T......C.......G......C.C...
1739-N.galapagosensis    ...................GC......T.CA.T......C.......G......C.C...
1692-N.dodgei            ...................GC......T.CA.T......C.......G......C.C...
1910-N.lineolata         ...................GC......T.CA.T......C.......G......C.C...
M33876-N.crassaA         ............................................................
```

```
                                670       680       690       700       710       720
                         ....|....|....|....|....|....|....|....|....|....|....|....|
2489-N.crassaA           CCTCGCAATCACACGGTCGCTGCAGAGGTAGATGGCATCATCAATCTTCCTCTCTCCCAT
8900-N.crassaA           ............................................................
8830-N.crassaB           ............................................................
8858-N.crassaC           ............................................................
8838-PS3                 ............................................................
8770-N.sitophila         ............................................................
8774-N.tetrasperma       ......................T.....................................
8817-PS1                 ............................................................
8901-N.intermediaA       ............................................................
8844-N.intermediaB       .....................A...............C......................
8847-PS2                 ............................................................
8780-N.discreta          ..C......T..G.A.....C.......C......T...GC...................
958-G.calospora          ............G.A.......G................GC...................
5508-N.sublineolata      ............G.A.......G................GC...C...............
959-G.cerialis           ............G.A........................GC...................
7221-N.pannonica         ............G.A........................GC...................
1889-N.terricola         ............G.A........................GC...................
1740-N.africana          ............G.A.........................C.G.C...............
1739-N.galapagosensis    ............G.A.........................C.G.C...............
1692-N.dodgei            ............G.A.........................C.G.C...............
1910-N.lineolata         ............G.A.........................C.G.C...............
M33876-N.crassaA         ............................................................
```

Supplemental Figure. Nucleotide alignment of the coding region of *mat A-1*

```
                                     730       740       750       760       770       780
                             ....|....|....|....|....|....|....|....|....|....|....|....|
2489-N.crassaA               TGGATTCAGCAGGGAGAATTCGGTACCGAGTCTGGATACTCAGCTCAGTTTGAGACCTTG
8900-N.crassaA               ............................................................
8830-N.crassaB               ........A...................................................
8858-N.crassaC               ............................................................
8838-PS3                     ............................................................
8770-N.sitophila             ............................................................
8774-N.tetrasperma           ..........................................A.................
8817-PS1                     ............................................................
8901-N.intermediaA           ............................................................
8844-N.intermediaB           ............................................................
8847-PS2                     ............................................................
8780-N.discreta              .................T........T.....C..............A........T...
958-G.calospora              .................T.....C........C....T...G..................
5508-N.sublineolata          .................C.....C........C....T...G..................
959-G.cerialis               .................T.G...C........C....T......................
7221-N.pannonica             .................T.....C........C....T......................
1889-N.terricola             .................T.....C........C....T......................
1740-N.africana              ...C.............T.....C......G.C....T....C.................
1739-N.galapagosensis        ...C.............T.....C......G.C....T....C.................
1692-N.dodgei                ...C.............T.....C......G.C....T....C.................
1910-N.lineolata             ...C.............T.....C......G.C....T....T.................
M33876-N.crassaA             ............................................................

                                     790       800       810       820       830       840
                             ....|....|....|....|....|....|....|....|....|....|....|....|
2489-N.crassaA               TTGGATTCAATTCTCGAGAATGGACACGCCTCCAGCAATGACCCTTACAACATGGCTCTG
8900-N.crassaA               ............................................................
8830-N.crassaB               ............................................................
8858-N.crassaC               ...........C................................................
8838-PS3                     ............................................................
8770-N.sitophila             ............................................................
8774-N.tetrasperma           ...........C................................................
8817-PS1                     ............................................................
8901-N.intermediaA           ...........C.............................T..................
8844-N.intermediaB           ...........C................................................
8847-PS2                     ............................................................
8780-N.discreta              ........T.....T.............................C...............
958-G.calospora              C.......G..C..T.........A.....A...A......T..C...............
5508-N.sublineolata          ........G..C..T.........A.....A...A......T..C...............
959-G.cerialis               ........G..C..T.........A...................C.......G.......
7221-N.pannonica             ........G..C..T.........A.........A......T..C...............
1889-N.terricola             ........G..C..T.........A................T..C...............
1740-N.africana              ........G..C..T.........A.......T...........C.....T.........
1739-N.galapagosensis        ........G..C..T.........A.......T...........C.....T.........
1692-N.dodgei                ........G..C..T.........A.......T........A..C.....T.........
1910-N.lineolata             ........G..C..T.........A.......T...........C.....T.........
M33876-N.crassaA             ............................................................
```

Supplemental Figure . Nucleotide alignment of the coding region of *mat A-1*

```
                                  Intron 2
                          850        860 |      870        880
                      ....|....|....|....|....|....|....|....|..
2489-N.crassaA        GCTATCGATGTTCCCATGATGGGATTTAACGGAGGAGCATAG
8900-N.crassaA        ..........................................
8830-N.crassaB        ..........................................
8858-N.crassaC        ..........................................
8838-PS3              ..........................................
8770-N.sitophila      ......................A...................
8774-N.tetrasperma    ..........................................
8817-PS1              ......................A.......T...........
8901-N.intermediaA    .........................................A
8844-N.intermediaB    ................................C.........
8847-PS2              ..........................................
8780-N.discreta       .....G....................C.........TT....
958-G.calospora       .....G....................................
5508-N.sublineolata   .....G....................................
959-G.cerialis        .....G....................................
7221-N.pannonica      .....G..........G.........................
1889-N.terricola      .....G....................................
1740-N.africana       .G...G.G..................................
1739-N.galapagosensis .G...G.G..................................
1692-N.dodgei         .G...G.G..................................
1910-N.lineolata      .G...G.G..................................
M33876-N.crassaA      ..........................................
```

Supplemental Figure. Amino acid alignment of *mat A-1*

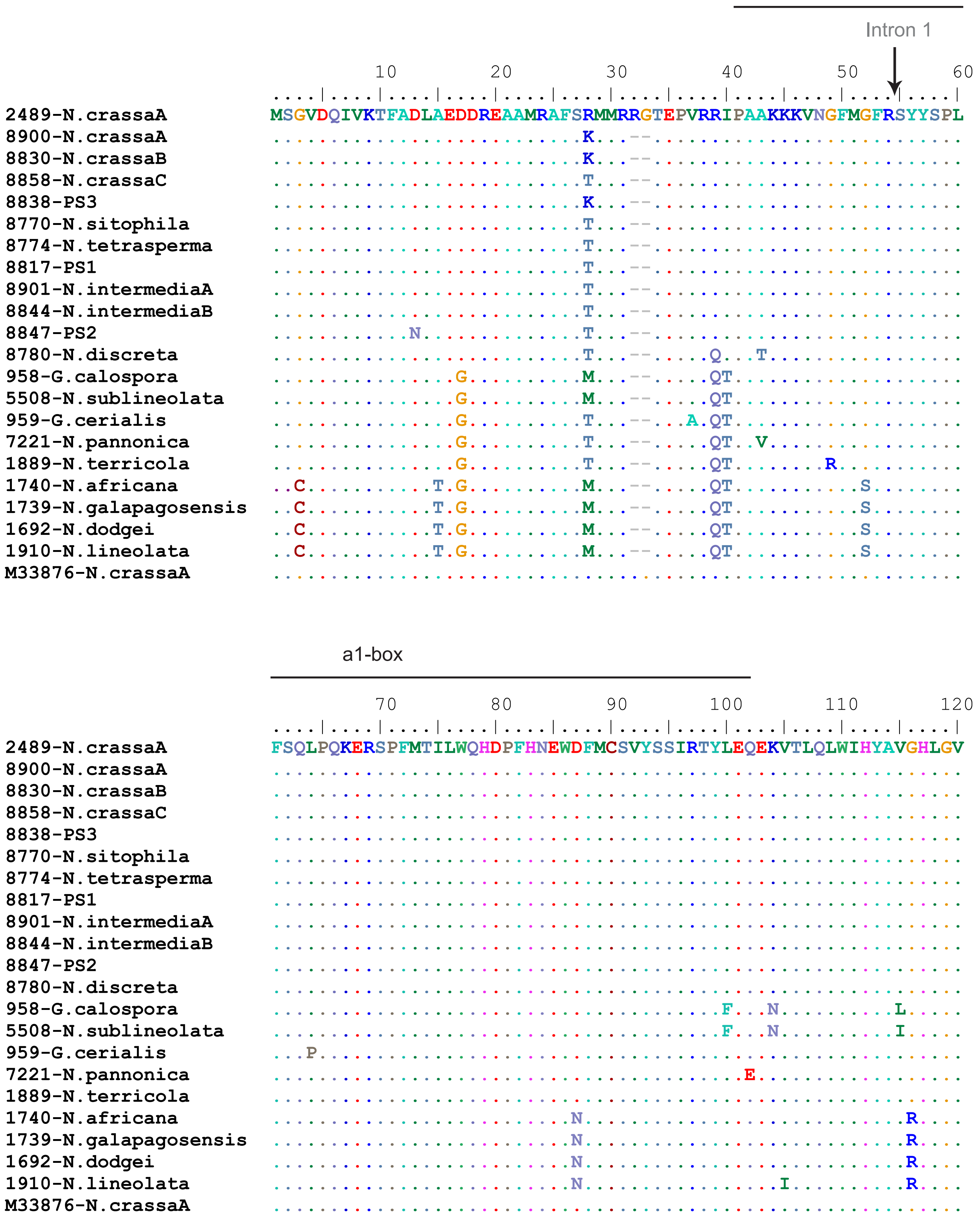

Supplemental Figure. Amino acid alignment of *mat A-1*

```
                                 130       140       150       160       170       180
                        ....|....|....|....|....|....|....|....|....|....|....|....|
2489-N.crassaA          IIRDNYMASFGWNLVRFPNGTHDLERTALPLVQHNLQPMNGLCLLTKCLESGLPLANPHS
8900-N.crassaA          .....................................................H......
8830-N.crassaB          ............................................................
8858-N.crassaC          .................................P..........................
8838-PS3                ............................................................
8770-N.sitophila        ............................................................
8774-N.tetrasperma      ............................................................
8817-PS1                ............................................................
8901-N.intermediaA      .................................P..........................
8844-N.intermediaB      .................................P..........................
8847-PS2                ............................................................
8780-N.discreta         ...............HL...............R...........................
958-G.calospora         .R.....T.......QL................Q.I......S.F.....N........P
5508-N.sublineolata     .R.....T.......QL................Q........S.F.....N........P
959-G.cerialis          .T.............QL.........................R................P
7221-N.pannonica        .T.............QL..........................................P
1889-N.terricola        .T.............QL..........................................P
1740-N.africana         ...............QL...........................F..............P
1739-N.galapagosensis   ...............QL...........................F..............P
1692-N.dodgei           ............K..QL..........................................P
1910-N.lineolata        ...............QL..........................................P
M33876-N.crassaA        ............................................................
```

```
                                                  205
                                 190       200     * 207 210       220       230       240
                                                       *
                        ....|....|....|....|....|....|....|....|....|....|....|....|
2489-N.crassaA          VIAKLSDPSYDMIWFNKRPHRQQGHAVQTDESEVGVSAMFPRNHTVAAEVDGIINLPLSH
8900-N.crassaA          ............................I...............................
8830-N.crassaB          ............................I...............................
8858-N.crassaC          .........F................G.................................
8838-PS3                ............................I...............................
8770-N.sitophila        ..............................G.............................
8774-N.tetrasperma      ..........................G....................V............
8817-PS1                ................................D...........................
8901-N.intermediaA      .........F................G.................................
8844-N.intermediaB      .........F................G....................T....T.......
8847-PS2                ............................................................
8780-N.discreta         ...................YS..RQVG...D..LE........YA....A...A......
958-G.calospora         ..............M...........G...N..L....L.....A..G.....A......
5508-N.sublineolata     ..............M...F.......G...N..L....L.....A..G.....A......
959-G.cerialis          ..........................N...N..L....L.....A........A......
7221-N.pannonica        ..........................G...N..L....L.....A........A......
1889-N.terricola        ..........................G...N..L....L.....A........A......
1740-N.africana         ..........................G..YN..L....L.....A........TD.....
1739-N.galapagosensis   ..........................G..YN..L....L.....A........TD.....
1692-N.dodgei           ..........................G..YN..L....L.....A........TD.....
1910-N.lineolata        ..........................G..YN..L....L.....A........TD.....
M33876-N.crassaA        ............................................................
```

Supplemental Figure. Amino acid alignment of *mat A-1*

```
                                                              Intron 2
                                                                  ↓
                                  250       260       270       280       290
                         ....|....|....|....|....|....|....|....|....|....|....
2489-N.crassaA           WIQQGEFGTESGYSAQFETLLDSILENGHASSNDPYNMALAIDVPMMGFNGGA*
8900-N.crassaA           .....................................................*
8830-N.crassaB           .....................................................*
8858-N.crassaC           .....................................................*
8838-PS3                 .....................................................*
8770-N.sitophila         ...............................................E.....*
8774-N.tetrasperma       ..............T......................................*
8817-PS1                 ...............................................E..*
8901-N.intermediaA       .....................................................*
8844-N.intermediaB       .....................................................*
8847-PS2                 .....................................................*
8780-N.discreta          .....D..................................M..........L*
958-G.calospora          .....D.....F...............N.TN.........M...........*
5508-N.sublineolata      .....D.....F...............N.TN.........M...........*
959-G.cerialis           .....DC....F...............N........R...M...........*
7221-N.pannonica         .....D.....F...............N..N.........M...R.......*
1889-N.terricola         .....D.....F...............N............M...........*
1740-N.africana          .L...D....A.F.P............N...........GMG..........*
1739-N.galapagosensis    .L...D....A.F.P............N...........GMG..........*
1692-N.dodgei            .L...D....A.F.P............N....E......GMG..........*
1910-N.lineolata         .L...D....A.F.S............N...........GMG..........*
M33876-N.crassaA         .....................................................*
```

Supplemental Figure. Nucleotide alignment of the coding region of *mat A-2*

```
                                  10        20        30        40        50        60
                         ....|....|....|....|....|....|....|....|....|....|....|....|
2489-N.crassaA           ATGAATCTTCTCAACATGCAACCTAAAAGGTCAGAGCAACCAGCTATGTTCGAAGAAAAC
8900-N.crassaA           ............................................................
8830-N.crassaB           ............................................................
8858-N.crassaC           ..A.......................G.........A.......................
8838-PS3                 ............................................................
8770-N.sitophila         ...........A................................................
8774-N.tetrasperma       .......A..................G.................................
8817-PS1                 ............................................................
8901-N.intermediaA       ..A.......................G.................................
8844-N.intermediaB       ..A.......................G.............T...................
8847-PS2                 ............................................................
8780-N.discreta          ...G....CA............T..G...............G.T..G...T.........
958-G.calospora          ...G....CAG..............G...A....G..G...G.T....C..A........
5508-N.sublineolata      ...G....CAG..............G...A....G..G...G.T....C..A........
959-G.cerealis           ...G....CA...............G...A...........G.T....C...........
7221-N.pannonica         ...G....CA...............G...A.........G.G.T....C...........
1889-N.terricola         ........CA...............G...A...........G.T....C...........
1740-N.africana          ...G....CA...............G...A.........T.G.T................
1739-N.galapagosensis    ...G....CA......C........G...A.........T.G.T................
1692-N.dodgei            ...G....CA...............G...A.........T.G.T................
1910-N.lineolata         ...G....CA...............G...A.........T.G.T................
M33876-N.crassaA         ............................................................
```

Intron 1

```
                                  70        80        90       100       110       120
                         ....|....|....|....|....|....|....|....|....|....|....|....|
2489-N.crassaA           CGTGCCTCTAGCCAGGAAGGCCAGGATCTCGAAGTGATGTACAAGAAACTCCATCAGCTA
8900-N.crassaA           ............................................................
8830-N.crassaB           ............................................................
8858-N.crassaC           ............................................................
8838-PS3                 ............................................................
8770-N.sitophila         ............................................................
8774-N.tetrasperma       ............................................................
8817-PS1                 ............................................................
8901-N.intermediaA       ............................................................
8844-N.intermediaB       .........................G..................................
8847-PS2                 ............................................................
8780-N.discreta          ....T.......................................................
958-G.calospora          ...A........................................................
5508-N.sublineolata      ...A.......................................................C
959-G.cerealis           ...A....................................................C...
7221-N.pannonica         ...A.......................................C................
1889-N.terricola         ...A........................................................
1740-N.africana          ...A....C.......................AG.........................G
1739-N.galapagosensis    ...A....C.......................AG.........................G
1692-N.dodgei            ...A....C.......................AG....................T....G
1910-N.lineolata         ...A....C.......................AG.........................G
M33876-N.crassaA         ............................................................
```

Supplemental Figure. Nucleotide alignment of the coding region of *mat A-2*

```
                                     130       140       150       160       170       180
                             ....|....|....|....|....|....|....|....|....|....|....|....|
2489-N.crassaA               CAGGCTAGGCTTTCCCGTTCAGTTCTTTCAGAGGCAATCAAGGAGTTCGAAGAGAACCTT
8900-N.crassaA               ............................................................
8830-N.crassaB               ............................................................
8858-N.crassaC               ............................................................
8838-PS3                     ............................................................
8770-N.sitophila             ............................................................
8774-N.tetrasperma           ............................................................
8817-PS1                     ............................................................
8901-N.intermediaA           ............................................................
8844-N.intermediaB           ............................................................
8847-PS2                     ............................................................
8780-N.discreta              ............................................................
958-G.calospora              ..............T....................G................GA......
5508-N.sublineolata          ..............T....................G................GA......
959-G.cerealis               ..............T.............................................
7221-N.pannonica             ..............T..............................C..............
1889-N.terricola             ..............T.............................................
1740-N.africana              ..............T...G...C.....................................
1739-N.galapagosensis        ..............T...G...C.....................................
1692-N.dodgei                ..............T...G...C.....................................
1910-N.lineolata             ..............T...G...C.........A...........................
M33876-N.crassaA             .............................................AA.............
```

```
                                     190       200       210       220       230       240
                             ....|....|....|....|....|....|....|....|....|....|....|....|
2489-N.crassaA               CGGTGTCTTTTCCATGAAGCCAAGCTCTTGCTATGCTCAACGAGAACGAAGTATCGCCAA
8900-N.crassaA               ............................................................
8830-N.crassaB               ............................................................
8858-N.crassaC               ..A.........................................................
8838-PS3                     ............................................................
8770-N.sitophila             ............................................................
8774-N.tetrasperma           ...............................................A............
8817-PS1                     ............................................................
8901-N.intermediaA           .................G..........................................
8844-N.intermediaB           .............................A..............................
8847-PS2                     .A..........................................................
8780-N.discreta              .............................A......A...AC...............G..
958-G.calospora              .A...C..............................A..GAA...T..............
5508-N.sublineolata          .....C..................A...........A..GAA...T..............
959-G.cerealis               .A..................................A...AA..............T...
7221-N.pannonica             .A..................................A...AA..................
1889-N.terricola             .A................A.................A...AA..................
1740-N.africana              .A..................................A..GAA..................
1739-N.galapagosensis        .A..................................A..GAA..................
1692-N.dodgei                .A..................................A..GAA..................
1910-N.lineolata             .A..................................A..GAA..................
M33876-N.crassaA             ............................................................
```

Supplemental Figure. Nucleotide alignment of the coding region of *mat A-2*

```
                                       250       260       270       280       290       300
                                  ....|....|....|....|....|....|....|....|....|....|....|....|
2489-N.crassaA                    AGCTGGTTCGGGTCTAGCAACGAGTTCGGATCTAGCGACGAGAGAAGAATCATCAAGACA
8900-N.crassaA                    ............................................................
8830-N.crassaB                    ............................................................
8858-N.crassaC                    .............................G...........................G..
8838-PS3                          ............................................................
8770-N.sitophila                  ............................................................
8774-N.tetrasperma                .............................G...........................G..
8817-PS1                          ............................................................
8901-N.intermediaA                .............................G...........................G..
8844-N.intermediaB                ..........................T..............................G..
8847-PS2                          ............................................................
8780-N.discreta                   ...........T.................G....T.........C.....T......G..
958-G.calospora                   ....................................A.......C............G..
5508-N.sublineolata               ....................................A.......C............G..
959-G.cerealis                    .....................------------------.....C............G..
7221-N.pannonica                  .............................G......A.......C............G..
1889-N.terricola                  .............................G......A.......C............G..
1740-N.africana                   ...........A........T....C...G......A.......C............G..
1739-N.galapagosensis             ...........A........T........G......A.......C............G..
1692-N.dodgei                     ...........A........T........G......A.......C............G..
1910-N.lineolata                  ...........A........T........G......A.......C...............
M33876-N.crassaA                  ............................................................

                                       310       320       330       340       350       360
                                  ....|....|....|....|....|....|....|....|....|....|....|....|
2489-N.crassaA                    TCATGCTGCATCATTGAGTCGACAAACACAATTCTTAACTTCCTCTCATTTCTTGAGAAG
8900-N.crassaA                    ............................................................
8830-N.crassaB                    ............................................................
8858-N.crassaC                    ............................................................
8838-PS3                          ............................................................
8770-N.sitophila                  ............................................................
8774-N.tetrasperma                ............................................................
8817-PS1                          ............................................................
8901-N.intermediaA                ............................................................
8844-N.intermediaB                ............................................................
8847-PS2                          ............................................................
8780-N.discreta                   ..G..................G.C........C..C..T..T..................
958-G.calospora                   ..G..........................G.....C.....T.....G..C......G..
5508-N.sublineolata               ..G..........................G.....C.....T.....G..C.........
959-G.cerealis                    ..G................................C.....T.....G..C.........
7221-N.pannonica                  ..G................................C.....T.....G..C.........
1889-N.terricola                  ..G................................C.....T.....G..C.........
1740-N.africana                   ...................................C.....T........C.........
1739-N.galapagosensis             ...................................C.....T........C.........
1692-N.dodgei                     ..G................................C.....T........C.........
1910-N.lineolata                  ..G................................C.....T........C.........
M33876-N.crassaA                  ............................................................
```

Supplemental Figure. Nucleotide alignment of the coding region of *mat A-2*

```
                                                 370       380       390       400       410       420
                                         ....|....|....|....|....|....|....|....|....|....|....|....|
2489-N.crassaA                           AATCGAGGATTGCCATTCGGTGGAGATCAAAGACTCCAACAAGCTGCCTACAAAGGCCAG
8900-N.crassaA                           ................CA..........................................
8830-N.crassaB                           ................CA..........................................
8858-N.crassaC                           ................CA..........................................
8838-PS3                                 ................CA..........................................
8770-N.sitophila                         ................CA..........................................
8774-N.tetrasperma                       ................CA..........................................
8817-PS1                                 ................CA......................G...................
8901-N.intermediaA                       ................CA..........................................
8844-N.intermediaB                       ................CA..........................................
8847-PS2                                 ................CA..........................................
8780-N.discreta                          ..A.............CA..C.......................................
958-G.calospora                          ..A........A....CA..C......G................................
5508-N.sublineolata                      ..A........A....CA..C......G................................
959-G.cerealis                           ..A.............CA..C.......................................
7221-N.pannonica                         ..A........A....CA..C.......................................
1889-N.terricola                         ..A.............CA..C..........................G............
1740-N.africana                          ..A.............CA..C.......G...T...........................
1739-N.galapagosensis                    ..A.............CA..C.......G...T...........................
1692-N.dodgei                            ..A.............CA..C.......G...T...........................
1910-N.lineolata                         ..A.............CA..C.......G...T....................G......
M33876-N.crassaA                         ............................................................
```

```
                                                 430       440       450       460       470       480
                                         ....|....|....|....|....|....|....|....|....|....|....|....|
2489-N.crassaA                           CAGTTTGCGTTCCGCCTCCTTCGCTCACTTACACTTCACAAAGCTGCTCAGGAGGTTCCG
8900-N.crassaA                           ............................................................
8830-N.crassaB                           ............................................................
8858-N.crassaC                           ...........................................A................
8838-PS3                                 ............................................................
8770-N.sitophila                         ......A....G................................................
8774-N.tetrasperma                       ...........................................A................
8817-PS1                                 ..........................T.................................
8901-N.intermediaA                       ...........................................A................
8844-N.intermediaB                       ...........................................A................
8847-PS2                                 ............................................................
8780-N.discreta                          .................................TGC.......A...........C...C
958-G.calospora                          .....C..A........................A.........A.........AA....C
5508-N.sublineolata                      .....C..A........................A....T....A.........AA....C
959-G.cerealis                           .....C..A.....................T..A.........T..........A....C
7221-N.pannonica                         .....C..A........................A.........A..........A....C
1889-N.terricola                         .....C..A........................A.........A..........A....C
1740-N.africana                          .....C..A..............A.........A.........A..........A....C
1739-N.galapagosensis                    .....C..A..............A.........A.........A..........A....C
1692-N.dodgei                            .....C..A..............A.......A.A.........A..........A....C
1910-N.lineolata                         .....C..A..............A.......A.A.........A..........A....C
M33876-N.crassaA                         ............................................................
```

Supplemental Figure. Nucleotide alignment of the coding region of *mat A-2*

```
                                   490       500       510       520       530       540
                              ....|....|....|....|....|....|....|....|....|....|....|....|
2489-N.crassaA                GGAAAGGACTTTGGCTTGGTCTACGGAAAAGATGTGTACGTACTGAATGGACATATTTTG
8900-N.crassaA                ............................................................
8830-N.crassaB                ............................................................
8858-N.crassaC                ......................................................C.....
8838-PS3                      ............................................................
8770-N.sitophila              ............................................................
8774-N.tetrasperma            ......................................................C.....
8817-PS1                      ............................................................
8901-N.intermediaA            ......................................................C.....
8844-N.intermediaB            ......................................................C.....
8847-PS2                      ............................................................
8780-N.discreta               ........A.......................G.....T...A..G....T...C.....
958-G.calospora               ....G...A.......................G...C.T...A.C.....T.T.C.A..T
5508-N.sublineolata           ....G...A.......................G...C.T...A.C.....T.T.C.A..T
959-G.cerealis                ....G...A.......................G.....T...T..G....T...C.....
7221-N.pannonica              ....G...A..............T........G.....T...A..G....T...C.....
1889-N.terricola              ....G...A.......................G.....T...A..G....T...C.....
1740-N.africana               ........A.....A...........C.....G.....T...A..G....T...C.....
1739-N.galapagosensis         ........A.....A...........C.....G.....T...A..G....T...C.....
1692-N.dodgei                 ........A.....A.................G.....T...A..G....T...C.....
1910-N.lineolata              ........A.....A.................G.....T...A..G....T...C.....
M33876-N.crassaA              ............................................................

                                   550       560       570       580       590       600
                              ....|....|....|....|....|....|....|....|....|....|....|....|
2489-N.crassaA                CACAGGTCGAAGCAAGAGATCGTGGGGCAGGCGGGAGGAAGAAACTGGCATGTCGACCAT
8900-N.crassaA                .....A......................................................
8830-N.crassaB                .....A......................................................
8858-N.crassaC                ............................................................
8838-PS3                      .....A......................................................
8770-N.sitophila              ............................................................
8774-N.tetrasperma            ............................................................
8817-PS1                      ....A.......................................................
8901-N.intermediaA            ............................................................
8844-N.intermediaB            ............................................................
8847-PS2                      ............................................................
8780-N.discreta               ....A...A..........................................A........
958-G.calospora               ........A.................A........G..G.AG.........A........
5508-N.sublineolata           ........A.................A........G..G.AG.........A........
959-G.cerealis                ........A.........G.....................AG.........A.T......
7221-N.pannonica              ........A.........G.............T.......AG.........A.T......
1889-N.terricola              ........A.........G.....................AG.........A.T......
1740-N.africana               ........A.........G.....................AG........CA.T......
1739-N.galapagosensis         ........A.........G.....................AG........CA.T......
1692-N.dodgei                 ........A.........G.....................AG........CA.T......
1910-N.lineolata              ........A.........G.....................AG........CA.T......
M33876-N.crassaA              ............................................................
```

Supplemental Figure. Nucleotide alignment of the coding region of *mat A-2*

```
                                610       620       630       640       650       660
                       ....|....|....|....|....|....|....|....|....|....|....|....|
2489-N.crassaA         ACCCTCCATCCTTTGAGGCGCGTTCCAGGCACCCCATGGCACAAGTTCTTTGGCAATCTT
8900-N.crassaA         ............................................................
8830-N.crassaB         ...............................................T............
8858-N.crassaC         ...................................G........................
8838-PS3               ............................................................
8770-N.sitophila       ............................................................
8774-N.tetrasperma     ............................................................
8817-PS1               ............................................................
8901-N.intermediaA     ............................................................
8844-N.intermediaB     ............................................................
8847-PS2               ............................................................
8780-N.discreta        ..T.....C..............C....................................
958-G.calospora        ..T.....C...............................................C...
5508-N.sublineolata    ..T.....C...............................................C...
959-G.cerealis         .TT.....C................................................T..
7221-N.pannonica       ..T.........................................................
1889-N.terricola       ..T.....C...................................................
1740-N.africana        ..T.....C..............C....................................
1739-N.galapagosensis  ..T.....C..............C....................................
1692-N.dodgei          ..T.....C...................................................
1910-N.lineolata       ..T.....C......G..TA........................................
M33876-N.crassaA       ............................................................
```

```
                                670       680       690       700       710       720
                       ....|....|....|....|....|....|....|....|....|....|....|....|
2489-N.crassaA         GAAGTTGGCGACGACAAGCAACTTCGCCTCTTCGATGATGATGCGGCCGTCGACAGTTAC
8900-N.crassaA         ............................................................
8830-N.crassaB         ............................................................
8858-N.crassaC         ...........A.............................A..................
8838-PS3               ............................................................
8770-N.sitophila       ............................................................
8774-N.tetrasperma     ............................................................
8817-PS1               ............................................................
8901-N.intermediaA     ...........A.............................A..............C...
8844-N.intermediaB     ...........A.............................A..............C...
8847-PS2               ............................................................
8780-N.discreta        .......A..C............................A..A..T.T.......A.G.T
958-G.calospora        ....C..A.------...G......A............CT..A..T.T.........G.T
5508-N.sublineolata    ....C..A.------...G......A............CT..A..T.T.........G.T
959-G.cerealis         ..GA...A..C.A.....G......A................A..T.T.........G.T
7221-N.pannonica       .......A..C.......G......A................A..T.T...A.....G.T
1889-N.terricola       .......A..C.......G......A................A..T.T.........G.T
1740-N.africana        .......A..C..G....G......A............C...A.TT.T.........G.T
1739-N.galapagosensis  .......A..C..G....G......A............C...A.TT.T.........G.T
1692-N.dodgei          .......A..C..G....G......A............C...A.TT.T.........G.T
1910-N.lineolata       .......A..C..G....G......A............C...A.TT.T.........G.T
M33876-N.crassaA       ............................................................
```

Supplemental Figure. Nucleotide alignment of the coding region of *mat A-2*

```
                                  730       740       750       760       770       780
                         ....|....|....|....|....|....|....|....|....|....|....|....|
2489-N.crassaA           CGAGTCGGTCCTCAGAAGTTCTTTGTGGTTATTCCGGAAACTGCTGAATTT-ATTTTGGA
8900-N.crassaA           .A...........G.....................................-........
8830-N.crassaB           .A...........G.....................................-........
8858-N.crassaC           .A...........G.....................................-........
8838-PS3                 .A...........G.....................................-........
8770-N.sitophila         .............G.....................................-........
8774-N.tetrasperma       .............G....................................C-........
8817-PS1                 .............G.....................................-........
8901-N.intermediaA       .A...........G..............................C......-........
8844-N.intermediaB       .A...........G.....................................-........
8847-PS2                 .............G.....................................-........
8780-N.discreta          ........A....G....................................C-........
958-G.calospora          ....A...C.T..G..G...T.C......C.....................-...C....
5508-N.sublineolata      ....A...C.T..G......T.C......C.....................-...C....
959-G.cerealis           ....A...C....G......T.C...............T............-...C....
7221-N.pannonica         ....A...C....G......T.C............................-...C....
1889-N.terricola         ....A...C....G......T.C............................-...C....
1740-N.africana          ....C........G......T.C............................-...C....
1739-N.galapagosensis    ....C........G......T.C............................-...C....
1692-N.dodgei            ....C........G......T.C.......................T....-...C....
1910-N.lineolata         ....C........G......T.C.......................T....-...C....
M33876-N.crassaA         ...................................................T........
```

Intron 2 (↓ between positions 826 and 827)

```
                                  790       800       810       820       830       840
                         ....|....|....|....|....|....|....|....|....|....|....|....|
2489-N.crassaA           CGAAGTCAGCAGCGAGCATCAGAGAGTCGCTACAATTCACACAGAGAATGGACATGTCCA
8900-N.crassaA           ....A.......................................................
8830-N.crassaB           ....A.......................................................
8858-N.crassaC           ....A.T..................C.......G..........................
8838-PS3                 ....A.......................................................
8770-N.sitophila         ....A.......................................................
8774-N.tetrasperma       ....A............................G..........................
8817-PS1                 G...A.......................................................
8901-N.intermediaA       ....A.T.A..................T.....G..........................
8844-N.intermediaB       ....A.T..........................G...A............A.....A...
8847-PS2                 ....A.......................................................
8780-N.discreta          ....A............................G........G....G........C...
958-G.calospora          ....A......................T.....G............GGA.....C.C...
5508-N.sublineolata      ....A......................T.....G............GGA.....C.C...
959-G.cerealis           ....A......................T.....G............GG......C.C...
7221-N.pannonica         ....A......................T.....G............GG........C...
1889-N.terricola         ....A......................T..................GG......C.C...
1740-N.africana          ....A...........T....A.....T.....G............GG.C....C.C...
1739-N.galapagosensis    ....A...........T....A.....T.....G............GG.C....C.C...
1692-N.dodgei            ....A...........T....A.....T.....G............GG......C.C...
1910-N.lineolata         ....A...........T....A.....T.....G............GG......C.C...
M33876-N.crassaA         .............-..............................................
```

Supplemental Figure. Nucleotide alignment of the coding region of *mat A-2*

```
                                                   Intron 3
                                850       860       870↓      880       890       900
                           ....|....|....|....|....|....|....|....|....|....|....|....|
2489-N.crassaA             GCCGCCAGCACCGACATCCATTCAGCAAGAAGCTCTCCTCAGGAAGTTGGACTTTGCCAT
8900-N.crassaA             .............G.............G................................
8830-N.crassaB             .............G.............G................................
8858-N.crassaC             ...............G............................................
8838-PS3                   .............G.............G........T.......................
8770-N.sitophila           ............................................................
8774-N.tetrasperma         ...............G............................................
8817-PS1                   ............................................................
8901-N.intermediaA         ...............G....................T.......................
8844-N.intermediaB         ...............G............................................
8847-PS2                   ............................................................
8780-N.discreta            ........T......G..T...........G...............C.............
958-G.calospora            ...............G...CC.........G....G...TGA..................
5508-N.sublineolata        ...............G...C..........G....G...TGA..................
959-G.cerealis             ...............G..............G....G...T.....C...........A..
7221-N.pannonica           ...............G..............G....G...T....................
1889-N.terricola           ...............G..............G....G...T....................
1740-N.africana            ...............G......T.......G....G........................
1739-N.galapagosensis      ...............G......T.......G....G........................
1692-N.dodgei              ...............G..............G....G........................
1910-N.lineolata           ...............G..............G....G........................
M33876-N.crassaA           ............................................................

                                910       920       930       940       950       960
                           ....|....|....|....|....|....|....|....|....|....|....|....|
2489-N.crassaA             GACAACATCATTGCCTGGTTATGTTGTAGAAGGACAACCTGAGATTGTGTTTCATCATGA
8900-N.crassaA             ............................................................
8830-N.crassaB             ............................................................
8858-N.crassaC             ............C...............................................
8838-PS3                   ............................................................
8770-N.sitophila           ............................................................
8774-N.tetrasperma         ............T...............................................
8817-PS1                   ....................................................T.......
8901-N.intermediaA         ............C...............................................
8844-N.intermediaB         ............C.......................G.......................
8847-PS2                   ............................................................
8780-N.discreta            .........G..C........C.............................C........
958-G.calospora            .G..G.......C................................GA..C..........
5508-N.sublineolata        .G..G.....G.C................................GA..C..........
959-G.cerealis             ............C..............................G................
7221-N.pannonica           A...........C..............................G.G..............
1889-N.terricola           ............C..............................G................
1740-N.africana            ............C..............................G..........C.....
1739-N.galapagosensis      ............C..............................G..........C.....
1692-N.dodgei              ............C..............................G..........C.....
1910-N.lineolata           ............C..............................G..........C.....
M33876-N.crassaA           .......................................................T....
```

Supplemental Figure. Nucleotide alignment of the coding region of *mat A-2*

```
                                Intron 4
                                   |
                           970     v  980       990       1000      1010      1020
                       ....|....|....|....|....|....|....|....|....|....|....|....|
2489-N.crassaA         AGGCTTACGCCAGATCCCCGTTGACTACAGTCAGGAGCGCCCACTTAGCATTCTCTCCCA
8900-N.crassaA         ............................................................
8830-N.crassaB         ............................................................
8858-N.crassaC         .........................................AG...........T.....
8838-PS3               ............................................................
8770-N.sitophila       ............................................................
8774-N.tetrasperma     .........................................A..................
8817-PS1               ........A...................................................
8901-N.intermediaA     .........................................A............T.....
8844-N.intermediaB     .........................................A............T.....
8847-PS2               ............................................................
8780-N.discreta        ......G......G................C..........A...C.....C........
958-G.calospora        ................---..C..TC....C..........A.T....T.C.........
5508-N.sublineolata    ................---..C..TC....C..........A.T....T.C.........
959-G.cerealis         ........A...............T.....C..........A......T...........
7221-N.pannonica       ........................TC....C..........A.T....T.C.........
1889-N.terricola       ........................TC....C.........GA.T....T.C.........
1740-N.africana        G.......................T.....C.........GA.T.C..T.C.........
1739-N.galapagosensis  G.......................T.....C.........GA.T.C..T.C.........
1692-N.dodgei          G.......................T.....C.........GA.T.C..T.C.........
1910-N.lineolata       G.......................T.....C...A.....GA.T.C..T.C.........
M33876-N.crassaA       ............................................................


                           1030      1040      1050      1060      1070      1080
                       ....|....|....|....|....|....|....|....|....|....|....|....|
2489-N.crassaA         TGTTTTCACTCGACCCGCACTTTGGGGAGAGGGTTTGGAGCTTGCTGATCACTTCGACCC
8900-N.crassaA         ............................................................
8830-N.crassaB         ............................................................
8858-N.crassaC         .....................C.....G.....................A..........
8838-PS3               ............................................................
8770-N.sitophila       ............................................................
8774-N.tetrasperma     ...........................G.....................A..........
8817-PS1               ............................................................
8901-N.intermediaA     ...........................G.....................A..........
8844-N.intermediaB     ...........................G.....................A..........
8847-PS2               ............................................................
8780-N.discreta        ...........................G......C..........G...A....T.....
958-G.calospora        A.................G........G......C..........G..A..T..T.....
5508-N.sublineolata    A.................G........G......C..........G..A..T..T.....
959-G.cerealis         ...........................G......C..........G...A....T.....
7221-N.pannonica       ...........................G......C..........G...A....T.....
1889-N.terricola       ...........................G......C..........G...A....T.....
1740-N.africana        ...............T...........G......C..........G...A....T.....
1739-N.galapagosensis  ...............T...........G......C..........G...A....T.....
1692-N.dodgei          ...............T...........G......C..........G...A....T.....
1910-N.lineolata       ..G............T...........G..A...C..........G...A....T.....
M33876-N.crassaA       ............................................................
```

Supplemental Figure. Nucleotide alignment of the coding region of *mat A-2*

```
                                1090      1100      1110      1120
                        ....|....|....|....|....|....|....|....|...
2489-N.crassaA          GCGAGACGGTGTGCAGCAAGAGGAGCACATCTATTACATTTGA
8900-N.crassaA          ...........................................
8830-N.crassaB          ...........................................
8858-N.crassaC          ...........................................
8838-PS3                ...........................................
8770-N.sitophila        ...........................................
8774-N.tetrasperma      ...C.......................................
8817-PS1                .................................C.........
8901-N.intermediaA      ...........................................
8844-N.intermediaB      ...........................................
8847-PS2                ...........................................
8780-N.discreta         .......................................C...
958-G.calospora         T...TT.A..A.T.................T............
5508-N.sublineolata     T...TT.A..A.T.................T............
959-G.cerealis          ..............................T............
7221-N.pannonica        ............................G.T............
1889-N.terricola        ..............................T..C.........
1740-N.africana         ......T.....................G.T............
1739-N.galapagosensis   ......T.....................G.T............
1692-N.dodgei           ......T.....................G.T............
1910-N.lineolata        ......T.....................G.T....G.......
M33876-N.crassaA        ...........................................
```

Supplemental Figure. Amino acid alignment of *mat A-2*

```
                                                           Intron 1
                                910       20        30        40        50        60
                                 *                            ↓
                      ....|....|....|....|....|....|....|....|....|....|....|....|
2489-N.crassaA        MNLLNMQPKRSEQPAMFEENRASSQEGQDLEVMYKKLHQLQARLSRSVLSEAIKEFEENL
8900-N.crassaA        ............................................................
8830-N.crassaB        ............................................................
8858-N.crassaC        I.......R...................................................
8838-PS3              ............................................................
8770-N.sitophila      ............................................................
8774-N.tetrasperma    ..H.....R...................................................
8817-PS1              ............................................................
8901-N.intermediaA    I.......R...................................................
8844-N.intermediaB    I.......R....S..............G...............................
8847-PS2              ............................................................
8780-N.discreta       .D.I...LR.....VR.....V......................................
958-G.calospora       .D.S....R..GR.V.LK...T...................................G..
5508-N.sublineolata   .D.S....R..GR.V.LK...T...................................G..
959-G.cerealis        .D.I....R.....V.L....T................H.....................
7221-N.pannonica      .D.I....R....AV.L....T............Q....................L....
1889-N.terricola      ...I....R.....V.L....T......................................
1740-N.africana       .D.I....R....SV......T.........R..............AA............
1739-N.galapagosensis .D.I.T..R....SV......T.........R..............AA............
1692-N.dodgei         .D.I....R....SV......T.........R......*
1910-N.lineolata      .D.I....R....SV......T.........R..............AA............
M33876-N.CrassaA      .......................................................N....
```

```
                                                         100
                                                          *
                                 70        80        90       100       110       120
                      ....|....|....|....|....|....|....|....|....|....|....|....|
2489-N.crassaA        RCLFHEAKLLLCSTRTKYRQSWFGSSNEFGSSDERRIIKTSCCIIESTNTILNFLSFLEK
8900-N.crassaA        ............................................................
8830-N.crassaB        ............................................................
8858-N.crassaC        .......................................A....................
8838-PS3              ............................................................
8770-N.sitophila      ............................................................
8774-N.tetrasperma    .......................................A....................
8817-PS1              ............................................................
8901-N.intermediaA    .......................................A....................
8844-N.intermediaB    .......................................A....................
8847-PS2              Q...........................................................
8780-N.discreta       ............T......E...........I..S....A.......A............
958-G.calospora       Q...........TE.S................N.S....A...................E
5508-N.sublineolata   ........I...TE.S................N.S....A....................
959-G.cerealis        Q...........TK.............------.S....A....................
7221-N.pannonica      Q...........TK..................N.S....A....................
1889-N.terricola      Q.....T.....TK..................N.S....A....................
1740-N.africana       Q...........TE..............S...N.S....A....................
1739-N.galapagosensis Q...........TE..................N.S....A....................
1692-N.dodgei
1910-N.lineolata      Q...........TE..................N.S.........................
M33876-N.CrassaA      ............................................................
```

Supplemental Figure. Amino acid alignment of *mat A-2*

```
                                                          155                 179
                                  130       140       150   *   160       170   * 180
                              ....|....|....|....|....|....|....|....|....|....|....|....|
2489-N.crassaA                NRGLPFGGDQRLQQAAYKGQQFAFRLLRSLTLHKAAQEVPGKDFGLVYGKDVYVLNGHIL
8900-N.crassaA                .....S......................................................
8830-N.crassaB                .....S......................................................
8858-N.crassaC                .....S............................D.......................L.
8838-PS3                      .....S......................................................
8770-N.sitophila              .....S................TL....................................
8774-N.tetrasperma            .....S............................D.......................L.
8817-PS1                      .....S.......R..............................................
8901-N.intermediaA            .....S............................D.......................L.
8844-N.intermediaB            .....S............................D.......................L.
8847-PS2                      .....S......................................................
8780-N.discreta               K....S.........................C..D...A...E.......E...MD..L.
958-G.calospora               K....S...E.....................I..D...I..RE.......E.H.I..LLF
5508-N.sublineolata           K....S...E.....................I..D...I..RE.......E.H.I..LLF
959-G.cerealis                K....S........................SI..V...I..RE.......E....D..L.
7221-N.pannonica              K....S.........................I..D...I..RE.......E...MD..L.
1889-N.terricola              K....S.........................I..D...I..RE.......E...MD..L.
1740-N.africana               K....S...RS....................I..D...I...E.......E...MD..L.
1739-N.galapagosensis         K....S...RS....................I..D...I...E.......E...MD..L.
1692-N.dodgei
1910-N.lineolata              K....S...RS...................KI..D...I...E.......E...MD..L.
M33876-N.CrassaA              ............................................................
```

```
                                                                  HPG-domain
                                            ________________________________________________
                                  190       200       210       220       230       240
                              ....|....|....|....|....|....|....|....|....|....|....|....|
2489-N.crassaA                HRSKQEIVGQAGGRNWHVDHTLHPLRRVPGTPWHKFFGNLEVGDDKQLRLFDDDAAVDSY
8900-N.crassaA                ............................................................
8830-N.crassaB                ............................................................
8858-N.crassaC                ...........................................E.........E......
8838-PS3                      ............................................................
8770-N.sitophila              ............................................................
8774-N.tetrasperma            ............................................................
8817-PS1                      .K..........................................................
8901-N.intermediaA            ...........................................E.........E......
8844-N.intermediaB            ...........................................E.........E......
8847-PS2                      ............................................................
8780-N.discreta               .K...............I........................DA.........NTS..ND
958-G.calospora               .............K...I.......................AD--.E.H....YTS...D
5508-N.sublineolata           .............K...I.......................AD--.E.H....YTS...D
959-G.cerealis                ......V......K...I..I..................F.IDAN.E.H.....TS...D
7221-N.pannonica              ......V......K...I........................DA..E.H.....TS.N.D
1889-N.terricola              ......V......K...I........................DA..E.H.....TS...D
1740-N.africana               ......V......K...I........................DAG.E.H.....TS...D
1739-N.galapagosensis         ......V......K...I........................DAG.E.H.....TS...D
1692-N.dodgei
1910-N.lineolata              ......V......K...I.......GY...............DAG.E.H.....TS...D
M33876-N.CrassaA              ............................................................
```

Supplemental Figure. Amino acid alignment of *mat A-2*

```
                                                        Intron 2        Intron 3
                             250       260       270       280       290       300
                      ....|....|....|....|....|....|....|....|....|....|....|....|
2489-N.crassaA        RVGPQKFFVVIPETAEFILDEVSSEHQRVATIHTENGHVQPPAPTSIQQEALLRKLDFAM
8900-N.crassaA        Q...R................I......................A...............
8830-N.crassaB        Q...R................I......................A...............
8858-N.crassaC        Q...R................I......L...............................
8838-PS3              Q...R................I......................A...............
8770-N.sitophila      ....R................I......................................
8774-N.tetrasperma    ....R................I......................................
8817-PS1              ....R..............E.I......................................
8901-N.intermediaA    Q...R................IN.....................................
8844-N.intermediaB    Q...R................I..........N...E.D.....................
8847-PS2              ....R................I......................................
8780-N.discreta       ....R................I.............S..A..V.................
958-G.calospora       .D.LRR.S.............I.............G..A.......T....R.E......
5508-N.sublineolata   .D.LR..S.............I.............G..A.......T....R.E......
959-G.cerealis        .D..R..S....D........I.............G..A............R..N.....
7221-N.pannonica      .D..R..S.............I.............G..A............R.......I
1889-N.terricola      .D..R..S.............I.............G..A............R........
1740-N.africana       .A..R..S.............I...Y.........GR.A........*
1739-N.galapagosensis .A..R..S.............I...Y.........GR.A........*
1692-N.dodgei
1910-N.lineolata      .A..R..S.......V.....I...Y.........G..A............R........
M33876-N.CrassaA      .................YFGRSQQQ...................................
```

```
                                  Intron 4
                         304                      334                    357
                          *  310       320       330 *     340       350   * 360
                      ....|....|....|....|....|....|....|....|....|....|....|....|
2489-N.crassaA        TTSLPGYVVEGQPEIVFHHEGLRQIPVDYSQERPLSILSHVFTRPALWGEGLELADHFDP
8900-N.crassaA        ............................................................
8830-N.crassaB        ............................................................
8858-N.crassaC        ...F.............................Q......................N...
8838-PS3              ............................................................
8770-N.sitophila      ............................................................
8774-N.tetrasperma    ...F.............................Q......................N...
8817-PS1              .................Y....H.....................................
8901-N.intermediaA    ...F.............................Q......................N...
8844-N.intermediaB    ...F.............................Q......................N...
8847-PS2              ............................................................
8780-N.discreta       ...F....................V........Q......................N...
958-G.calospora       AA.F..........MML........-..H....QF.T..Q...............E....
5508-N.sublineolata   AA.V..........MML........-..H....QF.T..Q...............E....
959-G.cerealis        ...F..........V.......H..........Q......................N...
7221-N.pannonica      ...F..........V.............H....QF.T...................N...
1889-N.terricola      ...F..........V.............H....EF.T...................N...
1740-N.africana
1739-N.galapagosensis
1692-N.dodgei
1910-N.lineolata      ...F..........V................K.EF.T...G...............N...
M33876-N.CrassaA      ..................Y.........................................
```

Supplemental Figure. Amino acid alignment of *mat A-2*

```
                               370
                         ....|....|....
2489-N.crassaA           RDGVQQEEHIYYI*
8900-N.crassaA           .............*
8830-N.crassaB           .............*
8858-N.crassaC           .............*
8838-PS3                 .............*
8770-N.sitophila         .............*
8774-N.tetrasperma       .............*
8817-PS1                 .............*
8901-N.intermediaA       .............*
8844-N.intermediaB       .............*
8847-PS2                 .............*
8780-N.discreta          .............*
958-G.calospora          .FSI.........*
5508-N.sublineolata      .FSI.........*
959-G.cerealis           .............*
7221-N.pannonica         .........V...*
1889-N.terricola         .............*
1740-N.africana
1739-N.galapagosensis
1692-N.dodgei
1910-N.lineolata         .........V.C.*
M33876-N.CrassaA         .............*
```

Supplemental Figure. Nucleotide alignment of the coding region of *mat A-3*

```
                                  10        20        30        40        50        60
                         ....|....|....|....|....|....|....|....|....|....|....|....|
2489-N.crassaA           ATGTCTGCCTTAGACGTTGATTCAATCAGCGACATCGCACCCGGTCTCAGCCCTGTGACT
8900-N.crassaA           ...........T.........G......................................
8830-N.crassaB           ...C.......T.........G......................................
8858-N.crassaC           .....................G......C...............................
8838-PS3                 ...........T.........G......................................
8770-N.sitophila         .....................G......................................
8774-N.tetrasperma       .....................G..............A.......................
8817-PS1                 .....................G......................................
8901-N.intermediaA       ....................AG......C...................G...........
8844-N.intermediaB       ....................AG......C...............................
8847-PS2                 .....................G......................................
8780-N.discreta          .....................G......................................
958-G.calospora          .....CA........C....CA................G........TCC......A...
5508-N.sublineolata      .....CA........C....CA................G........TCC......A...
959-G.cerealis           ......A.............CA.G................................A...
7221-N.pannonica         ......A.............CA.G................................A...
1740-N.africana          ......A..............G..................................A...
1739-N.galapagosensis    ......A..............G..................................A...
1692-N.dodgei            ..A...A..............G.....G............................A...
1910-N.lineolata         ......A..............G.....G..............T.............A...
M33876-N.crassaA         ............................................................
```

```
                                  70        80        90       100       110       120
                         ....|....|....|....|....|....|....|....|....|....|....|....|
2489-N.crassaA           GCAATTCACTATGGCAGGATCCAGGTAATGCTGTTCAGGTCCCATCTGGCCGAATTTGCT
8900-N.crassaA           ...............................................A............
8830-N.crassaB           ...............................................A............
8858-N.crassaC           ..........................................................T.
8838-PS3                 ...............................................A............
8770-N.sitophila         ............................................................
8774-N.tetrasperma       ............................................................
8817-PS1                 ............................................................
8901-N.intermediaA       ......................................A.....................
8844-N.intermediaB       .......................A..G.................................
8847-PS2                 ............................................................
8780-N.discreta          ..CC............................C.....A..............T......
958-G.calospora          ..C....G.........A...................................T....T.
5508-N.sublineolata      ..C....G.........A................................T..T....T.
959-G.cerealis           ..CC.................................................T..CTT.
7221-N.pannonica         ..CC...............................T.................T..CTT.
1740-N.africana          ..CC.............................................T...T..C...
1739-N.galapagosensis    ..CC.............................................T...T..C...
1692-N.dodgei            ..CC...................TC........................T...T..C...
1910-N.lineolata         ..CC.............................................T...T..C...
M33876-N.crassaA         ............................................................
```

Supplemental Figure. Nucleotide alignment of the coding region of *mat A-3*

```
                                   Intron 1
                        130       140       150 ↓     160       170       180
                    ....|....|....|....|....|....|....|....|....|....|....|....|
2489-N.crassaA      GAAGAGGATCTTGTCTATGCGATGGACAACTCAGTTGTCGTGTTTGGCGAAGAGGCCTTG
8900-N.crassaA      ............................................................
8830-N.crassaB      ............................................................
8858-N.crassaC      ............................................................
8838-PS3            ............................................................
8770-N.sitophila    ............................................................
8774-N.tetrasperma  ............................................................
8817-PS1            ............................................................
8901-N.intermediaA  ............................................................
8844-N.intermediaB  ............................................................
8847-PS2            ............................................................
8780-N.discreta     .........................................A..............T...
958-G.calospora     .....................................C...A..............T...
5508-N.sublineolata .....................................C...A..............T...
959-G.cerealis      .....................................C...A..............TC..
7221-N.pannonica    ....................A................C...A..............T...
1740-N.africana     .....................................C...A..............T...
1739-N.galapagosensis .....................................C...A..............T...
1692-N.dodgei       .....................................C...A..............T..A
1910-N.lineolata    .....................................C...A.......G......T...
M33876-N.crassaA    ............................................................
```

```
                        190       200       210       220       230       240
                    ....|....|....|....|....|....|....|....|....|....|....|....|
2489-N.crassaA      CTCATGGTTGCCCCCGACGAGTCCAGCATCGCGATCTGCACGTATCCATTTGGGCTCATG
8900-N.crassaA      .................T..............................G...........
8830-N.crassaB      .................T..............................G...........
8858-N.crassaC      .................T..............................G...........
8838-PS3            .................T..............................G...........
8770-N.sitophila    .................T..............................G...........
8774-N.tetrasperma  .................T..............................G...........
8817-PS1            .................T..............................G...........
8901-N.intermediaA  .................T..............................G...........
8844-N.intermediaB  .................T..............................G...........
8847-PS2            .................T..............................G...........
8780-N.discreta     ..T..............T...A.A...........T............G....A......
958-G.calospora     .................T...A.A..T........A............G........G..
5508-N.sublineolata .................T...A.A..T........A............G........G..
959-G.cerealis      .................T...A.A..T........T............G...........
7221-N.pannonica    .................T...A.A..T........T............G...........
1740-N.africana     ..T..............T...A.A.....T.....T............G...........
1739-N.galapagosensis ..T..............T...A.A.....T.....T............G...........
1692-N.dodgei       ..T..............T...A.A.....T.....T............G...........
1910-N.lineolata    ..T..............T...A.A.....T.....T............G...........
M33876-N.crassaA    ............................................................
```

Supplemental Figure. Nucleotide alignment of the coding region of *mat A-3*

```
                                                                  Intron 2
                                                                     ↓
                                  250       260       270       280       290       300
                             ....|....|....|....|....|....|....|....|....|....|....|....|
2489-N.crassaA               ATGATGGAATGGGGAAACTGGGACATACTCGCAGTTTCGCCCCCTTCGCGAACTCCCACC
8900-N.crassaA               .................................................A..........
8830-N.crassaB               .................................................A..........
8858-N.crassaC               .........................................G.......A..........
8838-PS3                     .................................................A..........
8770-N.sitophila             .................................................A..........
8774-N.tetrasperma           ...............................................A.A..........
8817-PS1                     .................................................A..........
8901-N.intermediaA           .........................................G.......A.........T
8844-N.intermediaB           .........................................G.......A..........
8847-PS2                     .................................................A..A.......
8780-N.discreta              ...G.........................................C...A......T...
958-G.calospora              .......................T...........C.....T...C.T.....C..TG.T
5508-N.sublineolata          ...................................C.....T...C.T.....C..TG.T
959-G.cerealis               .........................................T...C...A...C..TG..
7221-N.pannonica             .........................................T...C...A...C..TG..
1740-N.africana              .........................................T...C...A......TG.-
1739-N.galapagosensis        .........................................T...C...A......TG.-
1692-N.dodgei                .........................................T...C...A......TG..
1910-N.lineolata             .........................................T...C...A......TG..
M33876-N.crassaA             ............................................................

                                  310       320       330       340       350       360
                             ....|....|....|....|....|....|....|....|....|....|....|....|
2489-N.crassaA               ATTCCAAGCGAGAGCGTCTTGGGCATCTCAAACCAAGGAGGTGCTAATGTTGAGCAGCAA
8900-N.crassaA               ............................................................
8830-N.crassaB               ............................................................
8858-N.crassaC               .....G............................................C.........
8838-PS3                     ............................................................
8770-N.sitophila             .....................................................C......
8774-N.tetrasperma           .............................G....T.........................
8817-PS1                     ............................................................
8901-N.intermediaA           .....G..................................A...C.....C.........
8844-N.intermediaB           .....G..........................T.................C.........
8847-PS2                     ............................................................
8780-N.discreta              .....CG......A.AC....A..........................CCC.........
958-G.calospora              G....CGT......T.C....A..........................CCC.........
5508-N.sublineolata          G....CGT......T.C....A..........T...............CCC.........
959-G.cerealis               .....CGT.....AT.C....A..........................CCC.........
7221-N.pannonica             .....CGT......T.C....A..........................CCC.........
1740-N.africana              ------------------------------------------------------------
1739-N.galapagosensis        ------------------------------------------------------------
1692-N.dodgei                .....CGT.....T..C....A..........................CCC.........
1910-N.lineolata             .....CGT.....T..C....A....................T.....CCC.........
M33876-N.crassaA             ............................................................
```

Supplemental Figure. Nucleotide alignment of the coding region of *mat A-3*

```
                                370       380       390       400       410       420
                            ....|....|....|....|....|....|....|....|....|....|....|....|
2489-N.crassaA              GAGCAAAGCTCTCATACCATCGACATGACTCTGCCCAGCAACTTTTTTGAACAGAGTTCG
8900-N.crassaA              ................................A....A......................
8830-N.crassaB              ................................A....A......................
8858-N.crassaC              .....................................A......................
8838-PS3                    ................................A....A......................
8770-N.sitophila            .....................................A......................
8774-N.tetrasperma          .....................................A......................
8817-PS1                    ..............A......................A......................
8901-N.intermediaA          ...........................G.........A......................
8844-N.intermediaB          .......................A.............A......................
8847-PS2                    .....................................A......................
8780-N.discreta             ...........C......G..................TT.....................
958-G.calospora             ....G......C..............T.T.......GA.....AC...........C...
5508-N.sublineolata         ....G......C................T.......GA.....AC...........C...
959-G.cerealis              ....G......C............GC...........A.....A............C...
7221-N.pannonica            ....G......C..........G.GC...........A.....AC...........C...
1740-N.africana             ------------------------------------------------------------
1739-N.galapagosensis       ------------------------------------------------------------
1692-N.dodgei               ....G......C............GC......A...GA.....A............C...
1910-N.lineolata            ....G......C............GC......A...GA.....A............C...
M33876-N.crassaA            ............................................................
```

```
                                430       440       450       460       470       480
                            ....|....|....|....|....|....|....|....|....|....|....|....|
2489-N.crassaA              GTAACTCAAAGCAATGGTACTAGCCGCCCGCGCAACCAATTCGTTCTCTACTACCAATGG
8900-N.crassaA              .............................T........G..T..................
8830-N.crassaB              .........................................T..................
8858-N.crassaC              ............................................................
8838-PS3                    .........................................T..................
8770-N.sitophila            ............................................................
8774-N.tetrasperma          ............................................................
8817-PS1                    ............................................................
8901-N.intermediaA          ............................................................
8844-N.intermediaB          ............................................................
8847-PS2                    ............................................................
8780-N.discreta             .....C..G....................T........G.....................
958-G.calospora             ..C.A..TG.........T..........T.....T..G..T................C.
5508-N.sublineolata         ..C.A..TG.........T..........T.....T..G..T...........T....C.
959-G.cerealis              ........G.........T..........T.....T..G.....................
7221-N.pannonica            ........G.........T..........T.....T..G.....................
1740-N.africana             ------------------------------------------------------------
1739-N.galapagosensis       ------------------------------------------------------------
1692-N.dodgei               ........G.........T....T.....T.....T..G.....................
1910-N.lineolata            ........G.........T....T.....T.....T..G.....................
M33876-N.crassaA            ............................................................
```

Supplemental Figure. Nucleotide alignment of the coding region of *mat A-3*

```
                                                      Intron 3
                             490       500       510       530       540
                                                 520          |
                        ....|....|....|....|....|....|....|....|....|....|....|....|
2489-N.crassaA          CTGTTGGATACTCTGTTCTCCGAAGATCCGAGTCTTTCAGCTCGCAATATTTCTCAAATT
8900-N.crassaA          .....A......................................................
8830-N.crassaB          ............................................................
8858-N.crassaC          ............................................................
8838-PS3                ............................................................
8770-N.sitophila        ............................................................
8774-N.tetrasperma      ............................................................
8817-PS1                ............................................................
8901-N.intermediaA      ............................................................
8844-N.intermediaB      ..............................................G.............
8847-PS2                ............................................................
8780-N.discreta         ..C..........................A.....A.........G...........G..
958-G.calospora         ........C....................A.....C.........G.A..C......G..
5508-N.sublineolata     ........C....................A.....C.........G.A..C......G..
959-G.cerealis          ............A.T............T.A.....C.........G....C......G..
7221-N.pannonica        ..............T.........A....A.....C.........G....C......G..
1740-N.africana         -----------------------------------------------------....G..
1739-N.galapagosensis   -----------------------------------------------------....G..
1692-N.dodgei           ..............T..............A.....C.........G....C......G..
1910-N.lineolata        ..............T..............A.....C.........G....C......G..
M33876-N.crassaA        ............................................................


                             550       560       570       580       590       600
                        ....|....|....|....|....|....|....|....|....|....|....|....|
2489-N.crassaA          GTTGCGGGCTTGTGGAACAGCGAGCACCCCGCGGCAAAAGCTCGCTTCAGGGAACTGGCG
8900-N.crassaA          ................................A...........................
8830-N.crassaB          ................................A...........................
8858-N.crassaC          ............................................................
8838-PS3                ................................A...........................
8770-N.sitophila        ............................................................
8774-N.tetrasperma      .....................................................G......
8817-PS1                ............................................................
8901-N.intermediaA      ............................................................
8844-N.intermediaB      ............................................................
8847-PS2                ............................................................
8780-N.discreta         .................................................T..........
958-G.calospora         .......................A..............G..........TC........A
5508-N.sublineolata     ......................................G..........TC........A
959-G.cerealis          ......................................G..........T..........
7221-N.pannonica        ......................................G..........T..........
1740-N.africana         ......................................G..........T..........
1739-N.galapagosensis   ......................................G..........T..........
1692-N.dodgei           ......................................G..........T..........
1910-N.lineolata        ......................................G..........T..........
M33876-N.crassaA        ............................................................
```

Supplemental Figure. Nucleotide alignment of the coding region of *mat A-3*

```
                                   610       620       630       640       650       660
                         ....|....|....|....|....|....|....|....|....|....|....|....|
2489-N.crassaA           GAAATGGAGGTTCATCGTCATCGAGCTGAGAATCCTCACCTTTACCCCGACCAGCCGCGA
8900-N.crassaA           ....................C.......................................
8830-N.crassaB           ....................C.......................................
8858-N.crassaC           ..............G.....C.......................................
8838-PS3                 ....................C.......................................
8770-N.sitophila         ....................C.......................................
8774-N.tetrasperma       ..............G.....C.......................................
8817-PS1                 ....................C.......................................
8901-N.intermediaA       ..............G.....C.......................................
8844-N.intermediaB       ..............G.....C.......................................
8847-PS2                 ....................C.......................................
8780-N.discreta          ..............G.....C.......................................
958-G.calospora          ...........CGCG.....C.....C...........................T....G
5508-N.sublineolata      ...........CGCG.....C.....C...........................T.....
959-G.cerealis           ...........C.GG.....C.......................................
7221-N.pannonica         ...........C.GG.....C..................T....................
1740-N.africana          ...........C.GG.....C.................................T.C...
1739-N.galapagosensis    ...........C.GG.....C.................................T.C...
1692-N.dodgei            ...........C.GG.....C.................................T.C...
1910-N.lineolata         ...........C.GG.....C.......................A.........T.C...
M33876-N.crassaA         ............................................................
```

```
                                   670       680       690       700       710       720
                         ....|....|....|....|....|....|....|....|....|....|....|....|
2489-N.crassaA           TTTCCCACCACCGATCCCGTTCCTCCTCGCATGAGATATCCTTGCGTAATCTCGCCCGAA
8900-N.crassaA           ............................................................
8830-N.crassaB           ..................................A.........................
8858-N.crassaC           ............................................................
8838-PS3                 ............................................................
8770-N.sitophila         ............................................................
8774-N.tetrasperma       ............................................................
8817-PS1                 ............................................................
8901-N.intermediaA       ..................................T.........................
8844-N.intermediaB       ............................................................
8847-PS2                 ............................................................
8780-N.discreta          ........G.....C.............................................
958-G.calospora          .....G..G...........C.........................A...T.........
5508-N.sublineolata      ........G...........C.........................A...T.T.......
959-G.cerealis           ........G...........C.........................A.............
7221-N.pannonica         ........G...........C.........................A.............
1740-N.africana          ........G...........C....................C.A..A.....T...T...
1739-N.galapagosensis    ........A...........C....................C.A..A.........T...
1692-N.dodgei            ........G...........C....T...............C....A.........T...
1910-N.lineolata         ........G...........C....................C....A.........T...
M33876-N.crassaA         ............................................................
```

Supplemental Figure. Nucleotide alignment of the coding region of *mat A-3*

```
                                730       740       750       760       770       780
                       ....|....|....|....|....|....|....|....|....|....|....|....|
2489-N.crassaA         GATCGGCAACGAATTTTGCGGATGCTCGACTTTGTCTGGGAAGAGTCGAACGGCCAGTTG
8900-N.crassaA         ...............C............................................
8830-N.crassaB         ...............C............................................
8858-N.crassaC         ...............C............................................
8838-PS3               ...............C............................................
8770-N.sitophila       ...............C............................................
8774-N.tetrasperma     ...............C............................................
8817-PS1               ...............C............................................
8901-N.intermediaA     ...............C............................................
8844-N.intermediaB     ...............C....A.......................................
8847-PS2               ...............C............................................
8780-N.discreta        ...............C.........................................C..
958-G.calospora        ...............C..........T..............................C..
5508-N.sublineolata    ...............C..........T..............................C..
959-G.cerealis         ...............C..........T..............................C..
7221-N.pannonica       ...............C..........T..............................C..
1740-N.africana        ...............C..........T..............................C..
1739-N.galapagosensis  ...............C..........T..............................C..
1692-N.dodgei          ...............C..........T..............................C..
1910-N.lineolata       ...............C..........T...........C..................C..
M33876-N.crassaA       .....C......................................................

                                790       800       810       820       830       840
                       ....|....|....|....|....|....|....|....|....|....|....|....|
2489-N.crassaA         GCTGCCGAAGAAGCCGCACTTAACGATGTTGTTCAACCTCAACAAGCCGAGGAGGTCGGC
8900-N.crassaA         ............................................................
8830-N.crassaB         ............................................................
8858-N.crassaC         ..............................................T.............
8838-PS3               ............................................................
8770-N.sitophila       ..............................T.............................
8774-N.tetrasperma     ............................................................
8817-PS1               ............................................................
8901-N.intermediaA     ............................................................
8844-N.intermediaB     ............................................................
8847-PS2               ............................................................
8780-N.discreta        ..................G........ACC.AA...A.C...G.......A.......A.
958-G.calospora        ..................G.........CC.....G..C...................C.
5508-N.sublineolata    ..................G.........CC........C...................C.
959-G.cerealis         ..................G.........CC........C........T............
7221-N.pannonica       ..................G.........CC........C.....................
1740-N.africana        ..................G.........CC........C.....G.........A.....
1739-N.galapagosensis  ..................G.........CC........C.....G.........A.....
1692-N.dodgei          ..................G.........CC........C.....G.........A.....
1910-N.lineolata       ..................G.........CC........C.....G.........A.....
M33876-N.crassaA       ............................................................
```

Supplemental Figure. Nucleotide alignment of the coding region of *mat A-3*

```
                                  850       860       870       880       890       900
                         ....|....|....|....|....|....|....|....|....|....|....|....|
2489-N.crassaA           CCTTTCCCCGACTTCGAGTGGGAGGAGCCTAACCACATTATCGACATGTCGACCGACCTG
8900-N.crassaA           ............................................................
8830-N.crassaB           ............................................................
8858-N.crassaC           ............................................................
8838-PS3                 ............................................................
8770-N.sitophila         ............................................................
8774-N.tetrasperma       ............................................................
8817-PS1                 ............................................................
8901-N.intermediaA       ............................................................
8844-N.intermediaB       ............................................................
8847-PS2                 ............................................................
8780-N.discreta          .............A...............C.....G.....T..................
958-G.calospora          .............................C.....GG.C..................T.A
5508-N.sublineolata      .............................C.....G..C..................T.A
959-G.cerealis           .............A...............C.....G..C..T...............T..
7221-N.pannonica         .............A...............C.....G..C..T...............T..
1740-N.africana          .............A...............C.C...G..C..T...............T..
1739-N.galapagosensis    .............A...............C.C...G..C..T...............T..
1692-N.dodgei            .............A...............C.....G..C..T...............T..
1910-N.lineolata         .............A...............C.....G..C..T...............T..
M33876-N.crassaA         ............................................................
```

```
                                  910       920       930       940       950       960
                         ....|....|....|....|....|....|....|....|....|....|....|....|
2489-N.crassaA           TCCGTGGCCCAGGACCCGGACTTCATGATGACTGAGGACGACTCCATGCGATTCCTTCTC
8900-N.crassaA           ............................................................
8830-N.crassaB           ............................................................
8858-N.crassaC           ............................................................
8838-PS3                 ............................................................
8770-N.sitophila         ............................................................
8774-N.tetrasperma       .........................................................T..
8817-PS1                 ............................................................
8901-N.intermediaA       .................................................A..........
8844-N.intermediaB       ............................................................
8847-PS2                 ............................................................
8780-N.discreta          ..G..A..G.......................................G...........
958-G.calospora          ........G.......................................GA......C...
5508-N.sublineolata      ........G.......................................GA......C..T
959-G.cerealis           ........G.................A.....................G...........
7221-N.pannonica         ........G.......................................G...........
1740-N.africana          ........G....................A..................G...........
1739-N.galapagosensis    ........G....................A..................G...........
1692-N.dodgei            ........G.......................................G...........
1910-N.lineolata         ........G.......................................G...........
M33876-N.crassaA         ............................................................
```

Supplemental Figure. Nucleotide alignment of the coding region of *mat A-3*

```
                                       970
                              ....|....|....|
2489-N.crassaA                AAGCAGGCGTCCTGA
8900-N.crassaA                ..........G....
8830-N.crassaB                ..........G....
8858-N.crassaC                ..........G....
8838-PS3                      ..........G....
8770-N.sitophila              ..........G....
8774-N.tetrasperma            .G........G....
8817-PS1                      ..........G....
8901-N.intermediaA            ..........G....
8844-N.intermediaB            ..........G....
8847-PS2                      ..........G....
8780-N.discreta               ..........G....
958-G.calospora               .C........G..A.
5508-N.sublineolata           ..........G..A.
959-G.cerealis                ..........G....
7221-N.pannonica              ..........G....
1740-N.africana               ..........G....
1739-N.galapagosensis         ..........G....
1692-N.dodgei                 ..........G....
1910-N.lineolata              ..........G....
M33876-N.crassaA              ...............
```

Supplemental Figure. Amino acid alignment of *mat A-3*

```
                                                                     Intron 1
                               10        20        30        40        50        60
                       ....|....|....|....|....|....|....|....|....|....|....|....|
2489-N.crassaA         MSALDVDSISDIAPGLSPVTAIHYGRIQVMLFRSHLAEFAEEDLVYAMDNSVVVFGEEAL
8900-N.crassaA         ...F...A....................................................
8830-N.crassaB         .P.F...A....................................................
8858-N.crassaC         .......A.T.............................V....................
8838-PS3               ...F...A....................................................
8770-N.sitophila       .......A....................................................
8774-N.tetrasperma     .......A....T...............................................
8817-PS1               .......A....................................................
8901-N.intermediaA     ......EA.T......G...........................................
8844-N.intermediaB     ......EA.T..................................................
8847-PS2               .......A....................................................
8780-N.discreta        .......A.............L...............D......................
958-G.calospora        ..T..L.T........P.....R...............D.V............A.......
5508-N.sublineolata    ..T..L.T........P.....R...............D.V............A.......
959-G.cerealis         ..T....T.............L...............D.F............A.......
7221-N.pannonica       ..T....T.............L...............D.F............A.......
1740-N.africana        ..T....A.............L..............VD..............A.......
1739-N.galapagosensis  ..T....A.............L..............VD..............A.......
1692-N.dodgei          I.T....A.G...........L.....HL.......VD..............A.......
1910-N.lineolata       ..T....A.G....C......L..............VD..............A...G...
M33876-N.crassaA       ............................................................

                                                            Intron 2
                               70        80        90       100       110       120
                       ....|....|....|....|....|....|....|....|....|....|....|....|
2489-N.crassaA         LMVAPDESSIAICTYPFGLMMMEWGNWDILAVSPPSRTPTIPSESVLGISNQGGANVEQQ
8900-N.crassaA         ................V...................Q.......................
8830-N.crassaB         ................V...................Q.......................
8858-N.crassaC         ................V...................Q.......................
8838-PS3               ................V...................Q.......................
8770-N.sitophila       ................V...................Q....................D..
8774-N.tetrasperma     ................V...................Q..............L........
8817-PS1               ................V...................Q.......................
8901-N.intermediaA     ................V...................Q................D......
8844-N.intermediaB     ................V...................Q.......................
8847-PS2               ................V...................QN......................
8780-N.discreta        .......T........V....V.............PQ.....G.NT.S........P...
958-G.calospora        .......T........V..V...............P...AV.V..A.S........P...
5508-N.sublineolata    .......T........V..V...............P...AV.V..A.S........P...
959-G.cerealis         .......T........V..................PQ..A..V.NA.S........P...
7221-N.pannonica       .......T........V..................PQ..A..V..A.S........P...
1740-N.africana        .......T........V..................PQ..---------------------
1739-N.galapagosensis  .......T........V..................PQ..---------------------
1692-N.dodgei          .......T........V..................PQ..A..V.IA.S........P...
1910-N.lineolata       .......T........V..................PQ..A..V.IA.S......S.P...
M33876-N.crassaA       ............................................................
```

Supplemental Figure. Amino acid alignment of *mat A-3*

```
                                                               HMG-box
                                                                              Intron 3
                                  130       140       150       160       170       180
                         ....|....|....|....|....|....|....|....|....|....|....|....|
2489-N.crassaA           EQSSHTIDMTLPSNFFEQSSVTQSNGTSRPRNQFVLYYQWLLDTLFSEDPSLSARNISQI
8900-N.crassaA           ............N...............................................
8830-N.crassaB           ............N...............................................
8858-N.crassaC           ............N...............................................
8838-PS3                 ............N...............................................
8770-N.sitophila         ............N...............................................
8774-N.tetrasperma       ............N...............................................
8817-PS1                 ....Q.......N...............................................
8901-N.intermediaA       .........A..N...............................................
8844-N.intermediaB       .......E....N..........................................S....
8847-PS2                 ............N...............................................
8780-N.discreta          ......V.....I..........................................D...V
958-G.calospora          .R......II..D.Y......NL...S............S...............E...V
5508-N.sublineolata      .R.......I..D.Y......NL...S............S...............E...V
959-G.cerealis           .R......A...N.Y...........S.................I....S.....D...V
7221-N.pannonica         .R.....GA...N.Y...........S.....................N......D...V
1740-N.africana          ---------------------------------------------------------A.V
1739-N.galapagosensis    ---------------------------------------------------------A.V
1692-N.dodgei            .R......A...D.Y...........S............................D...V
1910-N.lineolata         .R......A...D.Y...........S............................D...V
M33876-N.crassaA         ............................................................
```

```
                                                205
                                  190       200  *      210       220       230       240
                         ....|....|....|....|....|....|....|....|....|....|....|....|
2489-N.crassaA           VAGLWNSEHPAAKARFRELAEMEVHRHRAENPHLYPDQPRFPTTDPVPPRMRYPCVISPE
8900-N.crassaA           ............................................................
8830-N.crassaB           ...................................................K........
8858-N.crassaC           ........................Q...................................
8838-PS3                 ............................................................
8770-N.sitophila         ............................................................
8774-N.tetrasperma       ........................Q...................................
8817-PS1                 ............................................................
8901-N.intermediaA       ........................Q..........................I........
8844-N.intermediaB       ........................Q...................................
8847-PS2                 ............................................................
8780-N.discreta          ................M.......Q...................................
958-G.calospora          ................I.......A.............S................E....
5508-N.sublineolata      ................I.......A.............S................E.L..
959-G.cerealis           ................M.......R..............................E....
7221-N.pannonica         ................M.......R........F.....................E....
1740-N.africana          ................M.......R.............S...............YE.L..
1739-N.galapagosensis    ................M.......R.............S...............YE....
1692-N.dodgei            ................M.......R.............S.........L......E....
1910-N.lineolata         ................M.......R.........*
M33876-N.crassaA         ............................................................
```

Supplemental Figure. Amino acid alignment of *mat A-3*

```
                                250       260       270       280       290       300
                       ....|....|....|....|....|....|....|....|....|....|....|....|
2489-N.crassaA         DRQRILRMLDFVWEESNGQLAAEEAALNDVVQPQQAEEVGPFPDFEWEEPNHIIDMSTDL
8900-N.crassaA         ............................................................
8830-N.crassaB         ............................................................
8858-N.crassaC         ...................................V........................
8838-PS3               ............................................................
8770-N.sitophila       ..............................F.............................
8774-N.tetrasperma     ............................................................
8817-PS1               ............................................................
8901-N.intermediaA     ............................................................
8844-N.intermediaB     ............................................................
8847-PS2               ............................................................
8780-N.discreta        ..........................V..TE.T.E....D....Y......Q........
958-G.calospora        ..........................V..A.........A...........QV.......
5508-N.sublineolata    ..........................V..A.........A...........Q........
959-G.cerealis         ..........................V..A..............Y......Q........
7221-N.pannonica       ..........................V..A..............Y......Q........
1740-N.africana        ..........................V..A........I.....Y.....TQ........
1739-N.galapagosensis  ..........................V..A........I.....Y.....TQ........
1692-N.dodgei          ..........................V..A........I.....Y......Q........
1910-N.lineolata
M33876-N.crassaA       ............................................................


                                310       320
                       ....|....|....|....|....|
2489-N.crassaA         SVAQDPDFMMTEDDSMRFLLKQAS*
8900-N.crassaA         .......................C*
8830-N.crassaB         .......................C*
8858-N.crassaC         .......................C*
8838-PS3               .......................C*
8770-N.sitophila       .......................C*
8774-N.tetrasperma     ...................FR..C*
8817-PS1               .......................C*
8901-N.intermediaA     ................Q......C*
8844-N.intermediaB     .......................C*
8847-PS2               .......................C*
8780-N.discreta        ................G......C*
958-G.calospora        ................E...T..C*
5508-N.sublineolata    ................E......C*
959-G.cerealis         ........I.......G......C*
7221-N.pannonica       ................G......C*
1740-N.africana        .........I......G......C*
1739-N.galapagosensis  .........I......G......C*
1692-N.dodgei          ................G......C*
1910-N.lineolata
M33876-N.crassaA       ........................*
```

Supplemental Figure. Nucleotide alignment of a part of the coding region of *act*

```
                                 10        20        30        40        50        60
                        ....|....|....|....|....|....|....|....|....|....|....|....|
8900-N.crassaA          ATCCTCACTCTCAGGTACCCCATCGAGCACGGTGTCGTTACCAACTGGGACGACATGGAG
8772-N.crassaB          ............................................................
8860-N.crassaC          ............................................................
8835-PS3                ..................................................T.........
412-N.sitophila         ............................................................
8775-N.tetrasperma      .......................T....................................
8817-PS1                ............................................................
8901-N.intermediaA      ............................................................
8844-N.intermediaB      ............................................................
8853-PS2                ............................................................
8827-N.discreta         ............................................................
958-G.calospora         ........C...................................................
5508-N.sublineolata     ........C...................................................
959-G.cerealis          ............................................................
7221-N.pannonica        ............................................................
1889-N.terricola        .....T......................................................
1740-N.africana         ....................T.......................................
1739-N.galapagosensis   ....................T.......................................
1692-N.dodgei           ....................T.......................................
1910-N.lineolata        ....................T.......................................
```

```
                                 70        80        90       100       110       120
                        ....|....|....|....|....|....|....|....|....|....|....|....|
8900-N.crassaA          AAGATTTGGCATCACACCTTCTACAACGAGCTCCGTGTCGCCCCCGAGGAGCACCCCGTC
8772-N.crassaB          .........................................T..................
8860-N.crassaC          ............................................................
8835-PS3                ............................................................
412-N.sitophila         ............................................................
8775-N.tetrasperma      ............................................................
8817-PS1                ............................................................
8901-N.intermediaA      ............................................................
8844-N.intermediaB      ............................................................
8853-PS2                ............................................................
8827-N.discreta         ............................................................
958-G.calospora         ......................................T.....................
5508-N.sublineolata     ......................................T.....................
959-G.cerealis          ............................................................
7221-N.pannonica        ............................................................
1889-N.terricola        ............................................................
1740-N.africana         ............................................................
1739-N.galapagosensis   ............................................................
1692-N.dodgei           ............................................................
1910-N.lineolata        ............................................................
```

Supplemental Figure. Nucleotide alignment of a part of the coding region of *act*

```
                               130       140       150       160       170       180
                       ....|....|....|....|....|....|....|....|....|....|....|....|
8900-N.crassaA         CTTCTTACCGAGGCTCCCATCAACCCCAAGTCCAACCGTGAGAAGATGACACAGATCGTT
8772-N.crassaB         ............................................................
8860-N.crassaC         ............................................................
8835-PS3               ............................................................
412-N.sitophila        ............................................................
8775-N.tetrasperma     ............................................................
8817-PS1               ............................................................
8901-N.intermediaA     ............................................................
8844-N.intermediaB     ............................................................
8853-PS2               ............................................................
8827-N.discreta        .................T..........................................
958-G.calospora        ..C..............T..........................................
5508-N.sublineolata    ..C..............T..........................................
959-G.cerealis         ..C..............T..........................................
7221-N.pannonica       ..C..............T......................................T...
1889-N.terricola       ..C..............T..........................................
1740-N.africana        ..C..............T..........................................
1739-N.galapagosensis  ..C..............T..........................................
1692-N.dodgei          ..C..............T..........................................
1910-N.lineolata       ..C..............T..........................................
```

```
                               190       200       210       220       230       240
                       ....|....|....|....|....|....|....|....|....|....|....|....|
8900-N.crassaA         TTCGAGACTTTCAACGCCCCCGCCTTCTACGTCTCCATCCAGGCCGTCCTTTCTCTCTAC
8772-N.crassaB         ............................................................
8860-N.crassaC         ............................................................
8835-PS3               ............................................................
412-N.sitophila        ............................................................
8775-N.tetrasperma     ............................................................
8817-PS1               ............................................................
8901-N.intermediaA     .....................................................C......
8844-N.intermediaB     ........................................................T...
8853-PS2               ...........T................................................
8827-N.discreta        ............................................................
958-G.calospora        ........G..........................T........................
5508-N.sublineolata    ........G..........................T...........T............
959-G.cerealis         ........C...................................................
7221-N.pannonica       ........G...................................................
1889-N.terricola       ........G...................................................
1740-N.africana        ........C...................................................
1739-N.galapagosensis  ........C...................................................
1692-N.dodgei          ........C...................................................
1910-N.lineolata       ........C...................................................
```

Supplemental Figure. Nucleotide alignment of a part of the coding region of *act*

```
                                  250       260       270       280       290       300
                         ....|....|....|....|....|....|....|....|....|....|....|....|
8900-N.crassaA           GCCTCCGGTCGTACCACCGGTATCGTCCTCGACTCCGGTGACGGTGTCACTCACGTCGTC
8772-N.crassaB           ............................................................
8860-N.crassaC           ............................................................
8835-PS3                 ................................T...........................
412-N.sitophila          ..........................T.................................
8775-N.tetrasperma       ............................................................
8817-PS1                 ............................................................
8901-N.intermediaA       ............................................................
8844-N.intermediaB       ............................................................
8853-PS2                 ............................................................
8827-N.discreta          ..........................T..T...........T..................
958-G.calospora          .........................................T..................
5508-N.sublineolata      .........................................T..................
959-G.cerealis           .........................................T..................
7221-N.pannonica         .........................................T..................
1889-N.terricola         .........................................T..................
1740-N.africana          ..........................T..............T..................
1739-N.galapagosensis    ..........................T..............T..................
1692-N.dodgei            ..........................T..............T..................
1910-N.lineolata         ..........................T..............T..................
```

```
                                  310       320       330       340       350       360
                         ....|....|....|....|....|....|....|....|....|....|....|....|
8900-N.crassaA           CCCATCTACGAGGGTTTCGCCCTTCCCCACGCCATTGCCCGTGTTGACATGGCTGGCCGT
8772-N.crassaB           .......................C....................................
8860-N.crassaC           ............................................................
8835-PS3                 ............................................................
412-N.sitophila          ............................................................
8775-N.tetrasperma       ............................................................
8817-PS1                 ............................................................
8901-N.intermediaA       ..........................T.................................
8844-N.intermediaB       ............................................................
8853-PS2                 ............................................................
8827-N.discreta          ............................................................
958-G.calospora          ............................................................
5508-N.sublineolata      ............................................................
959-G.cerealis           ............................................................
7221-N.pannonica         ............................................................
1889-N.terricola         ............................................................
1740-N.africana          ............................................................
1739-N.galapagosensis    ............................................................
1692-N.dodgei            ............................................................
1910-N.lineolata         ............................................................
```

Supplemental Figure. Nucleotide alignment of a part of the coding region of *act*

```
                                   370       380       390       400       410       420
                          ....|....|....|....|....|....|....|....|....|....|....|....|
8900-N.crassaA            GATCTTACCGACTACCTCATGAAGATTCTCGCTGAGCGTGGTTACACCTTCTCCACCACC
8772-N.crassaB            ............................................................
8860-N.crassaC            ............................................................
8835-PS3                  ............................................................
412-N.sitophila           ............................................................
8775-N.tetrasperma        ........T...................................................
8817-PS1                  ............................................................
8901-N.intermediaA        ............................................................
8844-N.intermediaB        ............................................................
8853-PS2                  ............................................................
8827-N.discreta           ..........................C.................................
958-G.calospora           ..........................C..............C..T...............
5508-N.sublineolata       ..........................C..............C..................
959-G.cerealis            ..........................C..............C..................
7221-N.pannonica          .........................................C..................
1889-N.terricola          .........................................C..................
1740-N.africana           ..........................C..............C..................
1739-N.galapagosensis     ..........................C..............C..................
1692-N.dodgei             ..........................C..............C..................
1910-N.lineolata          ..........................C..............C..................
```

```
                                   430       440       450       460       470       480
                          ....|....|....|....|....|....|....|....|....|....|....|....|
8900-N.crassaA            GCCGAGCGCGAAATCGTTCGTGACATCAAGGAGAAGCTCTGCTACGTCGCTCTCGACTTC
8772-N.crassaB            ............................................................
8860-N.crassaC            ............................................................
8835-PS3                  ............................................................
412-N.sitophila           ............................................................
8775-N.tetrasperma        ............................................................
8817-PS1                  ............................................................
8901-N.intermediaA        ............................................................
8844-N.intermediaB        ............................................................
8853-PS2                  ............................................................
8827-N.discreta           ............................................................
958-G.calospora           ............................................................
5508-N.sublineolata       ............................................................
959-G.cerealis            ............................................................
7221-N.pannonica          ............................................................
1889-N.terricola          ............................................................
1740-N.africana           ............................................................
1739-N.galapagosensis     ..T.........................................................
1692-N.dodgei             ............................................................
1910-N.lineolata          ............................................................
```

Supplemental Figure. Nucleotide alignment of a part of the coding region of *act*

```
                                490       500       510       520       530       540
                       ....|....|....|....|....|....|....|....|....|....|....|....|
8900-N.crassaA         GAGCAGGAGATTCAGACCGCTGCCCAGAGCTCTTCTCTCGAGAAGTCCTACGAGCTTCCC
8772-N.crassaB         ...............................................T............
8860-N.crassaC         ............................................................
8835-PS3               ...................................C........................
412-N.sitophila        ............................................................
8775-N.tetrasperma     ............................................................
8817-PS1               ............................................................
8901-N.intermediaA     ...................................C........................
8844-N.intermediaB     ...........C.......................C..T.....................
8853-PS2               ...................................C........................
8827-N.discreta        ...................................C........................
958-G.calospora        ...........C........C..............C........................
5508-N.sublineolata    ...........C........C..T...........C........................
959-G.cerealis         ...........C.......................C........................
7221-N.pannonica       ...........C........C..............C........................
1889-N.terricola       ...........C.......................C.......................T
1740-N.africana        ...........C.......................C........................
1739-N.galapagosensis  ...........C.......................C........................
1692-N.dodgei          ...........C.......................C........................
1910-N.lineolata       ...........C.......................C........................
```

```
                                550       560       570       580       590       600
                       ....|....|....|....|....|....|....|....|....|....|....|....|
8900-N.crassaA         GACGGTCAGGTCATCACCATCGGCAACGAGCGTTTCCGTGCCCCTGAGGCTCTCTTCCAG
8772-N.crassaB         ............................................................
8860-N.crassaC         .....................................................T......
8835-PS3               .....................................................T......
412-N.sitophila        ............................................................
8775-N.tetrasperma     ............................................................
8817-PS1               ............................................................
8901-N.intermediaA     ............................................................
8844-N.intermediaB     ............................................................
8853-PS2               ............................................................
8827-N.discreta        ............................................................
958-G.calospora        ................................C........T..................
5508-N.sublineolata    ................................C........T..................
959-G.cerealis         .........................................T..................
7221-N.pannonica       .........................................T..................
1889-N.terricola       .........................................T..................
1740-N.africana        .........................................T..................
1739-N.galapagosensis  .........................................T..................
1692-N.dodgei          .........................................T..................
1910-N.lineolata       .........................................T..................
```

Supplemental Figure. Nucleotide alignment of a part of the coding region of *act*

```
                               610       620       630       640       650       660
                       ....|....|....|....|....|....|....|....|....|....|....|....|
8900-N.crassaA         CCTTCCGTCCTTGGTCTCGAATCTGGCGGCATCCACGTCACCACTTTCAACTCCATCATG
8772-N.crassaB         .............................T..............................
8860-N.crassaC         .............................T..............................
8835-PS3               .............................T..............................
412-N.sitophila        .............................T..............................
8775-N.tetrasperma     .............................T..............................
8817-PS1               .............................T..............................
8901-N.intermediaA     .............................T..............................
8844-N.intermediaB     .............................T..............................
8853-PS2               .............................T..............................
8827-N.discreta        .............................T..............C...............
958-G.calospora        .............................T..............................
5508-N.sublineolata    .............................T..............................
959-G.cerealis         .............................T..............................
7221-N.pannonica       .............................T..............................
1889-N.terricola       .............................T..............................
1740-N.africana        .............................T..............C...............
1739-N.galapagosensis  .............................T..............C...............
1692-N.dodgei          .............................T..............C...............
1910-N.lineolata       .............................T..............C...............

                                                                    Intron
                                                                       |
                               670       680       690       700       v710       720
                       ....|....|....|....|....|....|....|....|....|....|....|....|
8900-N.crassaA         AAGTGCGATGTCGACGTCAGGAAGGATCTCTATGGCAACATTGTCATGTCTGGTGGTACC
8772-N.crassaB         ............................................................
8860-N.crassaC         ............................................................
8835-PS3               ............................................................
412-N.sitophila        ............................................................
8775-N.tetrasperma     ............................................................
8817-PS1               ............................................................
8901-N.intermediaA     ............................................................
8844-N.intermediaB     ............................................................
8853-PS2               ............................................................
8827-N.discreta        ............................................................
958-G.calospora        ................................C...........................
5508-N.sublineolata    ................................C...........................
959-G.cerealis         ............................................................
7221-N.pannonica       ..............T.............................................
1889-N.terricola       ............................................................
1740-N.africana        ..............T.............................................
1739-N.galapagosensis  ..............T.............................................
1692-N.dodgei          ..............T.............................................
1910-N.lineolata       ..............T.............................................
```

Supplemental Figure. Nucleotide alignment of a part of the coding region of *act*

```
                                730       740       750       760
                       ....|....|....|....|....|....|....|....|....|..
8900-N.crassaA         ACCATGTACCCTGGTCTCTCCGACCGTATGCAGAAGGAGATCACTGC
8772-N.crassaB         ...............................................
8860-N.crassaC         ...............................................
8835-PS3               ...............................................
412-N.sitophila        ...............................................
8775-N.tetrasperma     ...............................................
8817-PS1               ...............................................
8901-N.intermediaA     ...........C...................................
8844-N.intermediaB     ...............................................
8853-PS2               ...............................................
8827-N.discreta        ..T............................................
958-G.calospora        ....-------------------------------------------
5508-N.sublineolata    ...............................................
959-G.cerealis         ...............................................
7221-N.pannonica       ...............................................
1889-N.terricola       ...............................................
1740-N.africana        ...............................................
1739-N.galapagosensis  ...............................................
1692-N.dodgei          ...............................................
1910-N.lineolata       ...............................................
```

Supplemental Figure. Amino acid alignment of a part of ACTIN

```
                                10        20        30        40        50        60
                       ....|....|....|....|....|....|....|....|....|....|....|....|
8900-N.crassaA         ILTLRYPIEHGVVTNWDDMEKIWHHTFYNELRVAPEEHPVLLTEAPINPKSNREKMTQIV
8772-N.crassaB         ............................................................
8860-N.crassaC         ............................................................
8835-PS3               ............................................................
412-N.sitophila        ............................................................
8775-N.tetrasperma     ............................................................
8817-PS1               ............................................................
8901-N.intermediaA     ............................................................
8844-N.intermediaB     ............................................................
8853-PS2               ............................................................
8827-N.discreta        ............................................................
958-G.calospora        ............................................................
5508-N.sublineolata    ............................................................
959-G.cerealis         ............................................................
7221-N.pannonica       ............................................................
1889-N.terricola       ............................................................
1740-N.africana        ............................................................
1739-N.galapagosensis  ............................................................
1692-N.dodgei          ............................................................
1910-N.lineolata       ............................................................
```

```
                                70        80        90       100       110       120
                       ....|....|....|....|....|....|....|....|....|....|....|....|
8900-N.crassaA         FETFNAPAFYVSIQAVLSLYASGRTTGIVLDSGDGVTHVVPIYEGFALPHAIARVDMAGR
8772-N.crassaB         ............................................................
8860-N.crassaC         ............................................................
8835-PS3               ............................................................
412-N.sitophila        ............................................................
8775-N.tetrasperma     ............................................................
8817-PS1               ............................................................
8901-N.intermediaA     ............................................................
8844-N.intermediaB     ............................................................
8853-PS2               ............................................................
8827-N.discreta        ............................................................
958-G.calospora        ............................................................
5508-N.sublineolata    ............................................................
959-G.cerealis         ............................................................
7221-N.pannonica       ............................................................
1889-N.terricola       ............................................................
1740-N.africana        ............................................................
1739-N.galapagosensis  ............................................................
1692-N.dodgei          ............................................................
1910-N.lineolata       ............................................................
```

Supplemental Figure. Amino acid alignment of a part of ACTIN

```
                                130       140       150       160       170       180
                         ....|....|....|....|....|....|....|....|....|....|....|....|
8900-N.crassaA           DLTDYLMKILAERGYTFSTTAEREIVRDIKEKLCYVALDFEQEIQTAAQSSSLEKSYELP
8772-N.crassaB           ............................................................
8860-N.crassaC           ............................................................
8835-PS3                 ............................................................
412-N.sitophila          ............................................................
8775-N.tetrasperma       ............................................................
8817-PS1                 ............................................................
8901-N.intermediaA       ............................................................
8844-N.intermediaB       ............................................................
8853-PS2                 ............................................................
8827-N.discreta          ............................................................
958-G.calospora          ............................................................
5508-N.sublineolata      ............................................................
959-G.cerealis           ............................................................
7221-N.pannonica         ............................................................
1889-N.terricola         ............................................................
1740-N.africana          ............................................................
1739-N.galapagosensis    ............................................................
1692-N.dodgei            ............................................................
1910-N.lineolata         ............................................................
```

```
                                                                               Intron
                                190       200       210       220       230      ↓ 240
                         ....|....|....|....|....|....|....|....|....|....|....|....|
8900-N.crassaA           DGQVITIGNERFRAPEALFQPSVLGLESGGIHVTTFNSIMKCDVDVRKDLYGNIVMSGGT
8772-N.crassaB           ............................................................
8860-N.crassaC           ............................................................
8835-PS3                 ............................................................
412-N.sitophila          ............................................................
8775-N.tetrasperma       ............................................................
8817-PS1                 ............................................................
8901-N.intermediaA       ............................................................
8844-N.intermediaB       ............................................................
8853-PS2                 ............................................................
8827-N.discreta          ............................................................
958-G.calospora          ............................................................
5508-N.sublineolata      ............................................................
959-G.cerealis           ............................................................
7221-N.pannonica         ............................................................
1889-N.terricola         ............................................................
1740-N.africana          ............................................................
1739-N.galapagosensis    ............................................................
1692-N.dodgei            ............................................................
1910-N.lineolata         ............................................................
```

Supplemental Figure. Amino acid alignment of a part of ACTIN

```
                                250
                        ....|....|....|
8900-N.crassaA          TMYPGLSDRMQKEIT
8772-N.crassaB          ...............
8860-N.crassaC          ...............
8835-PS3                ...............
412-N.sitophila         ...............
8775-N.tetrasperma      ...............
8817-PS1                ...............
8901-N.intermediaA      ...............
8844-N.intermediaB      ...............
8853-PS2                ...............
8827-N.discreta         ...............
958-G.calospora         .--------------
5508-N.sublineolata     ...............
959-G.cerealis          ...............
7221-N.pannonica        ...............
1889-N.terricola        ...............
1740-N.africana         ...............
1739-N.galapagosensis   ...............
1692-N.dodgei           ...............
1910-N.lineolata        ...............
```

Supplemental Figure. Nucleotide alignment of a part of the coding region of *tef-1*

```
                                  10        20        30        40        50        60
                         ....|....|....|....|....|....|....|....|....|....|....|....|
8900-N.crassaA           ATTGCCGCCGGTACTGGTGAGTTCGAGGCTGGTATCTCCAAGGATGGCCAGACCCGTGAG
8772-N.crassaB           ............................................................
8860-N.crassaC           ............................................................
8835-PS3                 ............................................................
412-N.sitophila          ............................................................
8775-N.tetrasperma       ........T......................................T............
8817-PS1                 ............................................................
8901-N.intermedia        ............................................................
8844-N.intermedia        ............................................................
8853-PS2                 ............................................................
8827-N.discreta          ........T...................................................
958-G.calospora          ........T...................................................
5508-N.sublineolata      ---------...................................................
959-G.cerealis           ........T...................................................
7221-N.pannonica         ........T...................................................
1889-N.terricola         ---------------------.......................................
1740-N.africana          ........T...................................................
1739-N.galapagosensis    ........T...................................................
1692-N.dodgei            ........T...................................................
1910-N.lineolata         ........T...................................................

                                  70        80        90       100       110       120
                         ....|....|....|....|....|....|....|....|....|....|....|....|
8900-N.crassaA           CACGCTCTTCTCGCCTACACCCTCGGTGTCAAGCAGCTCATTGTTGCCATCAACAAGATG
8772-N.crassaB           ........G...................................................
8860-N.crassaC           ........G...................................................
8835-PS3                 ........G...................................................
412-N.sitophila          ........G...................................................
8775-N.tetrasperma       ........G................................C..................
8817-PS1                 ........G.............................T.....................
8901-N.intermedia        ........G................................C..................
8844-N.intermedia        ........G..............T....................................
8853-PS2                 ........G..............T....................................
8827-N.discreta          ........G................................C..................
958-G.calospora          ........G................................C..................
5508-N.sublineolata      ........G................................C..................
959-G.cerealis           ........G................................C..................
7221-N.pannonica         ........G................................C..................
1889-N.terricola         ........G................................C..................
1740-N.africana          ........G................................C..................
1739-N.galapagosensis    ........G................................C..................
1692-N.dodgei            ........G...................................................
1910-N.lineolata         ........G................................C..................
```

Supplemental Figure. Nucleotide alignment of a part of the coding region of *tef-1*

```
                              130       140       150       160       170       180
                      ....|....|....|....|....|....|....|....|....|....|....|....|
8900-N.crassaA        GACACCACCCAGTGGTCCCAGACTCGTTTCGAGGAGATCATCAAGGAGACCAAGAACTTC
8772-N.crassaB        ............................................................
8860-N.crassaC        ............................................................
8835-PS3              ............................................................
412-N.sitophila       .................T..........................................
8775-N.tetrasperma    ............................................................
8817-PS1              .......................C....................................
8901-N.intermedia     ............................................................
8844-N.intermedia     ............................................................
8853-PS2              ............................................................
8827-N.discreta       ............................................................
958-G.calospora       .....................T......................................
5508-N.sublineolata   .....................T......................................
959-G.cerealis        .....................T................T.....................
7221-N.pannonica      .....................T......................................
1889-N.terricola      .....................T......................................
1740-N.africana       ............................................................
1739-N.galapagosensis ............................................................
1692-N.dodgei         ............................................................
1910-N.lineolata      ............................................................
```

```
                              190       200       210       220       230       240
                      ....|....|....|....|....|....|....|....|....|....|....|....|
8900-N.crassaA        ATCAAGAAGGTTGGCTACAACCCCGCTGGTGTCGCTTTCGTCCCCATCTCCGGCTTCAAC
8772-N.crassaB        ............................................................
8860-N.crassaC        ............................................................
8835-PS3              ............................................................
412-N.sitophila       ............................................................
8775-N.tetrasperma    ............................................................
8817-PS1              ............................................................
8901-N.intermedia     ............................................................
8844-N.intermedia     ............................................................
8853-PS2              ............................................................
8827-N.discreta       ................................T...........................
958-G.calospora       ...........C...............TCC..............................
5508-N.sublineolata   ...........C...............TCC..............................
959-G.cerealis        ...........................AC...............................
7221-N.pannonica      ...........C...............A................................
1889-N.terricola      ...........................A................................
1740-N.africana       ................................T...........................
1739-N.galapagosensis ................................T...........................
1692-N.dodgei         ................................T...........................
1910-N.lineolata      ................................T...........................
```

Supplemental Figure. Nucleotide alignment of a part of the coding region of *tef-1*

```
                               250       260       270       280       290       300
                       ....|....|....|....|....|....|....|....|....|....|....|....|
8900-N.crassaA         GGCGACAACATGCTTGAGCCCTCCACCAACTGCCCCTGGTACAAGGGTTGGGAGAAGGAG
8772-N.crassaB         ............................................................
8860-N.crassaC         ............................................................
8835-PS3               ............................................................
412-N.sitophila        ...............................................C............
8775-N.tetrasperma     ............................................................
8817-PS1               ............................................................
8901-N.intermedia      ............................................................
8844-N.intermedia      ...............................................C............
8853-PS2               ............................................................
8827-N.discreta        ........................T......................C............
958-G.calospora        ....................T.......................................
5508-N.sublineolata    ....................T....G..................................
959-G.cerealis         ........................T...................................
7221-N.pannonica       ........................G...................................
1889-N.terricola       ........................C...................................
1740-N.africana        ............................................................
1739-N.galapagosensis  ............................................................
1692-N.dodgei          ............................................................
1910-N.lineolata       ............................................................
```

```
                               310       320       330       340       350       360
                       ....|....|....|....|....|....|....|....|....|....|....|....|
8900-N.crassaA         ACCAAGGCCGGCAAGGCCACTGGCAAGACCCTCCTCGAGGCCATCGACGCCATTGAGCCC
8772-N.crassaB         ............................................................
8860-N.crassaC         ............................................................
8835-PS3               ............................................................
412-N.sitophila        ...........T......T.............T...........................
8775-N.tetrasperma     ............................................................
8817-PS1               ............................................................
8901-N.intermedia      ........T...................................................
8844-N.intermedia      ................................T...........T...............
8853-PS2               ................................T...........T...............
8827-N.discreta        ............................................................
958-G.calospora        ...............T............................................
5508-N.sublineolata    ...............T............................................
959-G.cerealis         ...............A............................................
7221-N.pannonica       ...............T...................T........................
1889-N.terricola       ...............T............................................
1740-N.africana        ............................................................
1739-N.galapagosensis  ............................................................
1692-N.dodgei          ............................................................
1910-N.lineolata       ............................................................
```

Supplemental Figure. Nucleotide alignment of a part of the coding region of *tef-1*

```
                              370       380       390       400       410       420
                     ....|....|....|....|....|....|....|....|....|....|....|....|
8900-N.crassaA       CCCAAGCGTCCTACCGACAAGCCCCTCCGTCTTCCCCTCCAGGATGTCTACAAGATCGGT
8772-N.crassaB       ............................................................
8860-N.crassaC       ............................................................
8835-PS3             ............................................................
412-N.sitophila      ............................................................
8775-N.tetrasperma   ...............................................T............
8817-PS1             ............................................................
8901-N.intermedia    ..........................T.................................
8844-N.intermedia    ............................................................
8853-PS2             ............................................................
8827-N.discreta      ...........C................................................
958-G.calospora      ...........C................................................
5508-N.sublineolata  ..T........C................................................
959-G.cerealis       ...........C...............................................C
7221-N.pannonica     ...........C..........................T....................C
1889-N.terricola     ...........C..........................T....................C
1740-N.africana      ............................................................
1739-N.galapagosensis ............................................................
1692-N.dodgei        ............................................................
1910-N.lineolata     ............................................................
```

```
                              430       440       450       460       470       480
                     ....|....|....|....|....|....|....|....|....|....|....|....|
8900-N.crassaA       GGTATCGGCACAGTGCCCGTCGGCCGTATCGAGACTGGTGTCCTCAAGCCCGGTATGGTC
8772-N.crassaB       ............................................T...............
8860-N.crassaC       ............................................................
8835-PS3             ............................................................
412-N.sitophila      ............................................................
8775-N.tetrasperma   .......................................A.T..................
8817-PS1             ............................................................
8901-N.intermedia    ............................................................
8844-N.intermedia    .....T......................................................
8853-PS2             .....T...............................................C......
8827-N.discreta      ............................................................
958-G.calospora      ............................................................
5508-N.sublineolata  ..................................................T.........
959-G.cerealis       .......................................A....................
7221-N.pannonica     ............................................................
1889-N.terricola     .......................................A....................
1740-N.africana      ............................................................
1739-N.galapagosensis ............................................................
1692-N.dodgei        ............................................................
1910-N.lineolata     ............................................................
```

Supplemental Figure. Nucleotide alignment of a part of the coding region of *tef-1*

```
                                 490       500       510       520       530       540
                        ....|....|....|....|....|....|....|....|....|....|....|....|
8900-N.crassaA          GTTACCTTCGCTCCTTCCAACGTCACCACTGAAGTCAAGTCCGTCGAGATGCACCACGAG
8772-N.crassaB          ............................................................
8860-N.crassaC          ............................................................
8835-PS3                ............................................................
412-N.sitophila         .......................T....................................
8775-N.tetrasperma      ............................................................
8817-PS1                .......................T....................................
8901-N.intermedia       ............................................................
8844-N.intermedia       ............................................................
8853-PS2                ............................................................
8827-N.discreta         .......................T.............................T......
958-G.calospora         ............................................................
5508-N.sublineolata     ............................................................
959-G.cerealis          ............................................................
7221-N.pannonica        ............................................................
1889-N.terricola        ............................................................
1740-N.africana         ............................................................
1739-N.galapagosensis   ............................................................
1692-N.dodgei           ............................................................
1910-N.lineolata        ............................................................
```

```
                                 550       560       570       580       590       600
                        ....|....|....|....|....|....|....|....|....|....|....|....|
8900-N.crassaA          CAGCTTGCTCAGGGTGTCCCCGGTGACAACGTCGGCTTCAACGTGAAGAACGTTTCCGTC
8772-N.crassaB          ................................T...........................
8860-N.crassaC          ............................................................
8835-PS3                ............................................................
412-N.sitophila         ............................................................
8775-N.tetrasperma      ............................................................
8817-PS1                .....C......................................................
8901-N.intermedia       ............................................................
8844-N.intermedia       ............................................................
8853-PS2                ............................................................
8827-N.discreta         ............................................................
958-G.calospora         .....................................................C......
5508-N.sublineolata     .....................................................C......
959-G.cerealis          ...................................T........................
7221-N.pannonica        ............................................................
1889-N.terricola        ......A........A.G..........................................
1740-N.africana         .................T......................................T...
1739-N.galapagosensis   .................T..........................................
1692-N.dodgei           .................T..........................................
1910-N.lineolata        .................T..........................................
```

Supplemental Figure. Nucleotide alignment of a part of the coding region of *tef-1*

```
                                 610       620       630       640       650       660
                        ....|....|....|....|....|....|....|....|....|....|....|....|
8900-N.crassaA          AAGGATATCCGCCGTGGTAACGTTGCCGGTGACTCCAAGAACGACCCCCCTGCTGGCGCC
8772-N.crassaB          ............................................................
8860-N.crassaC          ............................................................
8835-PS3                ............................................................
412-N.sitophila         ............................................................
8775-N.tetrasperma      ............................................................
8817-PS1                ............................................................
8901-N.intermedia       ...........................................................T
8844-N.intermedia       ...........................................................T
8853-PS2                ............................................................
8827-N.discreta         ............................................................
958-G.calospora         ....................................................T......T
5508-N.sublineolata     ............................................T.......T......T
959-G.cerealis          ....................................................T......T
7221-N.pannonica        ....................................................T......T
1889-N.terricola        ....................................................T......T
1740-N.africana         ...........................................................T
1739-N.galapagosensis   ...........................................................T
1692-N.dodgei           ...........................................................T
1910-N.lineolata        ...........................................................T
```

```
                                 670       680       690       700       710       720
                        ....|....|....|....|....|....|....|....|....|....|....|....|
8900-N.crassaA          GCCTCTTTCACCGCCCAGGTCATCGTTCTCAACCACCCCGGTCAGGTCGGTGCCGGCTAC
8772-N.crassaB          ......................................T.....................
8860-N.crassaC          ..T.................T.......................................
8835-PS3                ............................................................
412-N.sitophila         ....................T.......................................
8775-N.tetrasperma      ....................T.......................................
8817-PS1                ..T.................T.......................................
8901-N.intermedia       ....................T.......................................
8844-N.intermedia       ....................T.......................................
8853-PS2                ..............T.....T.......................................
8827-N.discreta         ....................T.......................................
958-G.calospora         ....................T.....C..T...........C..................
5508-N.sublineolata     ....................T.....C..T...........C..................
959-G.cerealis          ..............T.....T.....C..T...........C..................
7221-N.pannonica        ....................T.....C..T...........C..................
1889-N.terricola        ....................T.....C..T...........C..................
1740-N.africana         ...........T........T.....C..T..............................
1739-N.galapagosensis   ...........T........T.....C..T..............................
1692-N.dodgei           ...........T........T.....C..T..............................
1910-N.lineolata        ...........T........T.....C..T..............................
```

Supplemental Figure. Nucleotide alignment of a part of the coding region of *tef-1*

```
                                730       740       750       760       770       780
                         ....|....|....|....|....|....|....|....|....|....|....|....|
8900-N.crassaA           GCCCCCGTCCTCGACTGCCACACTGCCCACATTGCCTGCAAGTTCGCCGAGCTCCTCGAG
8772-N.crassaB           ............................................................
8860-N.crassaC           ............................................................
8835-PS3                 ............................................................
412-N.sitophila          .......................C....................................
8775-N.tetrasperma       ............................................................
8817-PS1                 ............................................................
8901-N.intermedia        ............................................................
8844-N.intermedia        ............................................................
8853-PS2                 ............................................................
8827-N.discreta          ............................................................
958-G.calospora          ..............T.............................................
5508-N.sublineolata      ..............T.............................................
959-G.cerealis           ..............T.............................................
7221-N.pannonica         ............................................................
1889-N.terricola         ............................................................
1740-N.africana          ...........T..T.............................................
1739-N.galapagosensis    ...........T..T.............................................
1692-N.dodgei            ...........T..T.............................................
1910-N.lineolata         ...........T..T.............................................
```

```
                                790       800       810       820       830
                         ....|....|....|....|....|....|....|....|....|....|....
8900-N.crassaA           AAGATCGACCGCCGTACTGGTAAGGCTGTTGAGGCCTCCCCCAAGTTCATCAAG
8772-N.crassaB           ......................................................
8860-N.crassaC           ......................................................
8835-PS3                 ......................................................
412-N.sitophila          ......................................................
8775-N.tetrasperma       .............................C........................
8817-PS1                 ......................................................
8901-N.intermedia        ......................................................
8844-N.intermedia        ......................................................
8853-PS2                 ........T..............................---------------
8827-N.discreta          ........T.............................T...............
958-G.calospora          ........T.............................................
5508-N.sublineolata      ........T.............................................
959-G.cerealis           ........T.............................................
7221-N.pannonica         ........T.............................................
1889-N.terricola         ........T.............................................
1740-N.africana          ........T.............................T...............
1739-N.galapagosensis    ........T.............................T...............
1692-N.dodgei            ........T.............................T...............
1910-N.lineolata         ........T.............................T...............
```

Supplemental Figure. Amino acid alignment of a part of EF-1α

```
                                10        20        30        40        50        60
                       ....|....|....|....|....|....|....|....|....|....|....|....|
8900-N.crassaA         IAAGTGEFEAGISKDGQTREHALLAYTLGVKQLIVAINKMDTTQWSQTRFEEIIKETKNF
8772-N.crassaB         ............................................................
8860-N.crassaC         ............................................................
8835-PS3               ............................................................
412-N.sitophila        ............................................................
8775-N.tetrasperma     ............................................................
8817-PS1               ............................................................
8901-N.intermedia      ............................................................
8844-N.intermedia      ............................................................
8853-PS2               ............................................................
8827-N.discreta        ............................................................
958-G.calospora        ...............................................S............
5508-N.sublineolata    ---............................................S............
959-G.cerealis         ...............................................S............
7221-N.pannonica       ...............................................S............
1889-N.terricola       -------........................................S............
1740-N.africana        ............................................................
1739-N.galapagosensis  ............................................................
1692-N.dodgei          ............................................................
1910-N.lineolata       ............................................................
```

```
                                70
                                *
                                70        80      8990       100       110       120
                                                    *
                       ....|....|....|....|....|....|....|....|....|....|....|....|
8900-N.crassaA         IKKVGYNPAGVAFVPISGFNGDNMLEPSTNCPWYKGWEKETKAGKATGKTLLEAIDAIEP
8772-N.crassaB         ............................................................
8860-N.crassaC         ............................................................
8835-PS3               ............................................................
412-N.sitophila        ..............................................S.............
8775-N.tetrasperma     ............................................................
8817-PS1               ............................................................
8901-N.intermedia      ............................................................
8844-N.intermedia      ............................................................
8853-PS2               ............................................................
8827-N.discreta        ............................S...............................
958-G.calospora        .........S...................................S..............
5508-N.sublineolata    .........S..................S................S..............
959-G.cerealis         .........T..................S................T..............
7221-N.pannonica       .........S..................A................S..............
1889-N.terricola       .........S..................P................S..............
1740-N.africana        ............................................................
1739-N.galapagosensis  ............................................................
1692-N.dodgei          ............................................................
1910-N.lineolata       ............................................................
```

Supplemental Figure. Amino acid alignment of a part of EF-1α

```
                                  130       140       150       160       170       180
                         ....|....|....|....|....|....|....|....|....|....|....|....|
8900-N.crassaA           PKRPTDKPLRLPLQDVYKIGGIGTVPVGRIETGVLKPGMVVTFAPSNVTTEVKSVEMHHE
8772-N.crassaB           ............................................................
8860-N.crassaC           ............................................................
8835-PS3                 ............................................................
412-N.sitophila          ............................................................
8775-N.tetrasperma       .................................I..........................
8817-PS1                 ............................................................
8901-N.intermedia        ............................................................
8844-N.intermedia        ............................................................
8853-PS2                 ............................................................
8827-N.discreta          ............................................................
958-G.calospora          ............................................................
5508-N.sublineolata      ............................................................
959-G.cerealis           .................................I..........................
7221-N.pannonica         ............................................................
1889-N.terricola         .................................I..........................
1740-N.africana          ............................................................
1739-N.galapagosensis    ............................................................
1692-N.dodgei            ............................................................
1910-N.lineolata         ............................................................

                                  190       200       210       220       230       240
                         ....|....|....|....|....|....|....|....|....|....|....|....|
8900-N.crassaA           QLAQGVPGDNVGFNVKNVSVKDIRRGNVAGDSKNDPPAGAASFTAQVIVLNHPGQVGAGY
8772-N.crassaB           ............................................................
8860-N.crassaC           ............................................................
8835-PS3                 ............................................................
412-N.sitophila          ............................................................
8775-N.tetrasperma       ............................................................
8817-PS1                 ............................................................
8901-N.intermedia        ............................................................
8844-N.intermedia        ............................................................
8853-PS2                 ............................................................
8827-N.discreta          ............................................................
958-G.calospora          .....................................V......................
5508-N.sublineolata      .....................................V......................
959-G.cerealis           .....................................V......................
7221-N.pannonica         .....................................V......................
1889-N.terricola         ..T..M...............................V......................
1740-N.africana          ............................................................
1739-N.galapagosensis    ............................................................
1692-N.dodgei            ............................................................
1910-N.lineolata         ............................................................
```

Supplemental Figure. Amino acid alignment of a part of EF-1α

```
                               250       260       270
                        ....|....|....|....|....|....|....|...
8900-N.crassaA          APVLDCHTAHIACKFAELLEKIDRRTGKAVEASPKFIK
8772-N.crassaB          ......................................
8860-N.crassaC          ......................................
8835-PS3                ......................................
412-N.sitophila         ......................................
8775-N.tetrasperma      ......................................
8817-PS1                ......................................
8901-N.intermedia       ......................................
8844-N.intermedia       ......................................
8853-PS2                .................................-----
8827-N.discreta         ......................................
958-G.calospora         ......................................
5508-N.sublineolata     ......................................
959-G.cerealis          ......................................
7221-N.pannonica        ......................................
1889-N.terricola        ......................................
1740-N.africana         ......................................
1739-N.galapagosensis   ......................................
1692-N.dodgei           ......................................
1910-N.lineolata        ......................................
```

Supplemental Figure. Nucleotide alignment of a part of the coding region of *ccg-7*

```
                               10        20        30        40        50        60
                       ....|....|....|....|....|....|....|....|....|....|....|....|
8900-N.crassaA         TACACTGAGCGCGACCCCGCTGCCATCCCCTGGTCCGAGACCGGTGCCGACTACATTGTC
8772-N.crassaB         ---------------------------.................................
8860-N.crassaC         ..........................T.................................
8835-PS3               ..........................T.................................
412-N.sitophila        ..........................T.................................
8775-N.tetrasperma     ..........................T.................................
8817-PS1               ..........................T.................................
8901-N.intermediaA     ..........................T.................................
8844-N.intermediaB     ..........................T.................................
8853-PS2               ---------------------------.................................
8827-N.discreta        ............................................................
958-G.calospora        .................................GGT........C...........C...
5508-N.sublineolata    .................................GGT........C...........C...
959-G.cerealis         ...................................T..............T.....C...
7221-N.pannonica       ...................................T..............T.....C...
1889-N.terricola       ..................A..............G.T........C.....T.....C...
1740-N.africana        ...................................T........C.....T.....C...
1739-N.galapagosensis  ...................................T........C.....T.....C...
1692-N.dodgei          ...................................T........C.....T.....C...
1910-N.lineolata       ...................................T........C.....T.....C..T
```

```
                               70        80        90       100       110       120
                       ....|....|....|....|....|....|....|....|....|....|....|....|
8900-N.crassaA         GAGTCCACTGGTGTCTTCACCACCACCGAGAAGGCCTCCGCCCACTTGAAGGGTGGTGCC
8772-N.crassaB         ............................................................
8860-N.crassaC         ............................................................
8835-PS3               ............................................................
412-N.sitophila        ............................................................
8775-N.tetrasperma     ............................................................
8817-PS1               ............................................................
8901-N.intermediaA     ............................................................
8844-N.intermediaB     ............................................................
8853-PS2               ..........................T..............T..................
8827-N.discreta        ............................................................
958-G.calospora        .....T..C....................C..............................
5508-N.sublineolata    ........C....................C..............................
959-G.cerealis         ........C...................................................
7221-N.pannonica       ........C...................................................
1889-N.terricola       ........C...................................................
1740-N.africana        ........C...................................................
1739-N.galapagosensis  ........C...................................................
1692-N.dodgei          ........C...................................................
1910-N.lineolata       ........C....................C..............T...............
```

Supplemental Figure. Nucleotide alignment of a part of the coding region of *ccg-7*

```
                                  130       140       150       160       170       180
                         ....|....|....|....|....|....|....|....|....|....|....|....|
8900-N.crassaA           AAGAAGGTCATCATCTCTGCCCCCTCTGCTGATGCCCCCATGTACGTTATGGGTGTCAAC
8772-N.crassaB           ............................................................
8860-N.crassaC           ............................................................
8835-PS3                 ............................................................
412-N.sitophila          .....A..............T.......................................
8775-N.tetrasperma       ............................................................
8817-PS1                 ............................................................
8901-N.intermediaA       ............................................................
8844-N.intermediaB       ............................................................
8853-PS2                 ............................................................
8827-N.discreta          ............................................................
958-G.calospora          ............................................................
5508-N.sublineolata      ............................................................
959-G.cerealis           ............................................................
7221-N.pannonica         ............................................................
1889-N.terricola         ...............A............................................
1740-N.africana          ............................................................
1739-N.galapagosensis    ............................................................
1692-N.dodgei            ............................................................
1910-N.lineolata         ....................T.......................................
```

```
                                  190       200       210       220       230       240
                         ....|....|....|....|....|....|....|....|....|....|....|....|
8900-N.crassaA           AACGAGACCTACGATGGCTCCGCCGACGTCATCTCCAACGCCTCTTGCACCACCAACTGC
8772-N.crassaB           ............................................................
8860-N.crassaC           ............................................................
8835-PS3                 ............................................................
412-N.sitophila          ............................................................
8775-N.tetrasperma       ............................................................
8817-PS1                 ..............C.........A...................................
8901-N.intermediaA       ..............C.............................................
8844-N.intermediaB       ..........................T.................................
8853-PS2                 ............................................................
8827-N.discreta          ..............C.........A...................................
958-G.calospora          ..............C.........A...................................
5508-N.sublineolata      ..............C.........A...................................
959-G.cerealis           ........................A...................................
7221-N.pannonica         ..............C.........A...................................
1889-N.terricola         ..............C.........A...................................
1740-N.africana          ..............C.........A...................................
1739-N.galapagosensis    ..............C.........A...................................
1692-N.dodgei            ..............C.........A...................................
1910-N.lineolata         ..............C.........A...................................
```

Supplemental Figure. Nucleotide alignment of a part of the coding region of *ccg-7*

```
                                250       260       270       280       290       300
                      ....|....|....|....|....|....|....|....|....|....|....|....|
8900-N.crassaA        TTGGCTCCCCTCGCCAAGGTCATCCACGACAACTTCACCATCGTCGAGGGTCTCATGACC
8772-N.crassaB        ............................................................
8860-N.crassaC        ............................................................
8835-PS3              ........T...................................................
412-N.sitophila       ............................................................
8775-N.tetrasperma    ...........T................................................
8817-PS1              ............................................................
8901-N.intermediaA    ............................................................
8844-N.intermediaB    ............................................................
8853-PS2              ............................................................
8827-N.discreta       ....................TG......................................
958-G.calospora       .........................................T..................
5508-N.sublineolata   .........................................T..................
959-G.cerealis        ..........................................A.................
7221-N.pannonica      ............................................................
1889-N.terricola      ............................................................
1740-N.africana       ..........................................A.................
1739-N.galapagosensis ..........................................A.................
1692-N.dodgei         ..........................................A.................
1910-N.lineolata      ..........................................A.................
```

```
                                310       320       330       340       350       360
                      ....|....|....|....|....|....|....|....|....|....|....|....|
8900-N.crassaA        ACCGTCCACTCCTACACCGCCACCCAGAAGACCGTCGATGGTCCTTCCGCCAAGGACTGG
8772-N.crassaB        ............................................................
8860-N.crassaC        ............................................................
8835-PS3              ............................................................
412-N.sitophila       ..T........T................................................
8775-N.tetrasperma    ..T........T................................................
8817-PS1              ................................................T...........
8901-N.intermediaA    ............................................................
8844-N.intermediaB    ............................................................
8853-PS2              ............................................................
8827-N.discreta       ............................................................
958-G.calospora       ............................................................
5508-N.sublineolata   .................G..........................................
959-G.cerealis        ...A........................................................
7221-N.pannonica      ............................................................
1889-N.terricola      ............................................................
1740-N.africana       ............................................................
1739-N.galapagosensis ............................................................
1692-N.dodgei         ............................................................
1910-N.lineolata      .................T..........................................
```

Supplemental Figure. Nucleotide alignment of a part of the coding region of *ccg-7*

```
                               370       380       390       400       410       420
                       ....|....|....|....|....|....|....|....|....|....|....|....|
8900-N.crassaA         CGCGGTGGCCGCACTGCTGCTCAGAACATCATTCCCAGCAGCACTGGTGCCGCCAAGGCC
8772-N.crassaB         ............................................................
8860-N.crassaC         ............................................................
8835-PS3               ...........................................................T
412-N.sitophila        ............................................................
8775-N.tetrasperma     ............................................................
8817-PS1               ............................................................
8901-N.intermediaA     ............................................................
8844-N.intermediaB     ............................................................
8853-PS2               ............................................................
8827-N.discreta        ............................................................
958-G.calospora        .....................................................T......
5508-N.sublineolata    .....................................................T......
959-G.cerealis         .....................................................T......
7221-N.pannonica       ............................................................
1889-N.terricola       .....................................................T......
1740-N.africana        ...........................................................T
1739-N.galapagosensis  ...........................................................T
1692-N.dodgei          ...........................................................T
1910-N.lineolata       .....................................................T.....T
```

```
                               430       440       450       460       470       480
                       ....|....|....|....|....|....|....|....|....|....|....|....|
8900-N.crassaA         GTCGGCAAGGTCATCCCCGACCTCAACGGCAAGCTCACTGGTATGGCCATGCGTGTCCCC
8772-N.crassaB         ............................................................
8860-N.crassaC         ............................................................
8835-PS3               ............................................................
412-N.sitophila        ............................................................
8775-N.tetrasperma     ............................................................
8817-PS1               ............................................................
8901-N.intermediaA     ...........................................................T
8844-N.intermediaB     ............................................................
8853-PS2               .......................T....................................
8827-N.discreta        ......................................C.....................
958-G.calospora        .........................................C..................
5508-N.sublineolata    .........................................C..................
959-G.cerealis         .........................................C..................
7221-N.pannonica       .................T.......................C...T..............
1889-N.terricola       .........................................C..................
1740-N.africana        .........................................C...T..............
1739-N.galapagosensis  .........................................C...T..............
1692-N.dodgei          .........................................C...T..............
1910-N.lineolata       .........................................C...T..............
```

Supplemental Figure. Nucleotide alignment of a part of the coding region of *ccg-7*

```
                                  490       500       510       520       530       540
                        ....|....|....|....|....|....|....|....|....|....|....|....|
8900-N.crassaA          ACCGCCAACGTCTCCGTTGTCGATCTTACTGCCCGCATCGAGAAGGGTGCTACCTACGAT
8772-N.crassaB          ............................................................
8860-N.crassaC          .............................C..............................
8835-PS3                ............................................................
412-N.sitophila         .............................C..............................
8775-N.tetrasperma      .............................C..............................
8817-PS1                .............................C..............................
8901-N.intermediaA      .............................C..............................
8844-N.intermediaB      .............................C.....T........................
8853-PS2                .............................C..............................
8827-N.discreta         .............................C.T....C.........C....T........
958-G.calospora         .............................C.T....C..............T.......C
5508-N.sublineolata     .............................C.T....C..............T.......C
959-G.cerealis          .............................C.T....C..............T........
7221-N.pannonica        .............................C.T....C..............T........
1889-N.terricola        .............................C.T...................T........
1740-N.africana         .............................C.T....C..............T........
1739-N.galapagosensis   .............................C.T....C..............T........
1692-N.dodgei           .............................C.T....C..............T........
1910-N.lineolata        .............................C.T....C..............T........
```

```
                                  550       560       570       580       590       600
                        ....|....|....|....|....|....|....|....|....|....|....|....|
8900-N.crassaA          GAGATCAAGGAGGTCATCAAGAAGGCCTCTGAGGGTCCCCTCGCTGGCATCCTTGCCTAC
8772-N.crassaB          ...............G............................................
8860-N.crassaC          ..........C....G............................................
8835-PS3                ...............G............................................
412-N.sitophila         ............................................................
8775-N.tetrasperma      ............................................................
8817-PS1                ...............G............................................
8901-N.intermediaA      ............................................................
8844-N.intermediaB      ............................................................
8853-PS2                ............................................................
8827-N.discreta         ...........................G................................
958-G.calospora         ...............G...........G...........A.G..................
5508-N.sublineolata     .........C.....G...........G...........A.G..................
959-G.cerealis          ...............G...........G..........T.....................
7221-N.pannonica        ...........................G................................
1889-N.terricola        ...............G...........G................................
1740-N.africana         ...............G...........G................................
1739-N.galapagosensis   ...............G...........G................................
1692-N.dodgei           ...............G...........G................................
1910-N.lineolata        ...............G...........G................................
```

Supplemental Figure. Nucleotide alignment of a part of the coding region of *ccg-7*

```
                               610       620       630       640       650       660
                       ....|....|....|....|....|....|....|....|....|....|....|....|
8900-N.crassaA         ACCGAGGATGAGGTTGTCTCTTCCGACATGAACGGCAACCCCGCCTCCTCCATCTTCGAT
8772-N.crassaB         ............................................................
8860-N.crassaC         .................T..........................................
8835-PS3               ..T.........................................................
412-N.sitophila        ..T.........................................................
8775-N.tetrasperma     ..T.........................................................
8817-PS1               ..T.........................................................
8901-N.intermediaA     ..T.........................................................
8844-N.intermediaB     ..T.........................................................
8853-PS2               ..T.........................................................
8827-N.discreta        ..T.....C...................................................
958-G.calospora        ..T......................................T..................
5508-N.sublineolata    ..T......................................T..............T...
959-G.cerealis         ..T.....C...................................................
7221-N.pannonica       ..T.....C..............T.......................T............
1889-N.terricola       ..T.....C..............T.......................T............
1740-N.africana        ..T.....C..............T....................................
1739-N.galapagosensis  ..T.....C..............T....................................
1692-N.dodgei          ..T.....C..............T....................................
1910-N.lineolata       ..T.....C..............T....................................
```

```
                               670       680       690       700       710       720
                       ....|....|....|....|....|....|....|....|....|....|....|....|
8900-N.crassaA         GCCAAGGCTGGTATCTCCCTCAACAAGAACTTCGTCAAGCTTGTCTCCTGGTACGACAAC
8772-N.crassaB         ............................................................
8860-N.crassaC         ............................................................
8835-PS3               ............................................................
412-N.sitophila        ........C...................................................
8775-N.tetrasperma     ........C...................................................
8817-PS1               ........C...................................................
8901-N.intermediaA     ........C...................................................
8844-N.intermediaB     ........C...................................................
8853-PS2               ........C...................................................
8827-N.discreta        ............................................................
958-G.calospora        ........C......................................T............
5508-N.sublineolata    ........C........G.............................T............
959-G.cerealis         ........C...............................CA.................
7221-N.pannonica       .........................................C..................
1889-N.terricola       .........................................C..................
1740-N.africana        ........C................................C..................
1739-N.galapagosensis  ........C................................C..................
1692-N.dodgei          ........C................................C..................
1910-N.lineolata       ........C................................C.......C..........
```

Supplemental Figure. Nucleotide alignment of a part of the coding region of *ccg-7*

```
                                   730       740       750
                          ....|....|....|....|....|....|...
8900-N.crassaA            GAGTGGGGCTACTCTCGCCGTGTCCTCGACCTC
8772-N.crassaB            .................................
8860-N.crassaC            .................................
8835-PS3                  .................................
412-N.sitophila           .................................
8775-N.tetrasperma        .................................
8817-PS1                  .................................
8901-N.intermediaA        .................................
8844-N.intermediaB        .................................
8853-PS2                  .................................
8827-N.discreta           .................................
958-G.calospora           .................................
5508-N.sublineolata       .................................
959-G.cerealis            .................................
7221-N.pannonica          .................................
1889-N.terricola          .................................
1740-N.africana           .................................
1739-N.galapagosensis     .................................
1692-N.dodgei             .................................
1910-N.lineolata          .................................
```

Supplemental Figure. Amino acid alignment of a part of GAPDH

```
                                  10        20        30        40        50        60
                         ....|....|....|....|....|....|....|....|....|....|....|....|
8900-N.crassaA           YTERDPAAIPWSETGADYIVESTGVFTTTEKASAHLKGGAKKVIISAPSADAPMYVMGVN
8772-N.crassaB           ---------...................................................
8860-N.crassaC           ............................................................
8835-PS3                 ............................................................
412-N.sitophila          ............................................................
8775-N.tetrasperma       ............................................................
8817-PS1                 ............................................................
8901-N.intermediaA       ............................................................
8844-N.intermediaB       ............................................................
8853-PS2                 ---------...................................................
8827-N.discreta          ............................................................
958-G.calospora          ...........G.................D..............................
5508-N.sublineolata      ...........G.................D..............................
959-G.cerealis           ............................................................
7221-N.pannonica         ............................................................
1889-N.terricola         ......T....A.................................T..............
1740-N.africana          ............................................................
1739-N.galapagosensis    ............................................................
1692-N.dodgei            ............................................................
1910-N.lineolata         .............................D..............................
```

```
                                  70        80        90       100       110       120
                         ....|....|....|....|....|....|....|....|....|....|....|....|
8900-N.crassaA           NETYDGSADVISNASCTTNCLAPLAKVIHDNFTIVEGLMTTVHSYTATQKTVDGPSAKDW
8772-N.crassaB           ............................................................
8860-N.crassaC           ............................................................
8835-PS3                 ............................................................
412-N.sitophila          ............................................................
8775-N.tetrasperma       ............................................................
8817-PS1                 ........N...............................................S...
8901-N.intermediaA       ............................................................
8844-N.intermediaB       ............................................................
8853-PS2                 ............................................................
8827-N.discreta          ........N..................V................................
958-G.calospora          ........N...................................................
5508-N.sublineolata      ........N...................................................
959-G.cerealis           ........N.........................I......I..................
7221-N.pannonica         ........N...................................................
1889-N.terricola         ........N...................................................
1740-N.africana          ........N.........................I.........................
1739-N.galapagosensis    ........N.........................I.........................
1692-N.dodgei            ........N.........................I.........................
1910-N.lineolata         ........N.........................I.........................
```

Supplemental Figure. Amino acid alignment of a part of GAPDH

```
                                 130       140       150       160       170       180
                         ....|....|....|....|....|....|....|....|....|....|....|....|
8900-N.crassaA           RGGRTAAQNIIPSSTGAAKAVGKVIPDLNGKLTGMAMRVPTANVSVVDLTARIEKGATYD
8772-N.crassaB           ............................................................
8860-N.crassaC           ............................................................
8835-PS3                 ............................................................
412-N.sitophila          ............................................................
8775-N.tetrasperma       ............................................................
8817-PS1                 ............................................................
8901-N.intermediaA       ............................................................
8844-N.intermediaB       ............................................................
8853-PS2                 ............................................................
8827-N.discreta          ..................................................V.L..A.S..
958-G.calospora          ..................................................V.L....S..
5508-N.sublineolata      ..................................................V.L....S..
959-G.cerealis           ..................................................V.L....S..
7221-N.pannonica         ...................................S..............V.L....S..
1889-N.terricola         ..................................................V......S..
1740-N.africana          ...................................S..............V.L....S..
1739-N.galapagosensis    ...................................S..............V.L....S..
1692-N.dodgei            ...................................S..............V.L....S..
1910-N.lineolata         ...................................S..............V.L....S..
```

```
                                 190       200       210       220       230       240
                         ....|....|....|....|....|....|....|....|....|....|....|....|
8900-N.crassaA           EIKEVIKKASEGPLAGILAYTEDEVVSSDMNGNPASSIFDAKAGISLNKNFVKLVSWYDN
8772-N.crassaB           .....V......................................................
8860-N.crassaC           ...A.V......................................................
8835-PS3                 .....V......................................................
412-N.sitophila          ............................................................
8775-N.tetrasperma       ............................................................
8817-PS1                 .....V......................................................
8901-N.intermediaA       ............................................................
8844-N.intermediaB       ............................................................
8853-PS2                 ............................................................
8827-N.discreta          .........A..................................................
958-G.calospora          .....V...A...M..............................................
5508-N.sublineolata      ...Q.V...A...M..............................................
959-G.cerealis           .....V...A.............................................I.....
7221-N.pannonica         .........A..................................................
1889-N.terricola         .....V...A..................................................
1740-N.africana          .....V...A..................................................
1739-N.galapagosensis    .....V...A..................................................
1692-N.dodgei            .....V...A..................................................
1910-N.lineolata         .....V...A...............................................S...
```

Supplemental Figure. Amino acid alignment of a part of GAPDH

```
                                       250
                               ....|....|.
8900-N.crassaA                 EWGYSRRVLDL
8772-N.crassaB                 ...........
8860-N.crassaC                 ...........
8835-PS3                       ...........
412-N.sitophila                ...........
8775-N.tetrasperma             ...........
8817-PS1                       ...........
8901-N.intermediaA             ...........
8844-N.intermediaB             ...........
8853-PS2                       ...........
8827-N.discreta                ...........
958-G.calospora                ...........
5508-N.sublineolata            ...........
959-G.cerealis                 ...........
7221-N.pannonica               ...........
1889-N.terricola               ...........
1740-N.africana                ...........
1739-N.galapagosensis          ...........
1692-N.dodgei                  ...........
1910-N.lineolata               ...........
```
